# Trivalent Metal Lewis Acids Activate CO_2_ in Transfer Hydrogenations

**DOI:** 10.1002/cssc.202500629

**Published:** 2025-07-09

**Authors:** Alexandros Paparakis, Leandro D. Mena, Pritha Saha, Krishna Mohan Das, Daniel Shirwani, Jorge G. Uranga, Martin Hulla

**Affiliations:** ^1^ Department of Inorganic Chemistry Faculty of Science Charles University Albertov 6 128 00 Praha 2 Czech Republic; ^2^ Departamento de Química Orgánica Facultad de Ciencias Químicas Universidad Nacional de Córdoba (INFIQC‐CONICET) X5000HUA Córdoba Argentina; ^3^ Instituto de Investigaciones en Físico‐Química de Córdoba Universidad Nacional de Córdoba (INFIQC‐CONICET) X5000HUA Córdoba Argentina

**Keywords:** carbon capture and utilizations, CO_2_ reduction to formate, Lewis acids, *p* ‐block catalysts, transfer hydrogenations

## Abstract

Using γ‐terpinene as a bio‐derived H_2_ surrogate, trivalent metal MX_3_ (M = Al, Ga, In, Yb, X = Cl, OTf) Lewis acids (LAs) catalyze CO_2_ hydrogenation to formate, yielding *p*‐cymene as the by‐product. The resulting formate produces up to 91% *N*‐formamides in tandem hydrogenation‐coupling reactions and up to 95% heterocycles, including azoles, via further in situ transfer formylation to *ortho*‐substituted anilines and cyclization at 130 °C and 4 bar. But In(OTf)_3_ and a Lewis base fail to abstract a hydride from γ‐terpinene. Unlike other LAs and transfer hydrogenation catalysts that induce hydride abstraction from 1,4‐cyclohexadiene(s) over B(C_6_F_5_)_3_, alkali earth or noble metals, MX_3_ LAs activate CO_2_, so CO_2_ can directly accept a hydride from γ‐terpinene during formate synthesis, as shown by density functional theory calculations. This triple role of MX_3_ LAs in promoting (1) CO_2_ activation, (2) tandem coupling reactions, and (3) transfer formylation at low pressure paves the way for sustainable CO_2_ hydrogenation processes, leveraging bio‐derived H_2_ surrogates to develop efficient carbon capture and utilization systems and to synthesize valuable compounds from renewable feedstocks.

## Graphical abstract

Indium(III) triflate and other trivalent metal ions catalyst the transfer hydrogenation of CO_2_ to formate and a series of in situ tandem reactions to yield formamides or azoles. In contrast to previous reports, the reaction proceeds by CO_2_ activation instead of hydride abstraction from a transfer hydrogenation source.

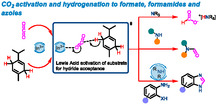

Achieving long‐term sustainability goals requires pivoting chemical synthesis from nonrenewable sources of fossil carbon (coal, oil, and natural gas) to renewable, biomass‐based carbon sources and CO_2_. These renewable technologies often involve CO_2_ reduction to lower oxidation states.^[^ ^1^, ^2^ ^]^ For this purpose, H_2_ is the most atom‐economic reductant. However, green H_2_ production entails high costs, high‐pressure plants, and specialized equipment.^[^ ^3^, ^4^, ^5^ ^]^ These downsides severely limit its practical applications, demanding more efficient production alternatives.

Among potential alternatives, transfer hydrogenations (THs)^[^ ^6^ ^]^ stand out for producing value‐added coproducts instead of undesirable reaction waste. For example, glycerol is a bio‐derived alcohol reductant for CO_2_^[^ ^6^ ^]^ or γ‐terpinene is a biomass‐derived reductant found in plants, such as *Cuminum cyminum L.* and *Melaleuca alternifolia*.^[^ ^7^, ^8^, ^9^ ^]^ γ‐Terpinene and related 1,4‐cyclohexadienes act as reductants in alkene, imine, silyl enol ether and 2‐alkynyl enones^[^ ^10^, ^11^ ^]^ THs over Lewis acid (LA),^[^ ^10^, ^11^, ^12^, ^13^, ^14^ ^]^ AeN″_2_ (Ae = Ca, Sr, Ba, N = Bis(trimethylsilyl)amine),^[^ ^15^ ^]^ ruthenium,^[^ ^16^ ^]^ or Brønsted super acid catalysts^[^ ^12^ ^]^ with *p*‐cymene coproduction. In turn, *p*‐cymene is a flavoring agent, a common ligand in transition metal (TM) chemistry and a sustainable alternative solvent to toluene and *para*‐xylene.^[^ ^17^, ^18^, ^19^, ^20^ ^]^ Through hydride abstraction from γ‐terpinene, these THs yield acid‐sensitive LA‐H or TM‐H reductants and a highly Brønsted acidic Wheland intermediate, as shown computationally^[^ ^13^, ^15^ ^]^ and experimentally^[^ ^16^, ^21^ ^]^ (**Scheme** **1**A). But without a sufficiently reactive substrate, H_2_ is released.^[^ ^16^ ^]^ As a result, these methods fail in CO_2_ TH reactions.

**Scheme 1:**
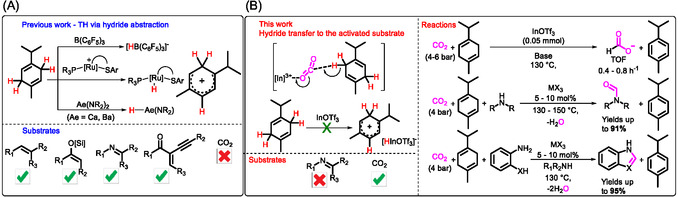
A) Previous THs with γ‐terpinene and related 1,4‐cyclohexadienes proceeding by hydride abstraction. B) This work: TH resulting from substrate activation with [In]^3+^ and other M^3+^ promotes CO_2_ TH to formate and its reductive coupling to amines and transfer formylation, yielding azoles.

In this study, we demonstrate that CO_2_ TH with γ‐terpinene to formate can be catalyzed by combining commercially available indium(III) triflate with a suitable base. We found that In(OTf)_3_ and other simple metal salts, such as InCl_3_, Al(OTf)_3_, Ga(OTf)_3_, and Yb(OTf)_3_, also catalyze CO_2_ reductive coupling with aliphatic amines, yielding formamides, and the tandem transfer formylation to *ortho*‐substituted anilines, yielding azoles (Scheme 1B). Moreover, the reactions proceed via CO_2_ activation by the LA catalyst (Scheme 1B), not via hydride abstraction from γ‐terpinene. Therefore, our findings contrast with the mechanism of LA of lighter elements, alkali earth metals, and TMs.

First, mixing 1,8‐diazabicyklo[5.4.0]undec‐7‐en (DBU) with a catalytic amount of In(OTf)_3_ in the presence of γ‐terpinene and CO_2_ (4–10 bar) at 130 °C (**Table** **1**) produced [DBUH][formate]. At 4 bar, the yield increased linearly with time, from 0.95 mmol after 48 h (Table 1, entry 1) to 1.45 mmol after 72 h (Table 1, entry 2), thus indicating negligible catalyst deactivation. Increasing the CO_2_ pressure to 6 bar doubled the turnover frequency (TOF) to 0.81 h^−1^ (Table 1, entry 3), but further increasing this pressure to 10 bar decreased the TOF to 0.37 h^−1^, perhaps because DBU tends to form carbamates.^[^ ^22^ ^]^ These carbamates can bind to In(OTf)_3_, slowing down the reaction.

**Table 1:** CO_2_ pressure affects the synthesis of DBU formate.

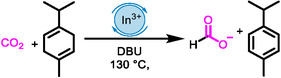					
Entry	Temperature [°C]	CO_2_ [bar]	Time [h)]	[Formate]‐[mmol]	TOF [h^−1^]
1	130	4	48	0.95	0.40
2	130	4	72	1.45	0.40
3	130	6	48	1.94	0.81
4	130	10	48	0.88	0.37
5a)	130	4	48	0	0

Reaction conditions: In(OTf)_3_ (0.05 mmol). The reaction solvent was a 1:1:2 mixture of DBU: γ‐terpinene: DMSO. Average yield after three runs. The yields were determined by ^1^H NMR with an internal standard based on the amount of [DBUH][OCOH] present in the reaction. All materials were used as received without further drying or purification.

a) Without In(OTf)_3_.

Second, formates produced in this reaction can be directly in situ applied in organic synthesis to prepare formamides via coupling with amines, among other reactions. Because LAs such as In(OTf)_3_ catalyze these reactions,^[^ ^23^, ^24^ ^]^ CO_2_ may be reductively coupled to amines, yielding formamides through tandem synthesis (**Table** **2**). Using morpholine as the model *N*‐formylation substrate with In(OTf)_3_ (10 mol%) and a mixture of γ‐terpinene, DBU, and dimethylsulfoxide (DMSO) as the reaction solvent, we obtained *N*‐formylmorpholine in 26% yield (Table 2, entry 1). In addition, the reaction produced 0.6 mmol of [DBUH][formate]. Accordingly, this reaction is limited by the *N*‐formylation step rather than by CO_2_ reduction.

**Table 2:** LAs and bases enable CO_2_ TH and reductive coupling to amines.

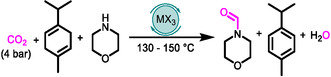				
Entry	Lewis acid	Lewis Base	Temperature [°C]	Yield [%]
1	In(OTf)_3_	DBU	130	26
2	In(OTf)_3_	NMM	130	65
3	In(OTf)_3_	2,4,6‐ Col	130	31
4	In(OTf)_3_	DABCO	130	13
5	InCl_3_	NMM	130	31
6	Ga(OTf)_3_	NMM	130	56
7	Al(OTf)_3_	NMM	130	32
8	Yb(OTf)_3_	NMM	130	30
9	In(OTf)_3_	NMM	150	91
10	In(OTf)_3_ a)	NMM	150	45

Reaction conditions: Morpholine (1 mmol), LA (0.1 mmol = 10 mol%), CO_2_ (4 bar), 48 h, average yield after three runs. Yields were determined by ^1^H NMR with an internal standard based on the amount of *N*‐formyl morpholine present in the reaction.

a) 5 mol% of catalyst instead of 10 mol%. All materials were used as received without further drying or purification.

Based on these findings, we hypothesized that DBU was too basic for proton transfer during morpholine condensation with [DBUH][formate]. Substituting DBU for *N*‐methylmorpholine (NMM, pKa of conjugate acid 7.38 in H_2_O) improved the reaction yield to 65% (Table 2, entry 2), and ex situ synthesis of [DBUH][formate] and [‐N‐methylmopholinium][formate] confirmed that the *N*‐methylmorpholinium salt is a better *N*‐formylation reagent than [DBUH][formate] (SI section 5, Supporting Information). However, the aromatic base 2,4,6‐collidine (pKa 7.43 of conjugate acid in H_2_O) with similar basicity to NMM did not significantly improve the yield in comparison with DBU, with only 31% yield of *N*‐formylmorpholine (Table 2, entry 3) due to its poor miscibility with DMSO, the reaction solvent. The use of stronger coordinating bases such as 1,4‐Diazabicyclo[2.2.2]octane (DABCO, pKa of conjugate acid 8.8 in H_2_O) decreased the *N*‐formylmorpholine yield to 13% (Table 2, entry 4), indicating that balancing the base strength and the coordination environment of indium is crucial for the reaction. Replacing indium(III) triflate with InCl_3_ also resulted in 31% yield (Table 2, entry 5), indicating that triflate dissociation and indium ion coordination play key roles in the reaction. Substituting indium for other trivalent metal triflates decreased the reaction yield in the following order: In > Ga > Al > Yb (Table 2, entries 1 and 6–8). Upon further reaction temperature, time, and solvent optimization, we obtained the desired *N*‐formylmorpholine in 91% yield over In(OTf)_3_, which was the best catalyst (Table 2, entry 9). In addition, in line with the linear kinetics halving the catalyst loading reduced the product yield from 91% to 45% (Table 2, entry 10). Further kinetic analysis showed negative rate order with respect to CO_2_ at pressures above 6 bar, which is consistent with reaction inhibition by carbamate salt formation and negative rate order with respect to morpholine concentration indicative of catalyst deactivation by morpholine binding to the In^3+^ cation (SI, section 9, Supporting Information) or potential homogenity issues due to poor solubility of carbamate salts formed by the reaction of morpholine with CO_2_.

Under optimized conditions, we tested various substrates (**Scheme** **2**A). Dialkyl amines, including morpholine (1), dimethylamine (used as the CO_2_ adduct dimethylammonium dimethyl carbamate) (2), *N*‐methylpiperazine (3), and pyrrolidine (4), were *N*‐formylated in yields ranging from 41 to 91%. However, substrates 2–4 required a lower catalyst loading to proceed, highlighting difficulties in tandem synthesis and explaining the lower overall formamide yield. The lower overall yield may be explained by formate salt decomposition in the presence of In(OTf)_3_, as formic acid/formate can be dehydrogenated to H_2_ and CO_2_ (SI section 7, Supporting Information). In(OTf)_3_ dehydrogenates formic acid even in the presence of 4 bars of CO_2_ (SI section 7, Supporting Information), showing that lowered catalyst loadings disfavor the LA‐catalyzed decomposition pathway, allowing the *N*‐formylation step to proceed. Low boiling point substrates with high vapor pressures, such as 2 and 4, may also lead to mass transfer limitations of the *N*‐formylation step due to substrate evaporation, allowing for the formate salt decomposition prior to amine *N*‐formylation. Hence, the remainder of the substrate scope was run at 5 mol% catalyst loading.

**Scheme 2:**
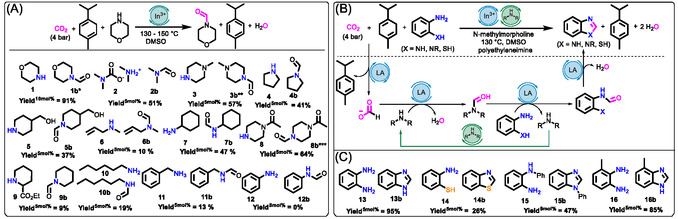
A) Substrate scope for *N‐*formylation with CO_2_ and γ‐terpinene over In(OTf)_3_. Reaction conditions: amine (1 mmol), DMSO (2 mL), NMM (1 mL), γ‐terpinene (1 mL), LA (5 mol%), and CO_2_ (4 bar) at 130 °C for 48 h. The yields were determined by ^1^H NMR, and the structures were confirmed by gas chromatography coupled to mass spectrometry (GC‐MS). * Reaction run for 24 h at 150 °C; ** at 130 °C and with 6 bar CO_2_; and ***, at 6 bar CO_2_. B) Direct in situ synthesis of azoles from *ortho*‐substituted anilines, CO_2_, and γ‐terpinene facilitated by secondary amines (in this case polyethyleneimine MW = 1200, which is represented by the green R_2_NH symbol) and catalyzed by In(OTf)_3_ via CO_2_ TH with γ‐terpinene, formate coupling to secondary amine, formate transfer to *ortho*‐substituted aniline, and cyclization to an azole. C) Substrate scope for the synthesis of azoles under conditions equivalent to (A) with 0.1 mL of polyethyleneimine (MW = 1200).

With this catalyst loading, the system was functionally tolerant of alcohols (5) and alkenes (6), with no evidence of concomitant reduction of the double bond. Most primary amines remained unreactive in the presence of In(OTf)_3_, and only cyclohexylamine (7), hexylamine (10), and benzylamine (11) produced measurable quantities of *N*‐formylated products with 47, 19%, and 13% yield, respectively. For 3 and 8, the *N*‐formylation reactions were performed at a lower temperature (130 °C) and higher CO_2_ pressure (6 bar) because we found that DMSO acted as a C1 source in competing *N*‐methylation reaction, which is catalyzed by formate formed by CO_2_ hydrogenation.^[^ ^25^ ^]^ Under these conditions, we reached 57% and 64% yields for 3b and 8b, respectively. During the reaction, the ester functionality of substrate 9 was completely removed by the In(OTf)_3_ (SI, section 8, Supporting Information), leading to only 10% yield of 9b. In addition, 10 was selectively mono‐formylated. Conversely, 11 yielded a mixture of products primarily consisting of the mono‐formylated product under standard conditions. Aniline (12) remained unreactive to *N*‐formylation.

Third, In(OTf)_3_ was compatible with and catalyzed the sequential tandem synthesis of azoles through four consecutive LA‐promoted reactions (Scheme 2B,C). Azole synthesis proceeds over In(OTf)_3_ promoted CO_2_ TH to formate (Table 1), the *N*‐formylation of the secondary amine (Table 2), LA‐catalyzed formate transfer from formamide to *ortho*‐substituted aniline^[^ ^26^, ^27^, ^28^ ^]^ and its LA‐catalyzed cyclization,^[^ ^29^ ^]^ yielding between 26% and 95% of the desired azole (13b–16b) using polyethyleneimine as the in situ recycled secondary amine scaffold. However, attempts to extend the scope beyond azoles to other heterocycles were unsuccessful.

To better understand the mechanism of CO_2_ TH, we performed density functional theory (DFT) calculations at the ωB97XD/def2TZVP//ωB97XD/6‐31+G(d) level of theory (details in SI, Supporting Information). When dissolving In(OTf)_3_ in DMSO, triflate ligands may be readily replaced by DMSO molecules, forming the hexa‐coordinate In(DMSO)_6_ complex^[^ ^30^, ^31^ ^]^ in line with our DFT calculations (Figure S22, Supporting Information). Given that triflates should not significantly affect the catalytic activity, we optimized the geometry of the hexa‐coordinate In(DMSO)_6_ as the starting structure. The optimized geometry corroborated previous reports, showing In—O bond lengths between 2.14 and 2.22 Å.^[^ ^30^ ^]^ Importantly, one axial DMSO molecule can be replaced by other species, such as CO_2_ or NMM, which are critical for the reaction and were considered in our analysis (S9.2 in SI, Supporting Information)

Khan et al. reported that TH begins with hydride transfer from γ‐terpinene to the boron atom over the B(C_6_F_5_)_3_ catalyst. Comparable hydride abstraction steps were reported for AeN″_2_ (Ae = Ca, Sr, Ba, and N = bis(trimethylsilyl)amine)^[^ ^15^ ^]^ and ruthenium catalysts.^[^ ^16^ ^]^ Experimentally, this mechanism is supported by H_2_ release from γ‐terpinene over B(C_6_F_5_)_3_ via H‐B(C_6_F_5_)_3_ protonation with the concomitantly formed Wheland intermediate. However, in our system, attempts to model hydride transfer from γ‐terpinene to the indium center were unsuccessful. Moreover, In(OTf)_3_ alone, or in combination with DBU or NMM, it did not catalyze γ‐terpinene dehydrogenation to *p*‐cymene and H_2_, nor did it promote TH of substrates like imines, which are known to proceed via hydride abstraction.^[^ ^12^, ^15^, ^16^, ^21^ ^]^ These results rule out the hydride abstraction mechanism that is well supported for frustrated Lewis pair, alkali earth, and TM catalysts.^[^ ^12^, ^15^, ^16^, ^21^ ^]^ Instead, our findings suggest that CO_2_ may accept the hydride directly from γ‐terpinene, with DBU or NMM acting as the proton acceptor (**Scheme** **3**). The generated product complex (PC) features a stabilizing formate ligand, which may dissociate as [base][formate] ion pair to regenerate the In^3+^ catalyst and separated products (SP), making it available to initiate a new catalytic cycle. In this mechanism, hydride transfer is the rate‐limiting step, with an activation energy of 42.9 kcal mol^−1^ without an In(OTf)_3_ catalyst with NMM or 45.2 kcal mol^−1^ with DBU.

**Scheme 3:**
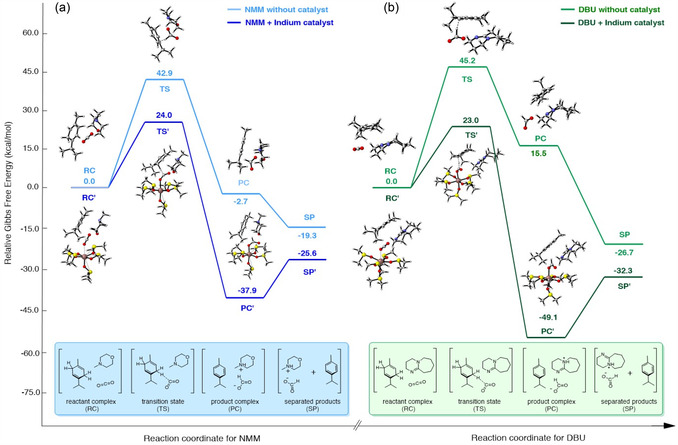
a) Energy profile of uncatalyzed (light blue) and catalyzed (blue) TH reactions involving γ‐terpinene as the H_2_ surrogate, NMM as the base, and CO_2_, calculated at the ωB97XD/def2TZVP//ωB97XD/6‐31 + G(d,p) level of theory. The Gibbs free energies are expressed relative to the energy of the initial reaction complex (RC or RC′). b) Coordinates using DBU as the base instead of NMM, calculated at the same level of theory.

Although proton transfer from the Wheland intermediate to NMM occurs without a major barrier (see Figure S21, Supporting Information) and the overall reaction is slightly exergonic (−2.7 kcal mol^−1^), the high activation energy for hydride transfer accounts for the lack of reactivity without the catalyst. Over the indium catalyst with CO_2_ coordinated to its vacant site (RC’ to PC’, Scheme 3a), the activation energy drops significantly to 24.0 kcal mol^−1^, and the reaction Gibbs free energy (Δ*G*_r_) decreases to −37.9 kcal mol^−1^. This reduction highlights the key role of the indium catalyst in stabilizing both the transition state and the formate intermediate generated by hydrogenation, in line with experimental observations (Table 1). Additionally, it is noteworthy that in the presence of the catalyst, the C—H bond length in γ‐terpinene undergoing cleavage at the transition state (TS) geometry is considerably shorter (1.28 Å) compared to the uncatalyzed reaction (1.54 Å, Figure S22, Supporting Information), suggesting that the catalyst facilitates an earlier hydride transfer to the activated and hence more electrophilic CO_2_. In effect, the In^3+^ activated CO_2_ can accept a hydride from a shorter (stronger) C—H bond than nonactivated CO_2_ in the absence of the catalyst.

Experimental data also revealed that morpholine formylation is almost three times slower when using DBU as the base instead of NMM (Table 2). To understand this difference, we modeled the mechanism of TH with DBU as the proton acceptor (Scheme 3b). As in the NMM system, the indium catalyst reduced the Δ*G* ^‡^ of the DBU‐based reaction pathway from 45.2 to 23.0 kcal mol^−1^. Additionally, the PC is even more stabilized, with Δ*G*_r_ decreasing to −49.1 kcal mol^−1^, in contrast to the endergonic profile of the uncatalyzed reaction (+15.5 kcal mol^−1^). The PC is 11.2 kcal mol^−1^ more stable in the DBU catalytic system than in the NMM one, making it less reactive in the follow‐up tandem reactions. This finding aligns with the experimental results, according to which formylation slows down when using DBU as the base.

In conclusion, MX_3_ LAs, particularly In(OTf)_3_, effectively catalyze CO_2_ THs with γ‐terpinene to formate in the presence of a Lewis base. In this system, γ‐terpinene functions as a bio‐derived H_2_ surrogate, generating *p*‐cymene as the by‐product. In situ generated formate can be transferred to amines and further to *ortho*‐substituted anilines in LA‐catalyzed tandem reactions, if so desired, yielding formamides and azoles, respectively. Moreover, our system is functionally tolerant and selective to CO_2_ hydrogenations over other functional groups, such as amides and alkenes, with the best catalyst, In(OTf)_3_, producing 1.94 mmol of formate at 6 bar of CO_2_ within 48 h. Supported by DFT calculations, the reaction mechanism involves the dissociation of a solvent molecule from the trivalent metal center, which subsequently binds to CO_2_, directly activating CO_2_ for hydride transfer from γ‐terpinene. This mechanism contrasts with γ‐terpinene‐based THs in which LAs act as the hydride acceptor. This direct CO_2_ activation may facilitate the development of new catalytic systems for H_2_‐free CO_2_ hydrogenations using *p*‐block and lanthanide catalysts while simultaneously yielding useful coproducts.

## Conflict of Interest

The authors declare no conflict of interest.

## Supporting information

Supplementary Material

## Data Availability

The data that support the findings of this study are available in the supplementary material of this article.

## References

[1] Q. Liu , L. Wu , R. Jackstell , M. Beller , Nat. Commun. 2015, 6, 1. 10.1038/ncomms6933 25600683

[2] G. A. Olah , Angew. Chem., Int. Ed. 2005, 44, 2636. 10.1002/anie.200462121 15800867

[3] F. Frieden , J. Leker , Sustainable Energy Fuels 2024, 8, 1806.

[4] U. Y. Qazi , Energies 2022, 15, 4741.

[5] M. Ball , M. Wietschel , Int. J. Hydrogen Energy 2009, 34, 615.

[6] A. Kumar , R. Bhardwaj , S. K. Mandal , J. Choudhury , ACS Catal. 2022, 12, 8886.

[7] N. Khan , S. Ahmed , M. A. Sheraz , Z. Anwar , I. Ahmad , Profiles Drug Subst., Excipients, Relat. Methodol. 2023, 48, 167. 10.1016/bs.podrm.2022.11.006 37061274

[8] N. S. Iacobellis , P. Lo Cantore , F. Capasso , F. Senatore , J. Agric. Food Chem. 2005, 53, 57. 15631509 10.1021/jf0487351

[9] R. Li , Z. T. Jiang , Flavour Fragrance J. 2004, 19, 311.

[10] I. Khan , B. G. Reed‐Berendt , R. L. Melen , L. C. Morrill , Angew. Chem., Int. Ed. 2018, 57, 12356. 10.1002/anie.201808800 PMC6207922 30106498

[11] L. Li , S. Kail , S. M. Weber , G. Hilt , Angew. Chem., Int. Ed. 2021, 60, 23661. 10.1002/anie.202109266 PMC8597135 34476880

[12] I. Chatterjee , M. Oestreich , Org. Lett. 2016, 18, 2463. 27181437 10.1021/acs.orglett.6b01016

[13] I. Chatterjee , Z. W. Qu , S. Grimme , M. Oestreich , Angew. Chem., Int. Ed. 2015, 54, 12158. 10.1002/anie.201504941 26418183

[14] L. Li , G. Hilt , Chem. Eur. J. 2021, 27, 11221. 34048092 10.1002/chem.202101259 PMC8453857

[15] H. Bauer , K. Thum , M. Alonso , C. Fischer , S. Harder , Angew. Chem., Int. Ed. 2019, 58, 4248. 10.1002/anie.201813910 30667149

[16] A. Lefranc , Z. W. Qu , S. Grimme , M. Oestreich , Chem. Eur. J. 2016, 22, 10009. 27311877 10.1002/chem.201600386

[17] C. M. Alder , J. D. Hayler , R. K. Henderson , A. M. Redman , L. Shukla , L. E. Shuster , H. F. Sneddon , Green Chem. 2016, 18, 3879.

[18] X. L. Cao , M. Sparling , R. Dabeka , J. Sci. Food Agric. 2019, 99, 5606. 31206173 10.1002/jsfa.9854

[19] M. M. Vinogradov , Y. N. Kozlov , D. S. Nesterov , L. S. Shul'pina , A. J. L. Pombeiro , G. B. Shul'pin , Catal. Sci. Technol. 2014, 4, 3214.

[20] E. Klaimanee , T. Nhukeaw , S. Saithong , A. Ratanaphan , S. Phongpaichit , Y. Tantirungrotechai , N. Leesakul , Polyhedron 2021, 204, 115244.

[21] I. Chatterjee , M. Oestreich , Angew. Chem., Int. Ed. 2015, 54, 1965. 10.1002/anie.201409246 25529119

[22] X. C. Chen , K. C. Zhao , Y. Q. Yao , Y. Lu , Catal. Sci. Technol. 2021, 11, 7072.

[23] D. Wei , C. Cui , Z. Qu , Y. Zhu , M. Tang , J. Mol. Struct. THEOCHEM 2010, 951, 89.

[24] A. Chandra Shekhar , A. Ravi Kumar , G. Sathaiah , V. Luke Paul , M. Sridhar , P. Shanthan Rao , Tetrahedron Lett. 2009, 50, 7099.

[25] B. N. Atkinson , J. M. J. Williams , ChemCatChem 2014, 6, 1860.

[26] J. Zhu , Z. Zhang , C. Miao , W. Liu , W. Sun , Tetrahedron 2017, 73, 3458.

[27] A. Paparakis , M. Hulla , ChemCatChem 2023, 15, e202300510.

[28] H. Mostafavi , M. R. Islami , E. Ghonchepour , A. M. Tikdari , Chem. Pap. 2018, 72, 2973.

[29] M. Hulla , S. Nussbaum , A. R. Bonnin , P. J. Dyson , Chem. Commun. 2019, 55, 13089. 10.1039/c9cc06156h 31608908

[30] P. Nava , Y. Carissan , S. Humbel , Phys. Chem. Chem. Phys. 2009, 11, 7130. 19672521 10.1039/b907229b

[31] A. Molla‐Abbassi , M. Skripkin , M. Kritikos , I. Persson , J. Mink , M. Sandström , Dalton Trans. 2003, 9, 1746.

