# Supplementary material for: Trivalent Metal Lewis Acids Activate CO2 in Transfer Hydrogenations
Source: ChemSusChem. 2025 Jul 9;18(16):e202500629. doi: 10.1002/cssc.202500629 (PMC12330308; doi:10.1002/cssc.202500629)
Supplement: Supplementary file 1 — Supplementary Material [file CSSC-18-e202500629-s001.pdf]

Supporting information

Contents

1. General procedures 3
2. Catalytic tests 3
   2.1 General Procedure: N-formylation of amines 3
   2.2 Typical result(s) of synthesis of N-formylmorpholine: 4
   2.3 Example of reproducibility between runs: 6
3. Solvent and temperature optimization 10
4. 4. Substrate scope: N-formylation 10
   4.1 Dimethyl formamide 10
   4.2 4-methylpiperazine-1-carbaldehyde 11
   4.3 Pyrrolidine-1-carbaldehyde 12
   4.4 4-(hydroxymethyl)piperidine-1-carbaldehyde 13
   4.5 N-allyl-N-methylformamide 14
   4.6 N-cyclohexylformamide 15
   4.7 4-acetylpiperazine-1-carbaldehyde 16
   4.8 n-Hexylformamide 17
   4.9 N-benzylformamide 18
4 Synthesis of azoles 19
   4.10 Benzimidazole 19
   4.11 Benzothiazole 20
   4.12 N-phenylbenzimidazole 22
   4.13 4-methylbenzimidazole 23
5. Direct formylation of morpholine using DBU formate and N-methyl morpholinium formate. 24
   5.1 General Procedure: 24
6. Synthesis of [DBUH][formate] 25
   6.1 General Procedure for the synthesis of [DBUH][formate]: 25
   6.2: Effect of CO_2 pressure on synthesis of DBU formate 27

7. Formic acid decomposition. 27
7.1 General Procedure: 27
8. Formation of partially deuterated N-methylamines. 28
8.1 Results: 28
9. Computational details 29
9.1. Methods 29
9.2. Starting structures 29
9.3. IRC profile of an uncatalysed reaction 31
9.4. Geometries of the relevant TSs 33
10. Effect of $In(OTf)_3$ on the ^{13}C NMR of CO_2 in DMSO-d6 52
References 55

1. General procedures

All reagents and solvents were purchased from commercial suppliers (Sigma-Aldrich, Merck, Alpha-Aesar, TCI, Across, abcr, Thermo scientific, Linde gas a.s. and Lach:ner). Specifically, Indium triflate was purchased from sigma aldrich. Synthetic reagents were used as received and without further purification including hydrogen gas (99.90%) and carbon dioxide for food industry (99.90%) purchased from Linde gas a.s. All solvents were used as received. Unless otherwise specified ^{1}H NMRs were taken on a Bruker AVANCE-III (400 MHz) at 298 K spectrometer and reported in ppm (δ). Deuterated solvents were purchased from abcr and used as received. NMR spectroscopy abbreviations: s, singlet; d, doublet; t, triplet; m, multiplet. All the products of the catalytic tests were identified by ^{1}H NMR data in comparison with literature, by GC coupled to mass spectrometry on a Shimadzu QP-2010 GC-MS with a Supelcowax 10 column and where necessary by comparison with genuine samples of the targeted compounds.

2. Catalytic tests

2.1 General Procedure: N-formylation of amines

In air $In(OTf)_3$ (0.05 mmol) and morpholine (1 mmol) were dissolved in the solvent mixture (4 mL) in a stainless-steel autoclave. The autoclave was then sealed and purged 5 times with the desired pressure of CO_2. The temperature and stirring rate were set using the Specview program on Parr 5000 series multi reactor system. T = 0 was defined as the time the heating starts. The heating was turned off at T = end of the stated reaction time and immediately cooled down i.e., for a reaction time of 24 hours the heating was turned off after 24 hours, removed from the heating mantel and cooled immediately. After which, dibromomethane (1 mmol) was added to the reactor, stirred and an aliquot was taken for ^{1}H NMR analysis in $CDCl_3$ or DMSO-d_6. The conversion of morpholine and the yield of N-formylmorpholine were quantified by ^{1}H NMR analysis with the added dibromomethane as the internal standard. Other reaction products were quantified by their respective C1 hydrogen signal in ^{1}H NMR and structures confirmed by GC-MS on a Shimadzu QP-2010 GC-MS with a Supelcowax 10 column.

2.2 Typical result(s) of synthesis of N-formylmorpholine:

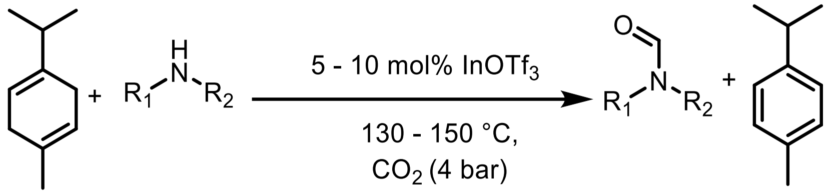

Figure S1A: ^{1}H NMR of a reaction mixture at the end of an N-formylation catalytic test.

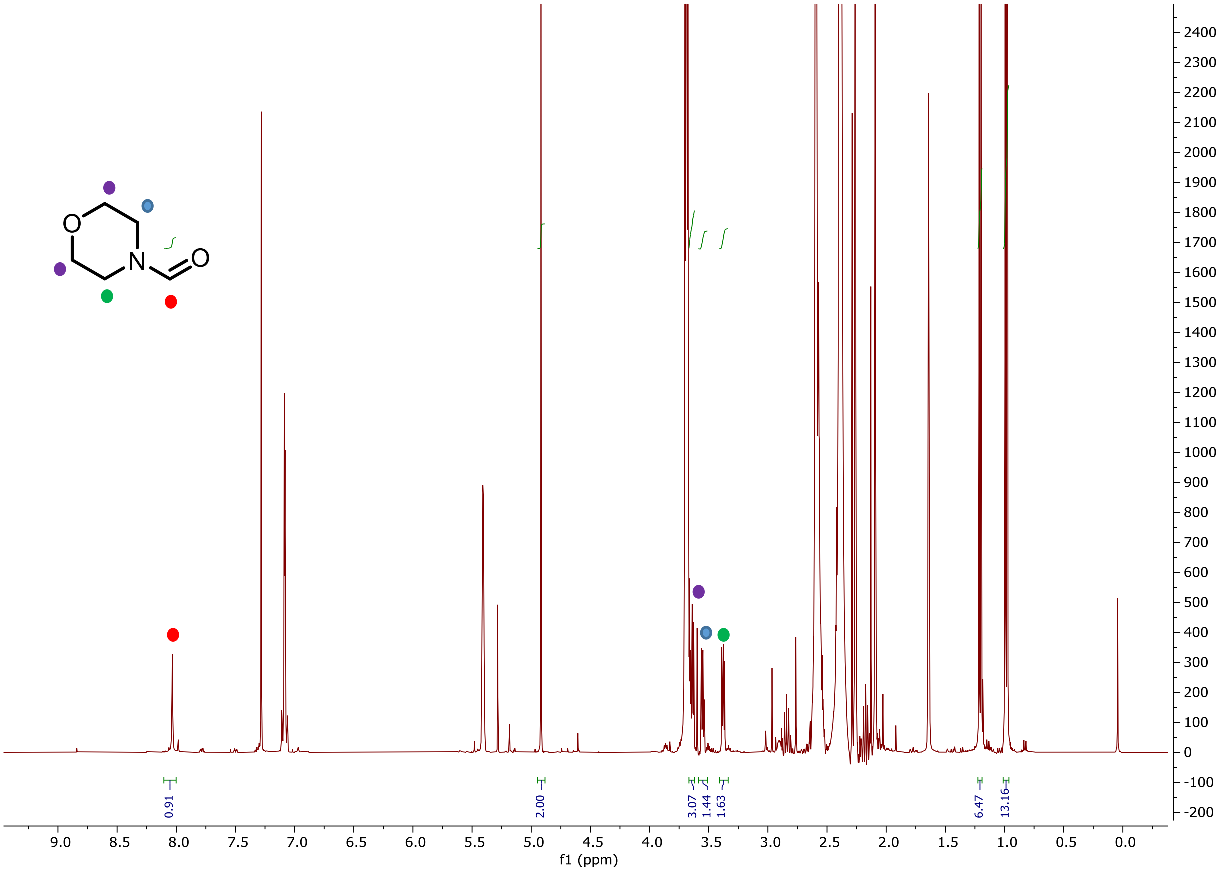

Figure S1B: ^{1}H NMR of the isolated N-formylmorpholine product taken in DMSO

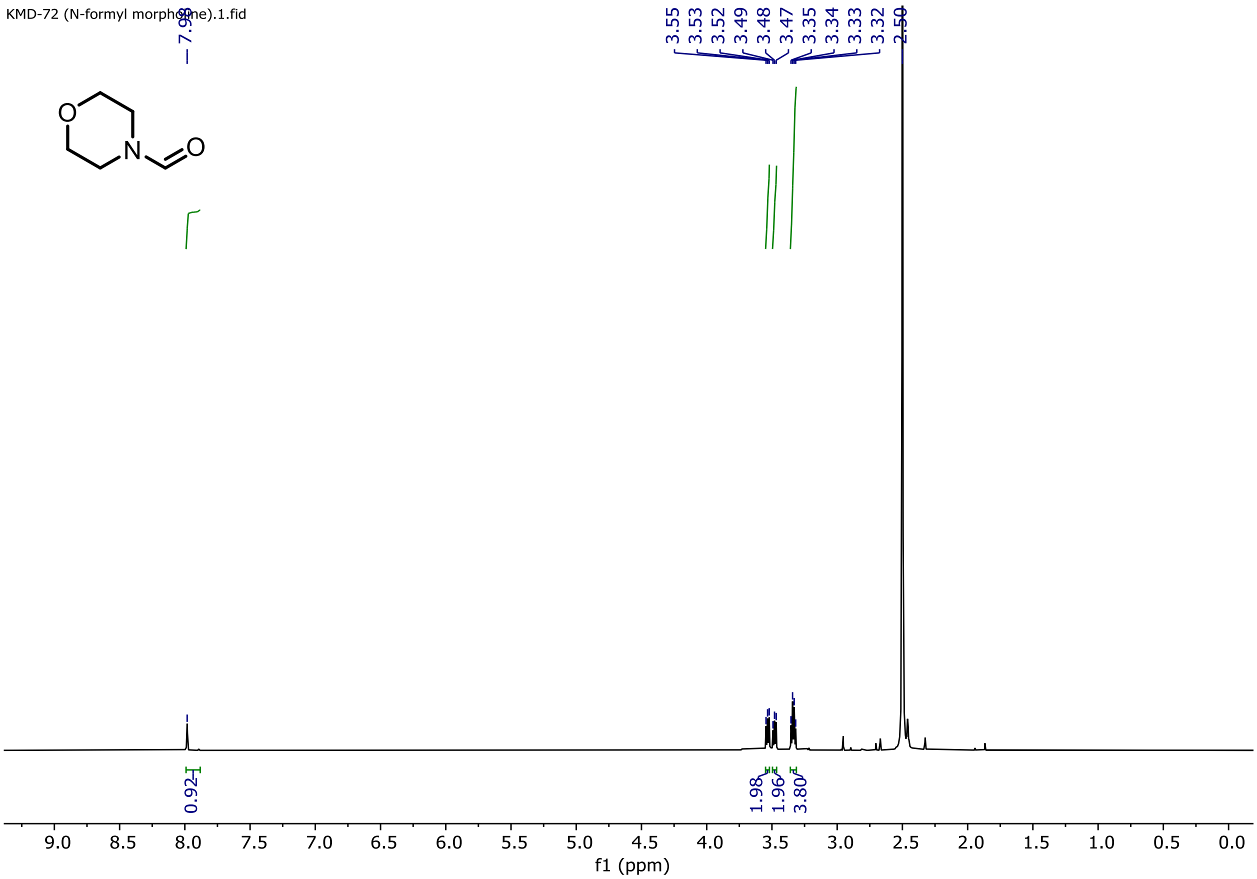

N-formylmorpholine:

^{1}H NMR (400 MHz, $CDCl_3$) δ: 8.02 (s, 1H), 3.66 – 3.62 (m, 4H), 3.56 – 3.53 (m, 2H), 3.39 – 3.36 (m, 2H) GC retention time 13.1 minutes to 13.2 minutes; EI-MS (m/z) calculated: 115, found 115.

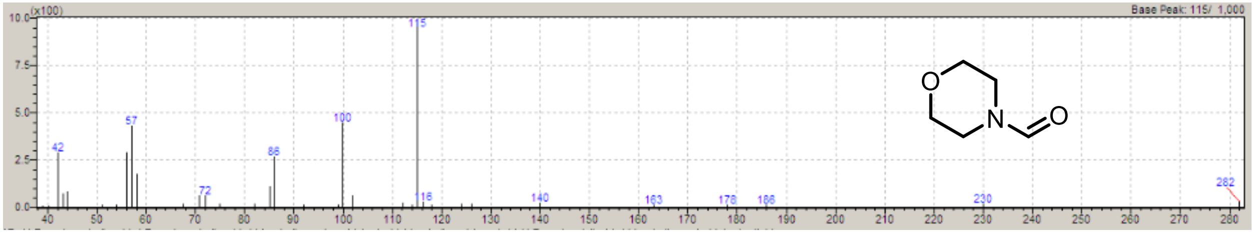

Figure S2: GC-MS analysis of a reaction mixture at the end of a catalytic test of synthesis of N-formylmorpholine.

2.3 Example of reproducibility between runs:

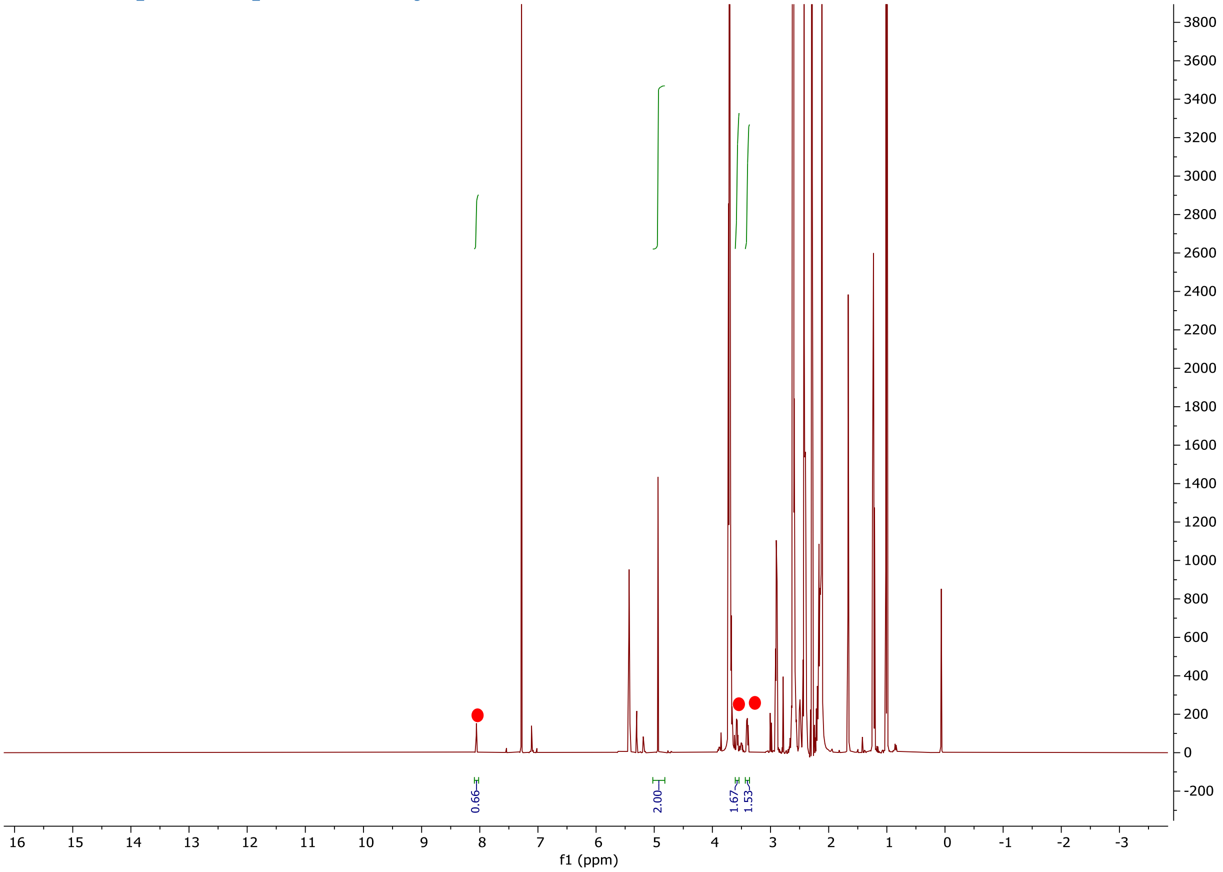

Figure S3a: ^{1}H NMR analysis of a reaction mixture at the end of a first run catalytic test of the transfer hydrogenation of CO_2 reductive coupling to morpholine at 130°C for 48hrs (table 1 entry 9).

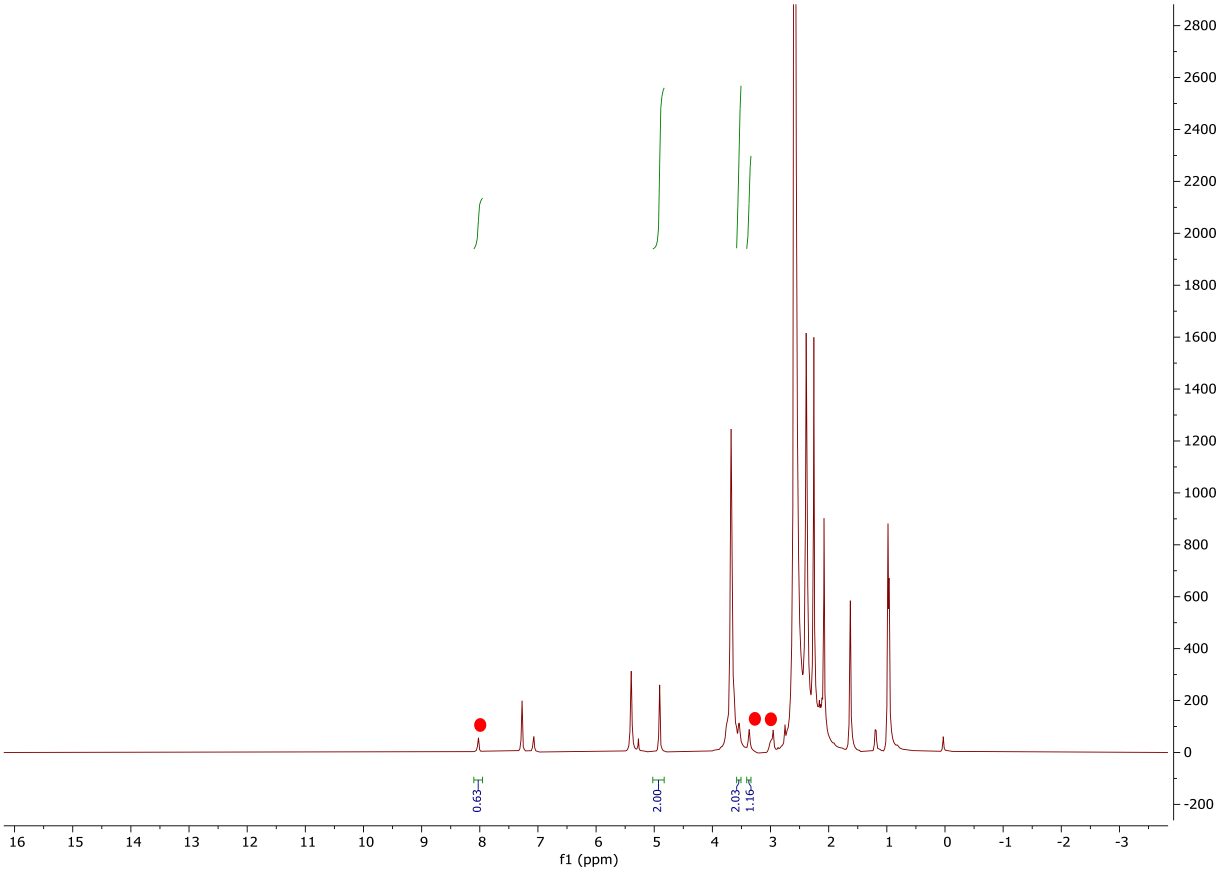

Figure S3b: ^{1}H NMR analysis of a reaction mixture at the end of a second run of a catalytic test of CO_2 reductive coupling to morpholine at 130°C for 48hrs (table 1 entry 9).

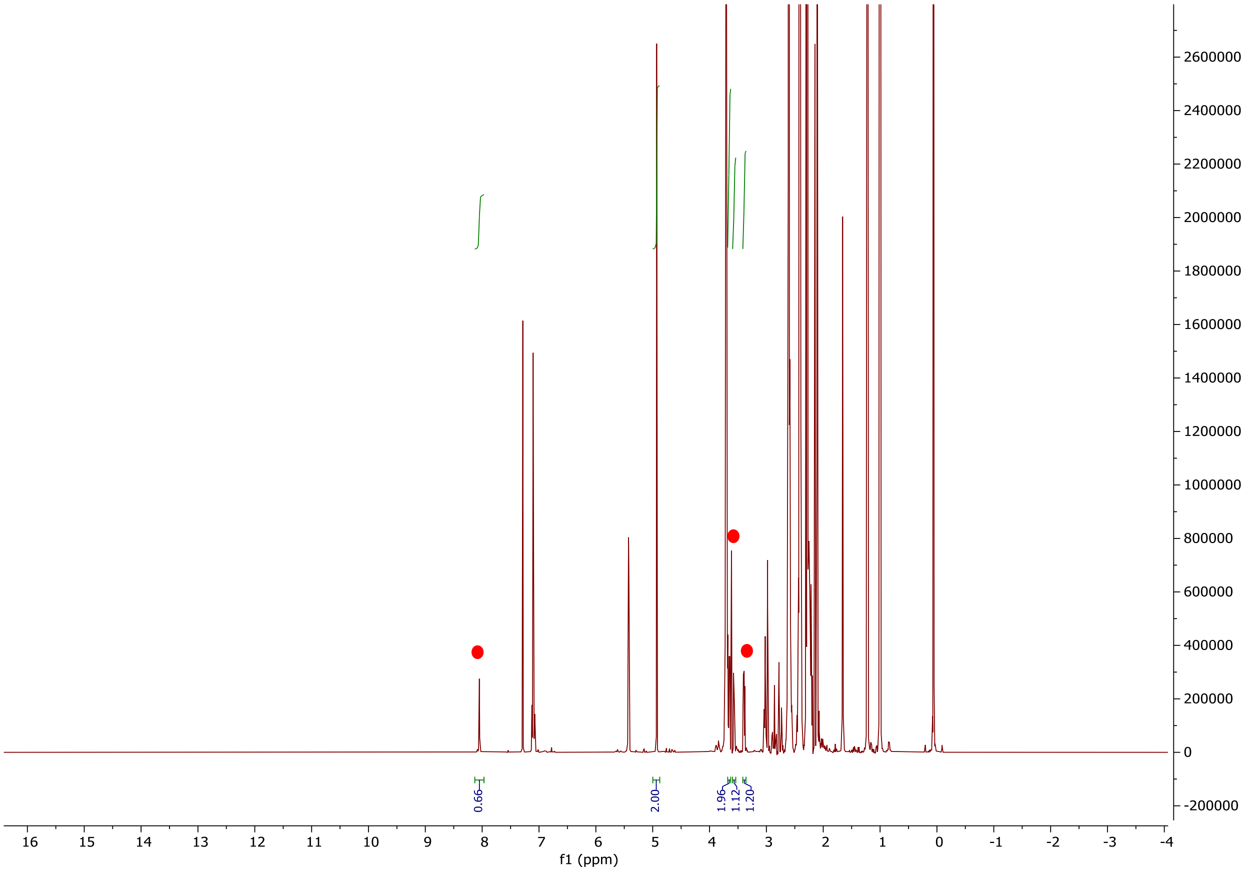

Figure S3c: ^{1}H NMR analysis of a reaction mixture at the end of a third run of a catalytic test of CO_2 reductive coupling to morpholine at 130°C for 48hrs (table 1 entry 9).

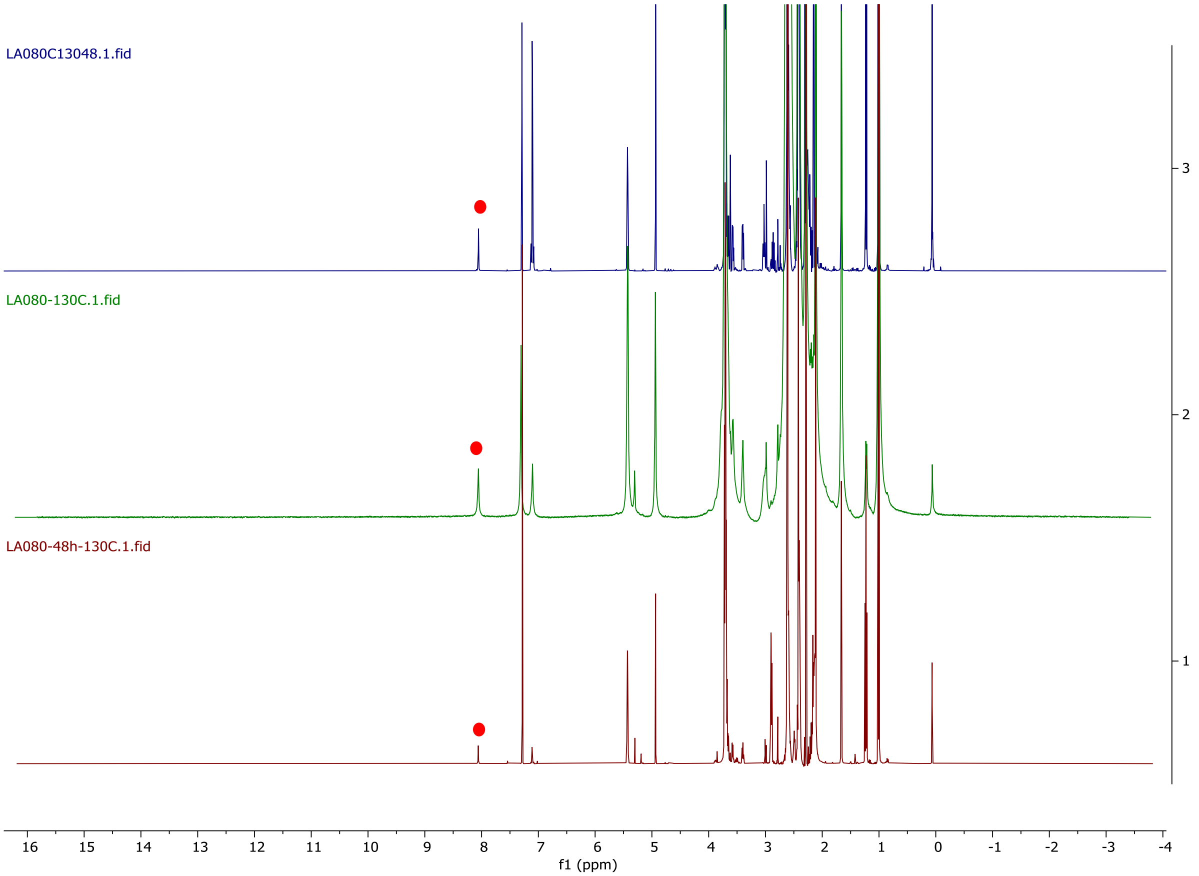

Figure S3d: Stacked of the above 3 spectra - ^{1}H NMR analysis of a reaction mixture at the end of a third run of a catalytic test of CO_2 reductive coupling to morpholine at 130°C for 48hrs

3. Solvent and temperature optimization

Table S1: Effect of solvent and of temperature on the $InOTf_3$ catalysed transfer hydrogenation of CO_2 and its reductive coupling to amines.

Entry	DMSO / NMM / γ-terp (mL)	Temperature (°C)	$InOTF_3$(mol%)	Yield (%)
**1**	2.0 / 1.0/ 0.5	90	10	15
**2**	2.0 / 1.0 / 1.0	90	10	19
**3**	2.0 / 1.0 / 1.5	90	10	17
4	2.5/ 0.5 / 1.0	90	10	12
**5**	1.5/ 1.5 / 1.0	90	10	11
**6**	2.0 / 1.0 / 1.0	110	10	29
**7**	2.0 / 1.0 / 1.0	130	5	35
**8**	2.0 / 1.0 / 1.0	150	10	49
**9[a]**	2.0 / 1.0 / 1.0	130	10	65
**10[a]**	2.0 / 1.0 / 1.0	150	10	91
**11**	2.0 / 1.0 / 1.0	130	0	0

Reaction conditions: Morpholine (1 mmol), LA = $InOTf_3$, CO_2 (4 bar), 22 hrs, average yield after three runs. Yields were determined by ^1H NMR with an internal standard. a) 48 hrs. NMM, γ-terpinene and DMSO were both used as received without further drying or purification.

4. 4. Substrate scope: N-formylation

4.1 Dimethyl formamide

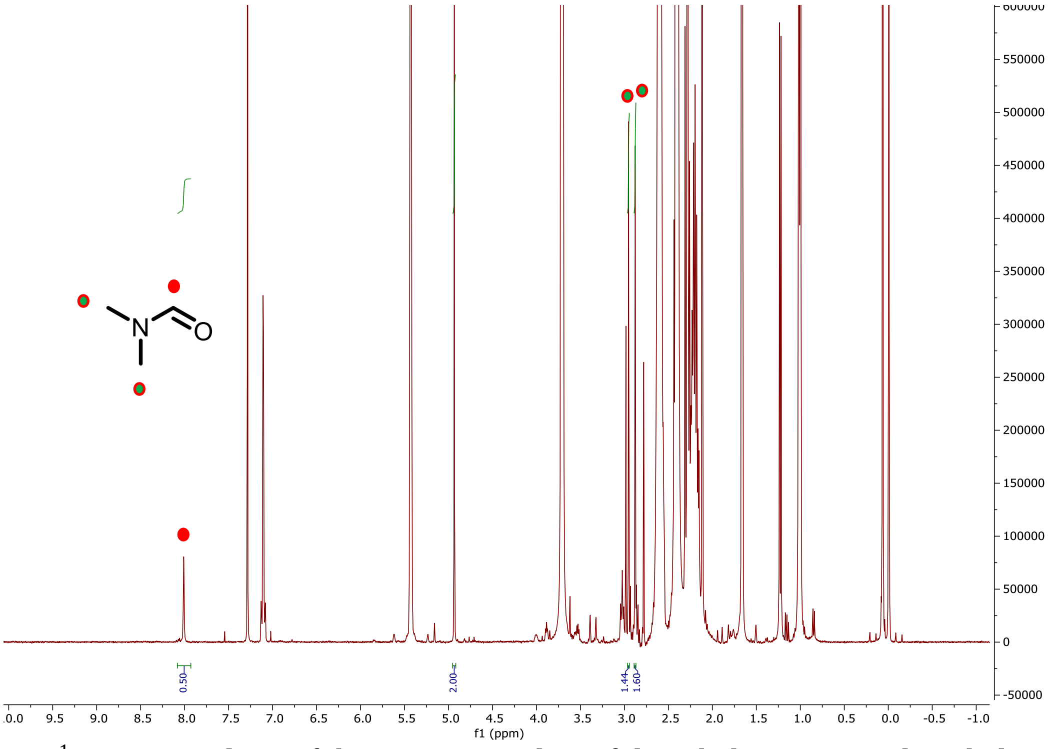

Figure S4: ^1H NMR analysis of the reaction product of dimethylammonium dimethyl carbamate.

^1H NMR (400 MHz, $CDCl_3$) δ: 8.00 (s, 1H), 2.95 (s, 3H), 2.87 (s, 3H) GC retention time 7.9 minutes to 8.1 minutes; EI-MS (m/z) calculated: 73, found 73

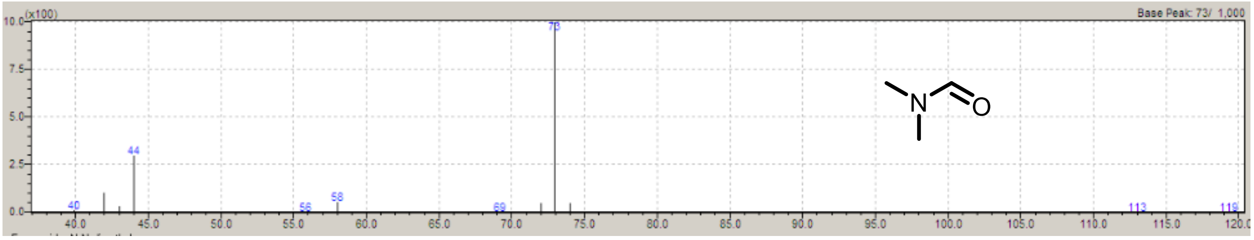

Figure S4b: EI-MS analysis of the reaction product of dimethylammonium dimethyl carbamate.

4.2 4-methylpiperazine-1-carbaldehyde

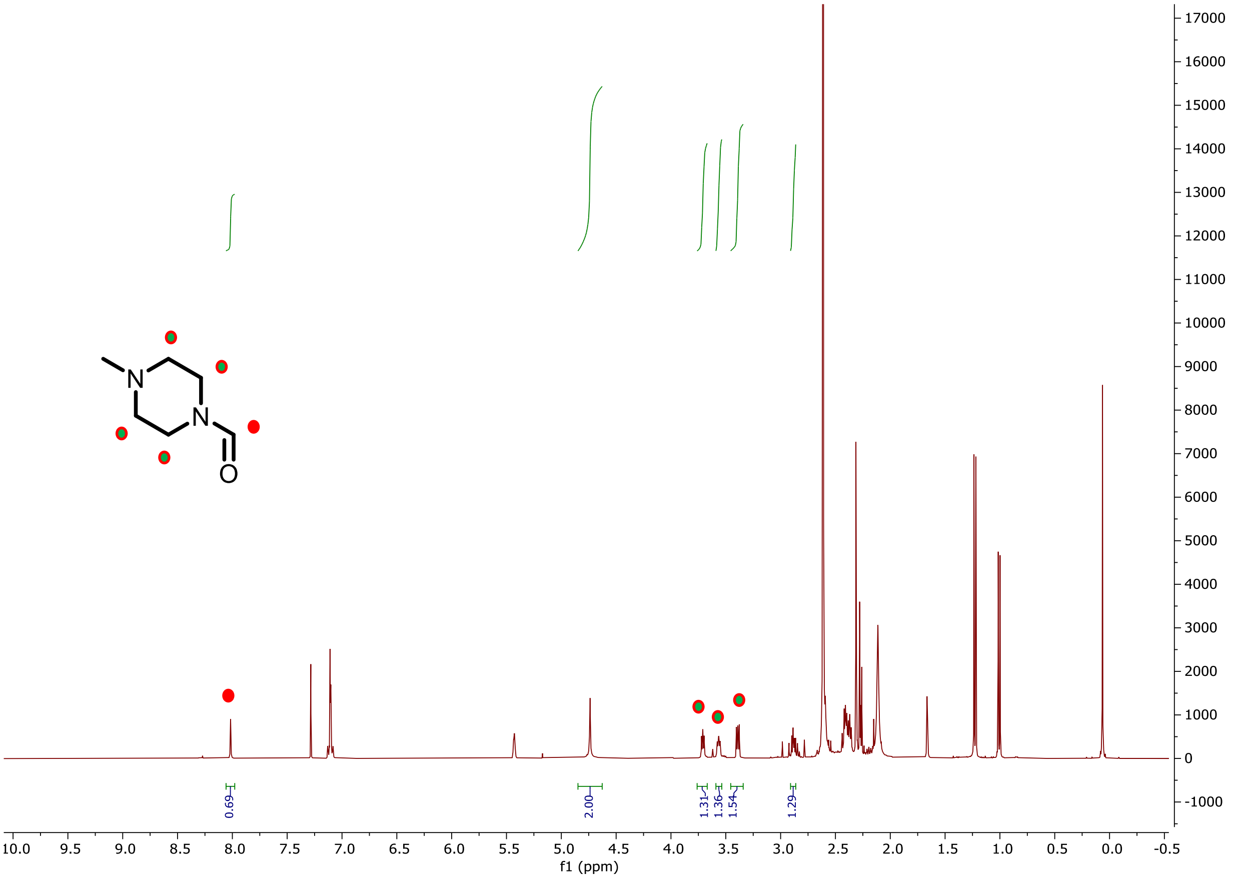

Figure S5: ^{1}H NMR analysis of the reaction product of N-methylpiperazine

^{1}H NMR (400 MHz, $CDCl_3$) δ: 8.01 (s, 1H), 3.73 – 3.68 (m, 2H), 3.59 – 3.54 (m, 2H), 3.41 – 3.37 (m, 2H), 2.90 – 2.86 (m, 2H). All other peaks were obscured by the reaction solvent. GC retention time 8.00 minutes to 8.2 minutes; EI-MS (m/z) calculated: 128, found 128

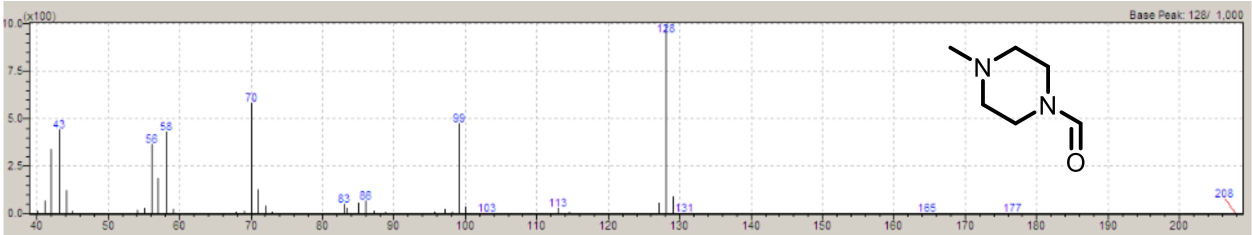

Figure S5b: EI-MS analysis of the reaction product of N-methylpiperazine

4.3 Pyrrolidine-1-carbaldehyde

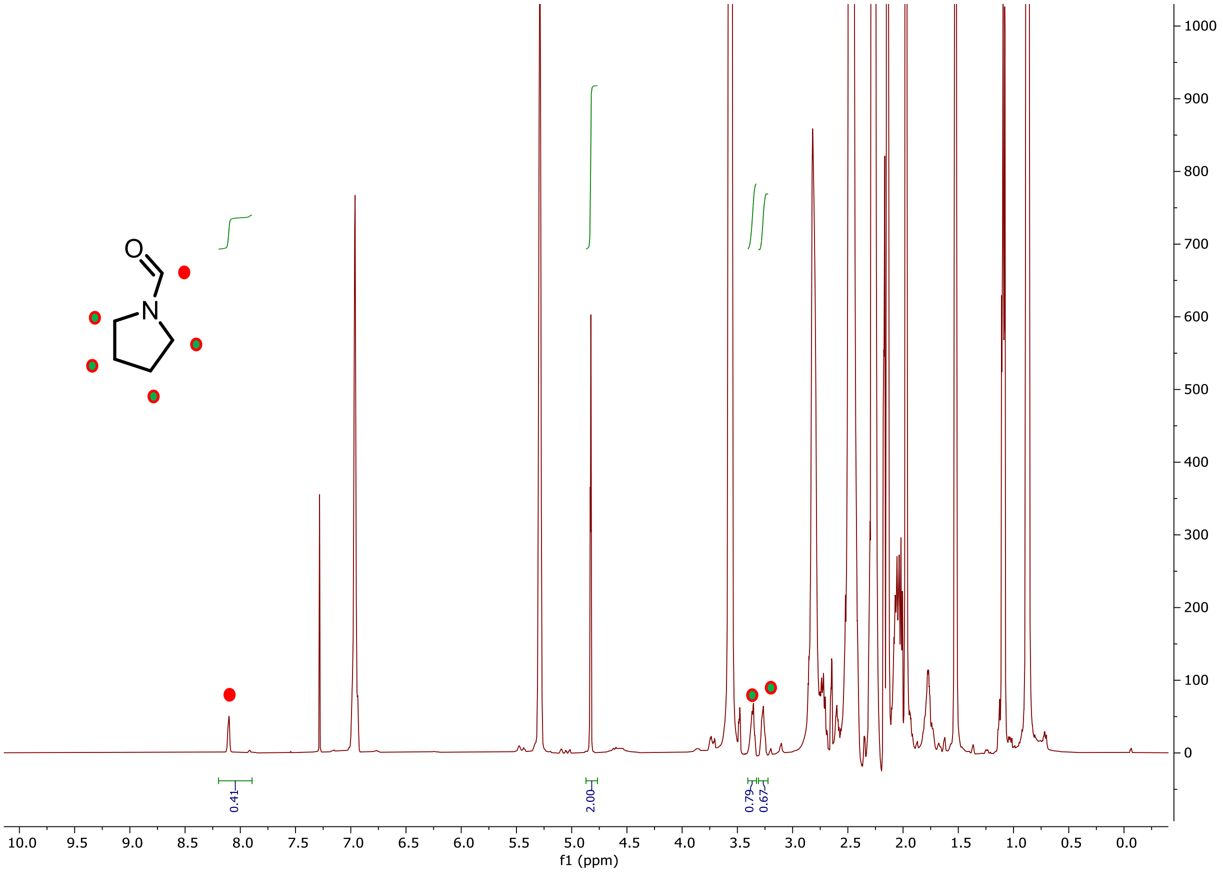

Figure S6: ^{1}H NMR analysis of the reaction product of pyrollidine

^{1}H NMR (400 MHz, $CDCl_3$) δ: 8.10 (s, 1H), 3.40 – 3.34 (m, 2H), 3.29 – 3.24 (m, 2H). All other peaks were obscured by the reaction solvent. GC retention time 11.9 minutes to 12.1 minutes; EI-MS (m/z) calculated: 99, found 99

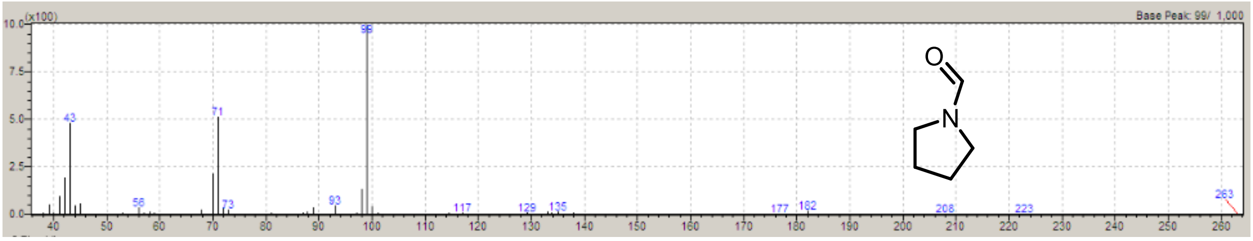

Figure S6b: ESI-MS analysis of the reaction product of pyrollidine.

4.4 4-(hydroxymethyl)piperidine-1-carbaldehyde

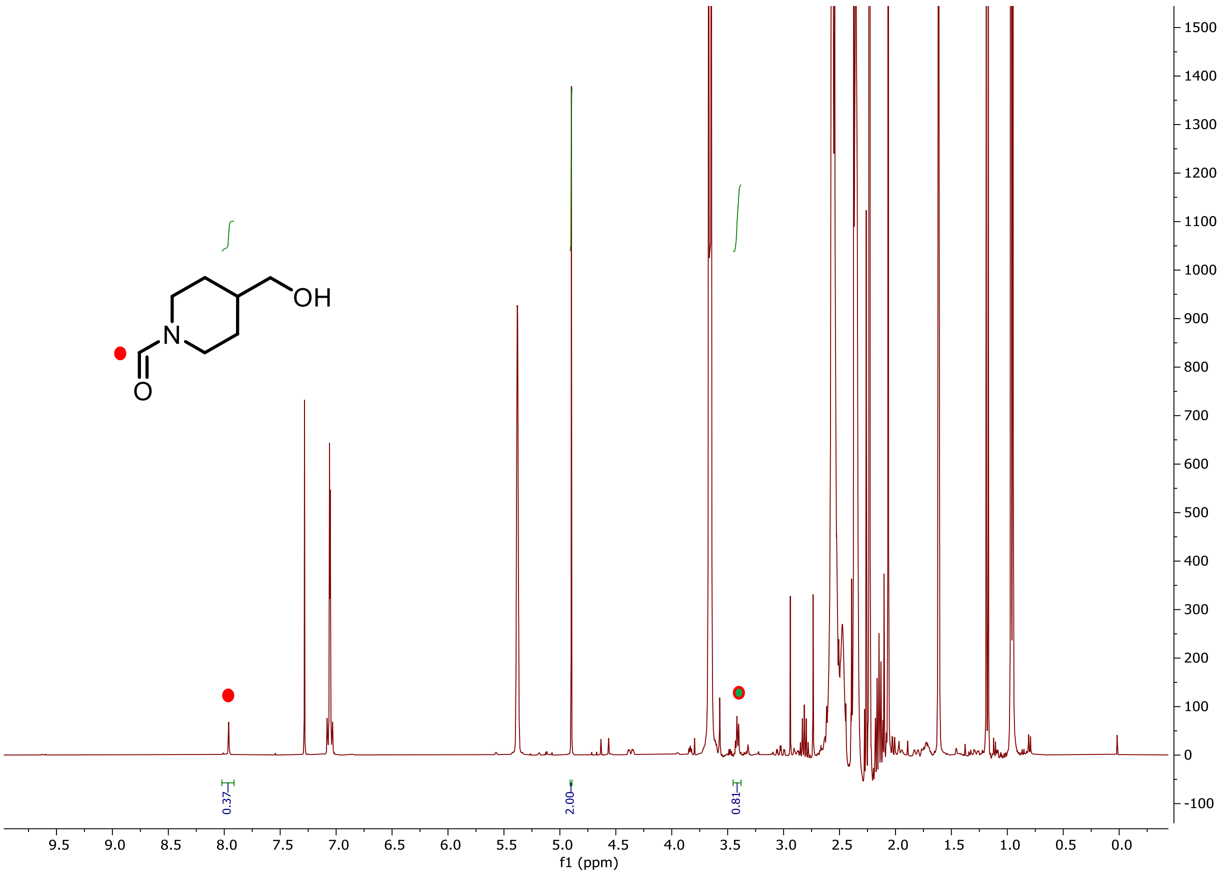

Figure S7: ^{1}H NMR analysis of the reaction product of piperidin-4-ylmethanol.

^{1}H NMR (400 MHz, $CDCl_3$) δ: 7.96 (s, 1H), 3.43 – 3.39 (m, 2H). All other peaks were obscured by the reaction solvent. GC retention time 21.1 minutes to 21.4 minutes; EI-MS (m/z) calculated: 143, found 143

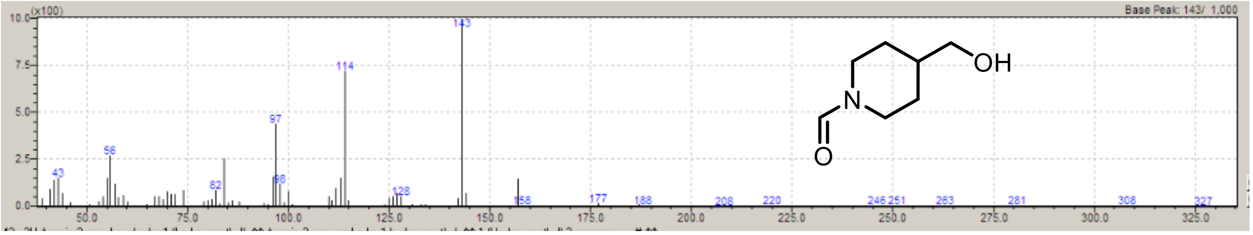

Figure S7b: EI-MS analysis of the reaction product of Piperidin-4-ylmethanol.

4.5 N-allyl-N-methylformamide

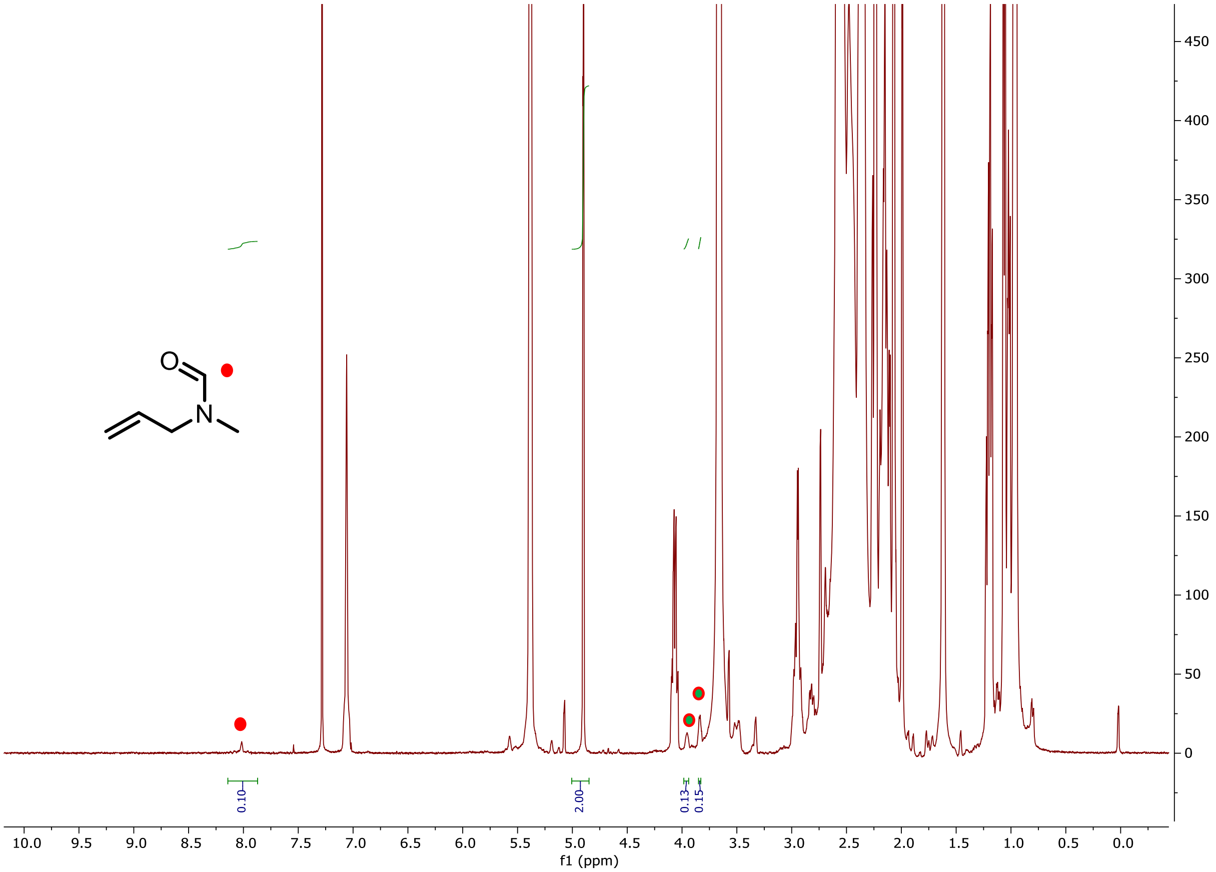

Figure S8: ^{1}H NMR analysis of the reaction product of N-allylmethylamine.

^{1}H NMR (400 MHz, $CDCl_3$) δ: 8.02 (s, 1H), 3.97 -3.93 (m, 1H), 3.86 – 3.82 (m, 1H). All other peaks were obscured by the reaction solvent. GC retention time minutes 9.5 to 9.7minutes; EI-MS (m/z) calculated: 99, found 99

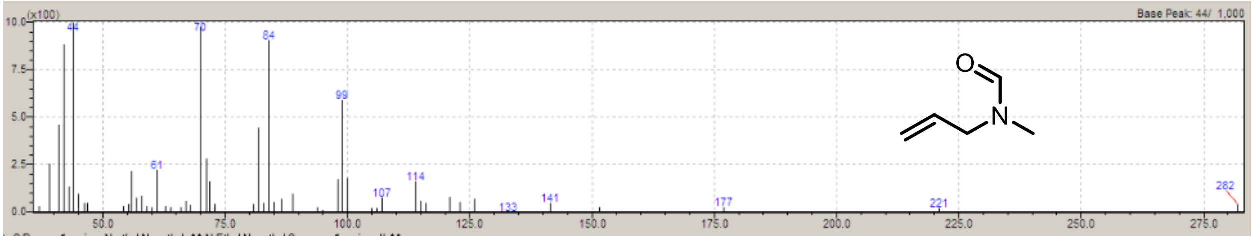

Figure S8b: EI-MS analysis of the reaction product of N-allylmethylamine.

4.6 N-cyclohexylformamide

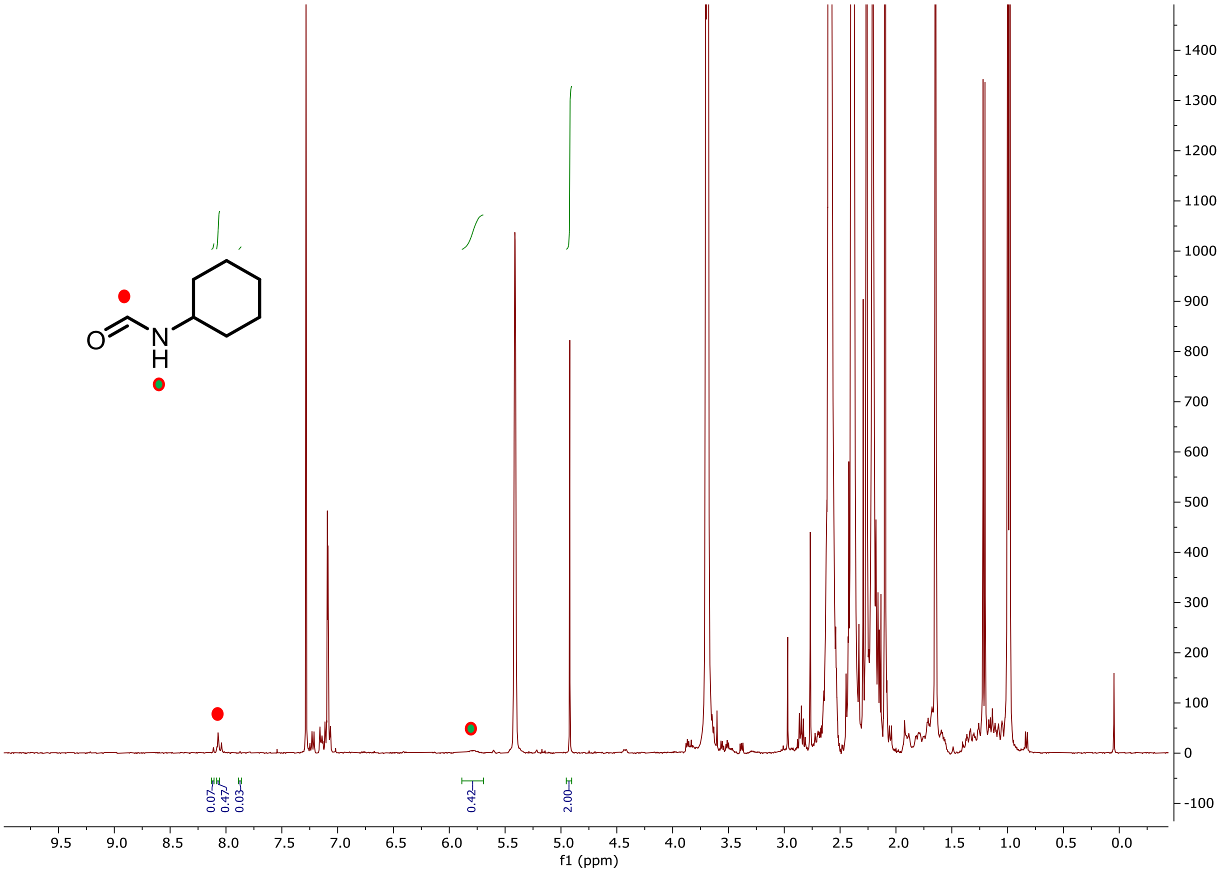

Figure S9: ^{1}H NMR analysis of the reaction product of cyclohexylamine

^{1}H NMR (400 MHz, $CDCl_3$) δ: 8.07 (s, 1H), 5.80 (br s, 1H). All other peaks were obscured by the reaction solvent. GC retention time 14.8 minutes to 15.1 minutes; EI-MS (m/z) calculated: 127, found 127

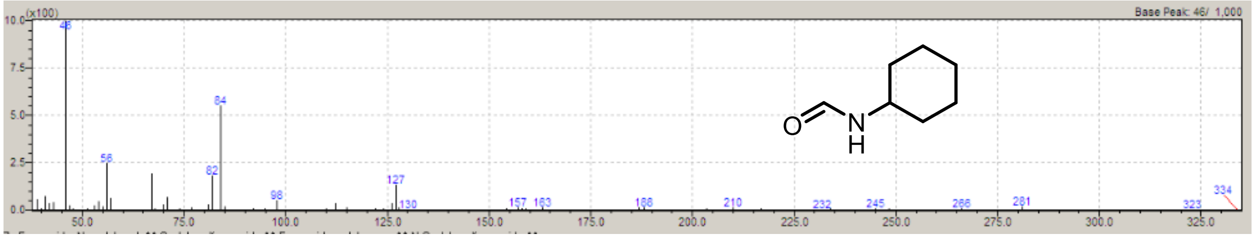

Figure S9b: EI-MS analysis of the reaction product of cyclohexylamine.

4.7 4-acetylpiperazine-1-carbaldehyde

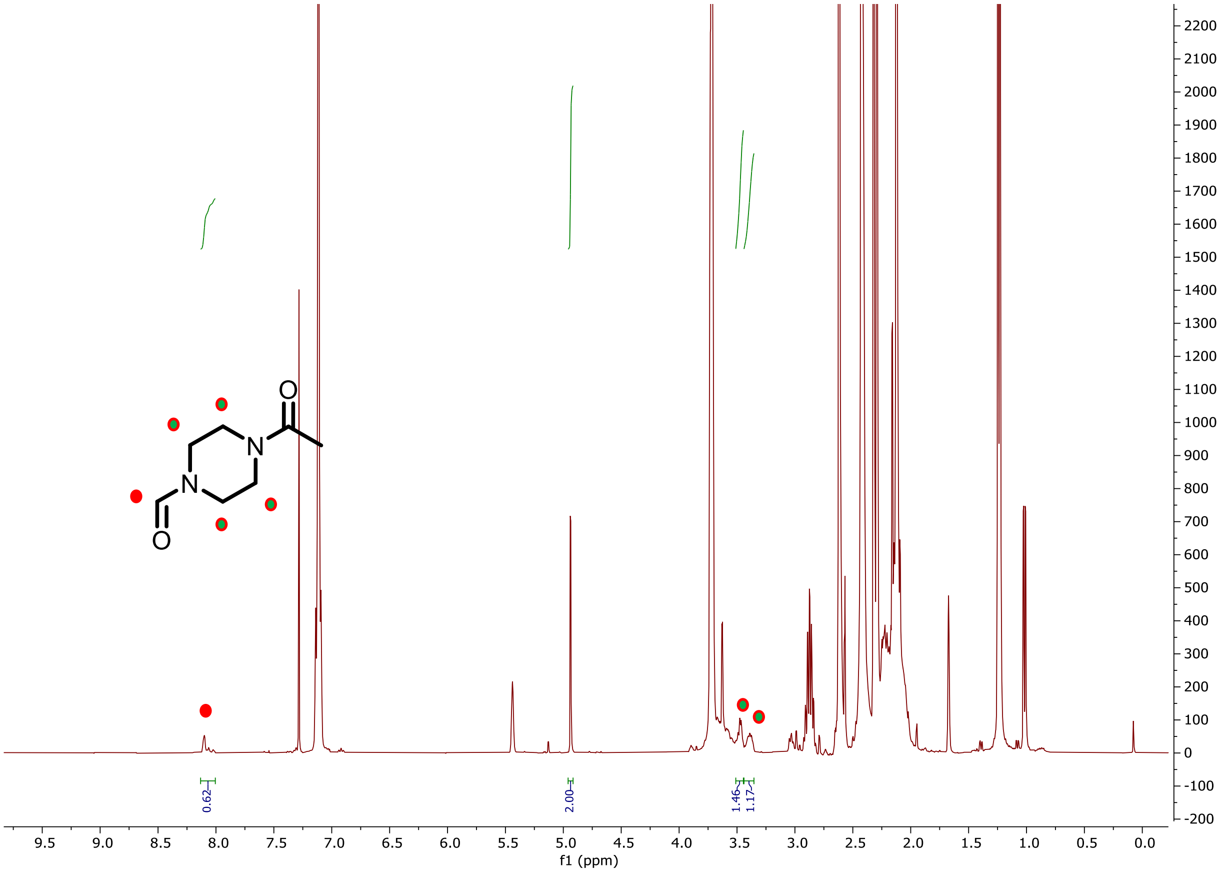

Figure S10: ^{1}H NMR analysis of the reaction product of 4-acetylpiperizine

^{1}H NMR (400 MHz, $CDCl_3$) δ: 8.09 (s, 1H), 3.50 – 3.44 (m, 2H), 3.42 – 3.36 (m, 2H). All other peaks were obscured by the reaction solvent. GC retention time 25.2 minutes to 25.5 minutes; EI-MS (m z) calculated:156, found 156

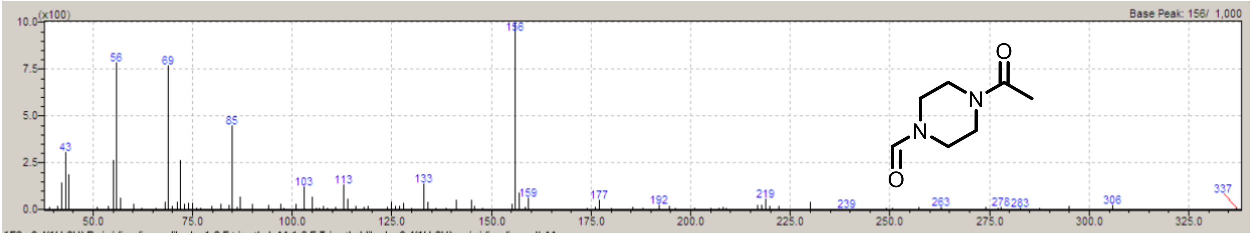

Figure S10b: EI-MS analysis of the reaction product of 4-acetylpiperizine.

4.8 n-Hexylformamide

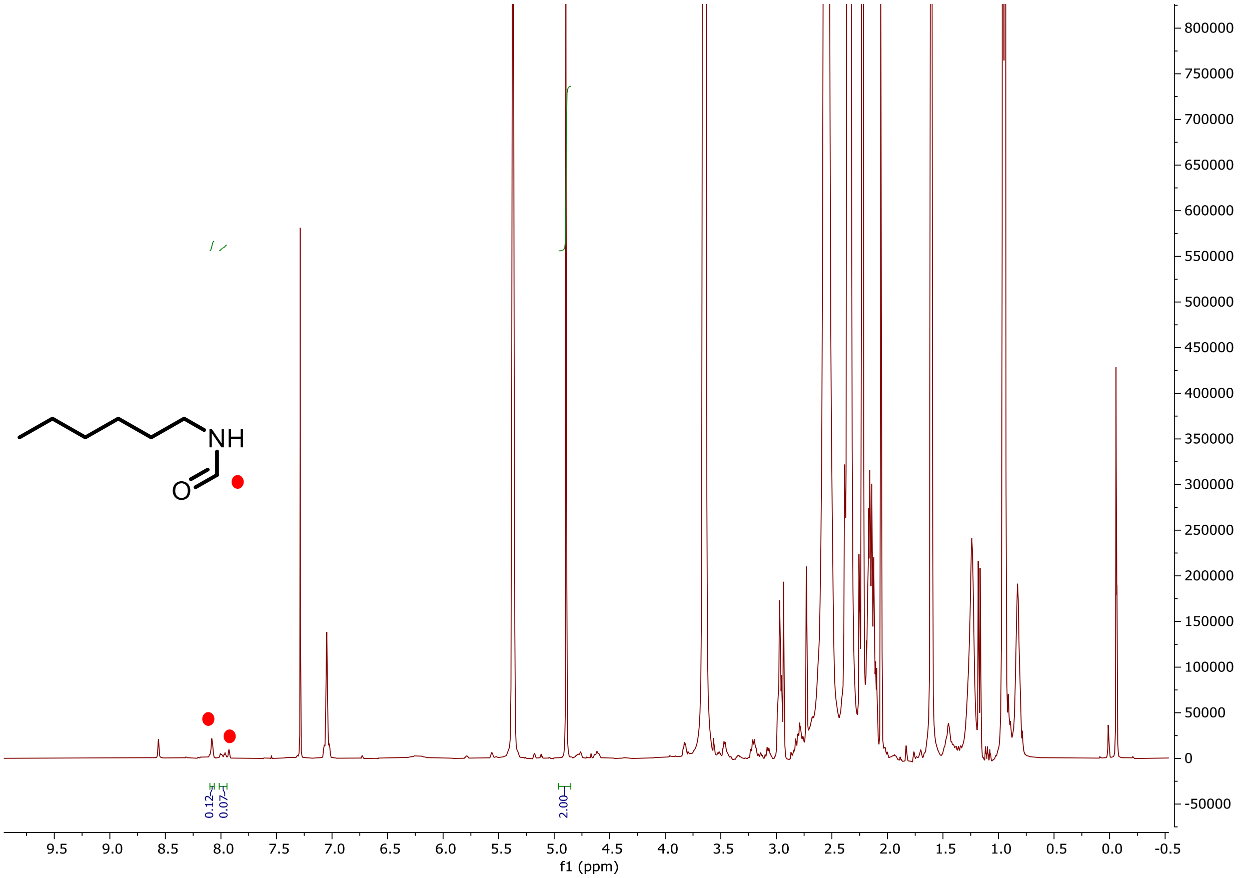

Figure S11: ^{1}H NMR analysis of the reaction product of n-hexylamine.

^{1}H NMR (400 MHz, $CDCl_3$) δ: 8.09 (s, 0.8H), 7.98 (d, 0.2H) All other peaks were obscured by the reaction solvent. GC retention time 14.0 minutes to 14.3 minutes; EI-MS (m/z) calculated:, 129 found 129

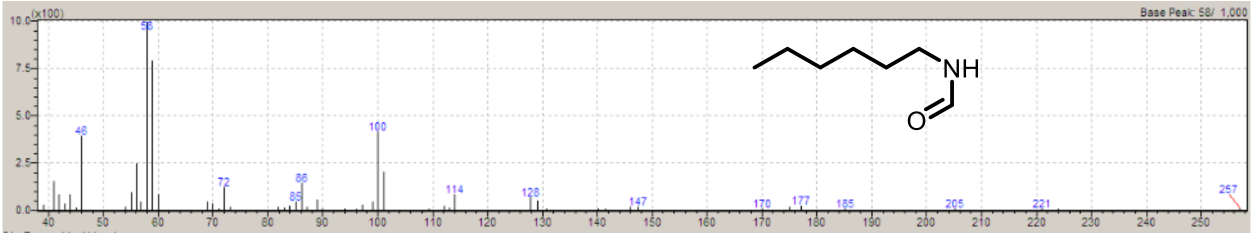

Figure S11b: EI-MS analysis of the reaction product of n-hexylamine.

4.9 N-benzylformamide

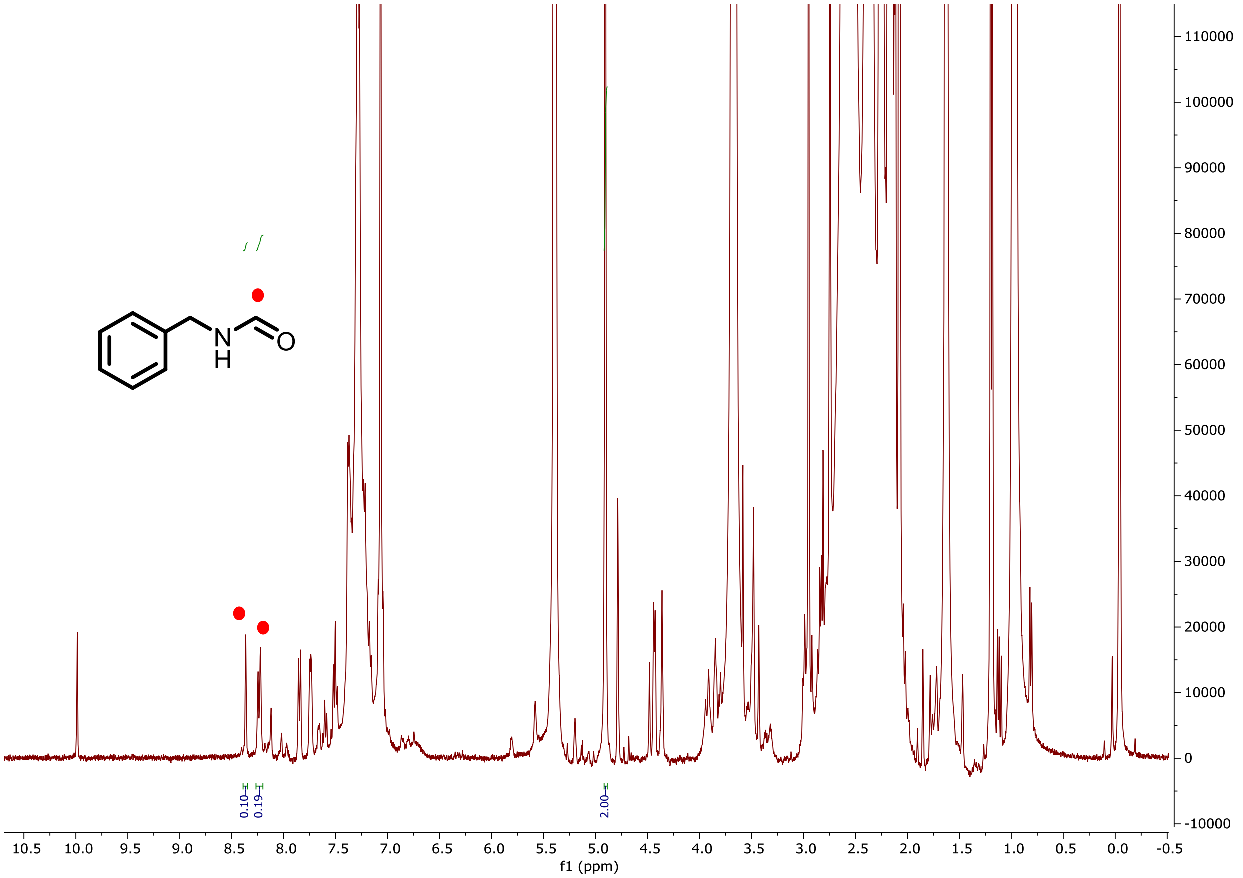

Figure S12: ^{1}H NMR analysis of the reaction product of benzylamine.

^{1}H NMR (400 MHz, $CDCl_3$) δ: 8.36 (s, 0.8H), 8.24 (d, 0.2H). All other peaks were obscured by the reaction solvent. GC retention time 7.8 to 8.0 minutes; EI-MS (m/z) calculated:135, found 135

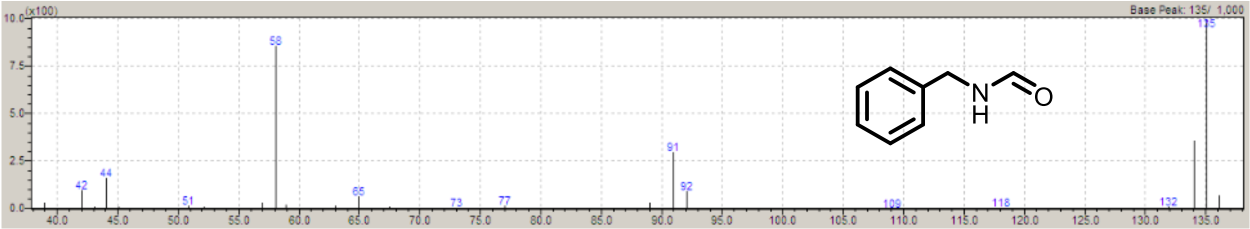

Figure S12b: EI-MS analysis of the reaction product of Benzylamine.

4 Synthesis of azoles

4.10 Benzimidazole

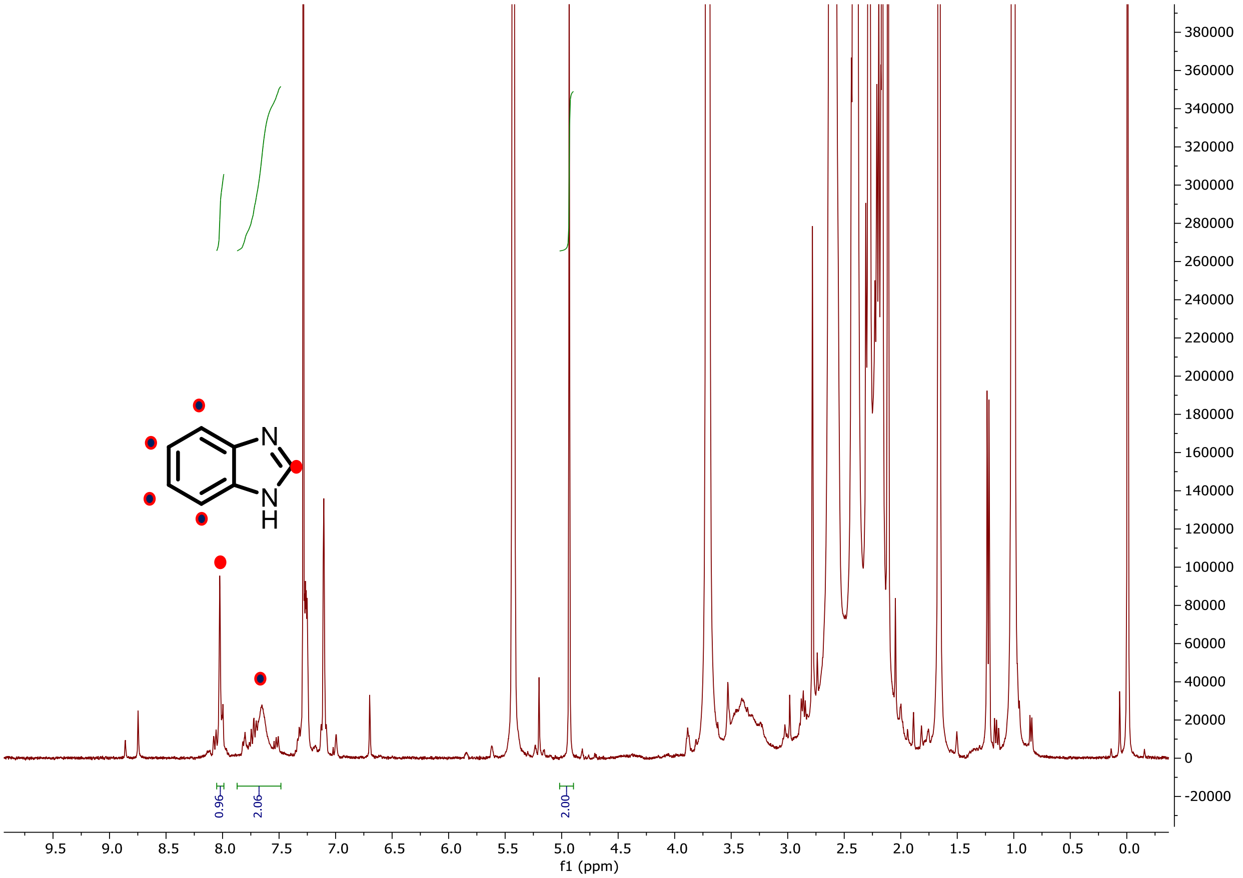

Figure S13: ^{1}H NMR analysis of the reaction product of *o*-phenylenediamine.

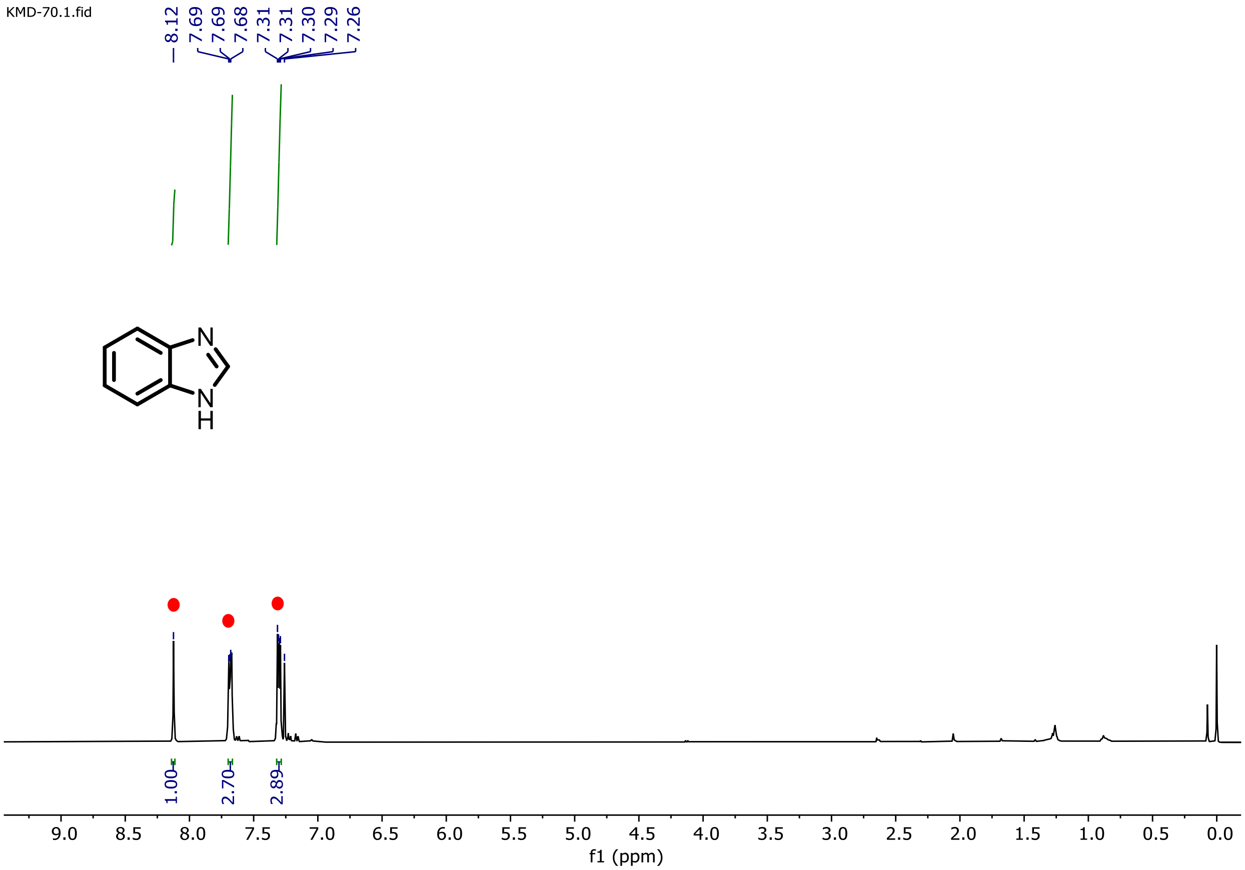

Figure S13b: ^{1}H NMR of benzimidazole isolated from the cyclization reaction of o-phenylenediamine

^{1}H NMR (400 MHz, $CDCl_3$) δ: 8.12 (s, 1H) 7.69 – 7. 68 (m, 2H), 7.31 – 7.29 (m, 2H). GC retention time 28.2 minutes to 28.7 minutes; EI-MS (m/z) calculated: 118, found 118 all other peaks were obscured due to NMR solvent

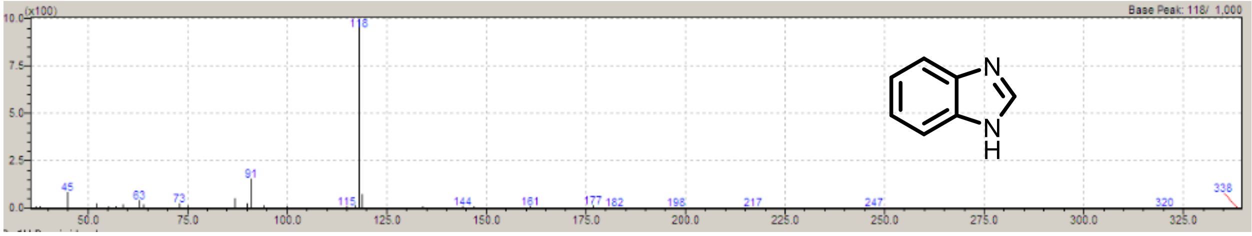

Figure S13c: EI-MS analysis of the reaction product of *o*-phenylenediamine.

4.11 Benzothiazole

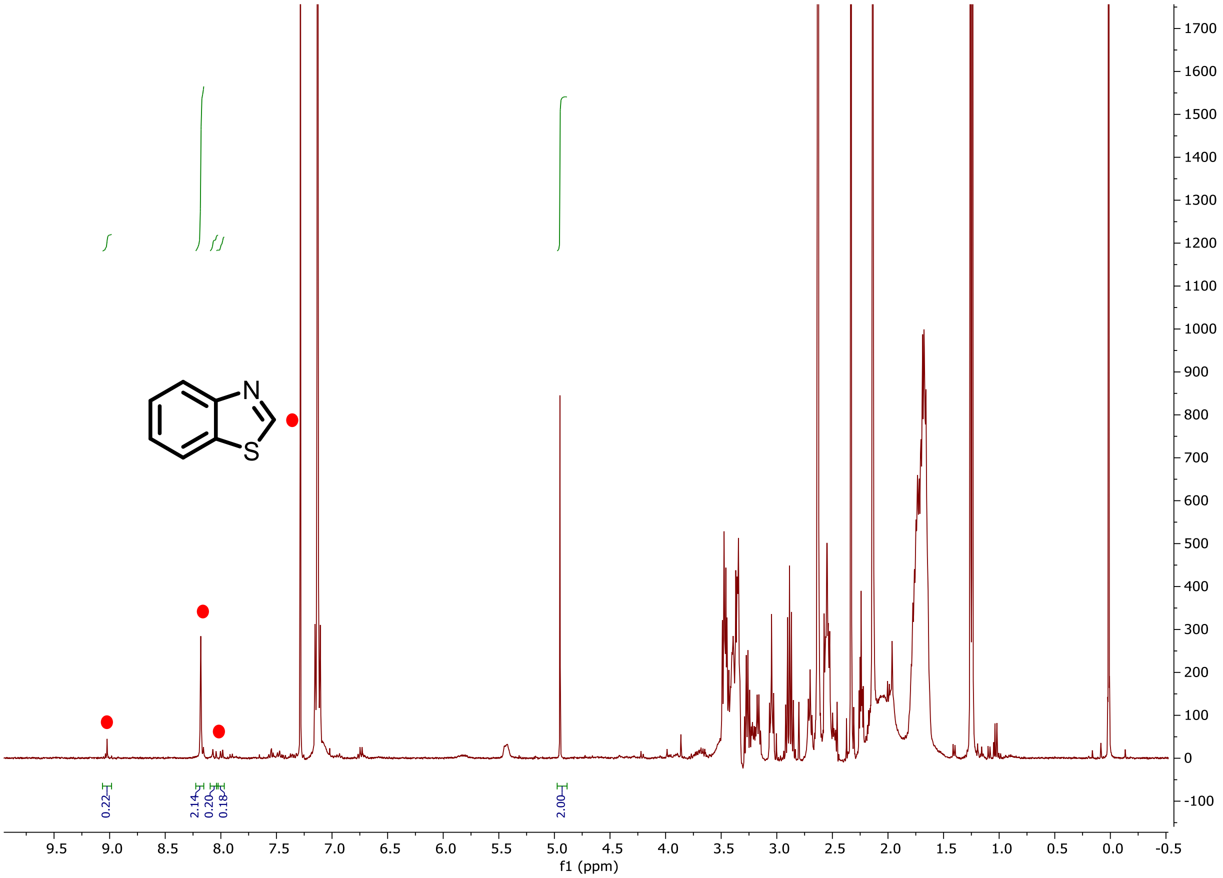

Figure S14: ^{1}H NMR analysis of the reaction product of 2-aminobenzenethiol.

^{1}H NMR (400 MHz, $CDCl_3$) δ: 9.01 (s, 1H), 8.21 – 8.15 (m, 2H), 8.06 – 7.99 (m, 2H).
GC retention time 13.4 minutes to 13.7 minutes; EI-MS (m/z) calculated: 135, found 135 all other peaks were obscured due to NMR solvent. Presence of DBU formate in the NMR (peak integrating to 2.14 (42.8 turnovers of DBU formate). DBU was used as base as proved more efficient than NMM for the above substrate.

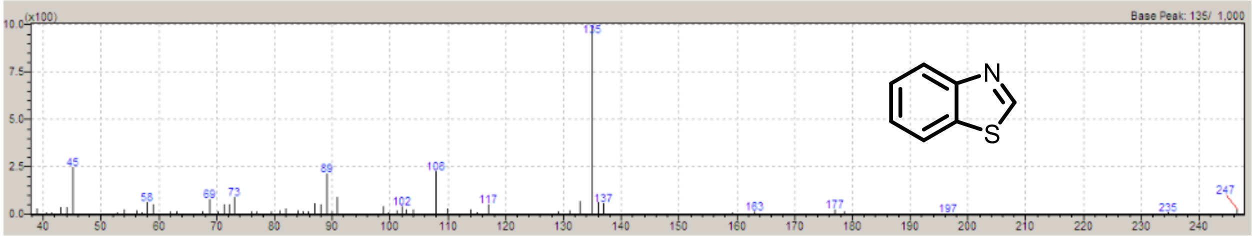

Figure S14b: EI-MS analysis of the reaction product of 2-aminobenzenethiol.

4.12 N-phenylbenzimidazole

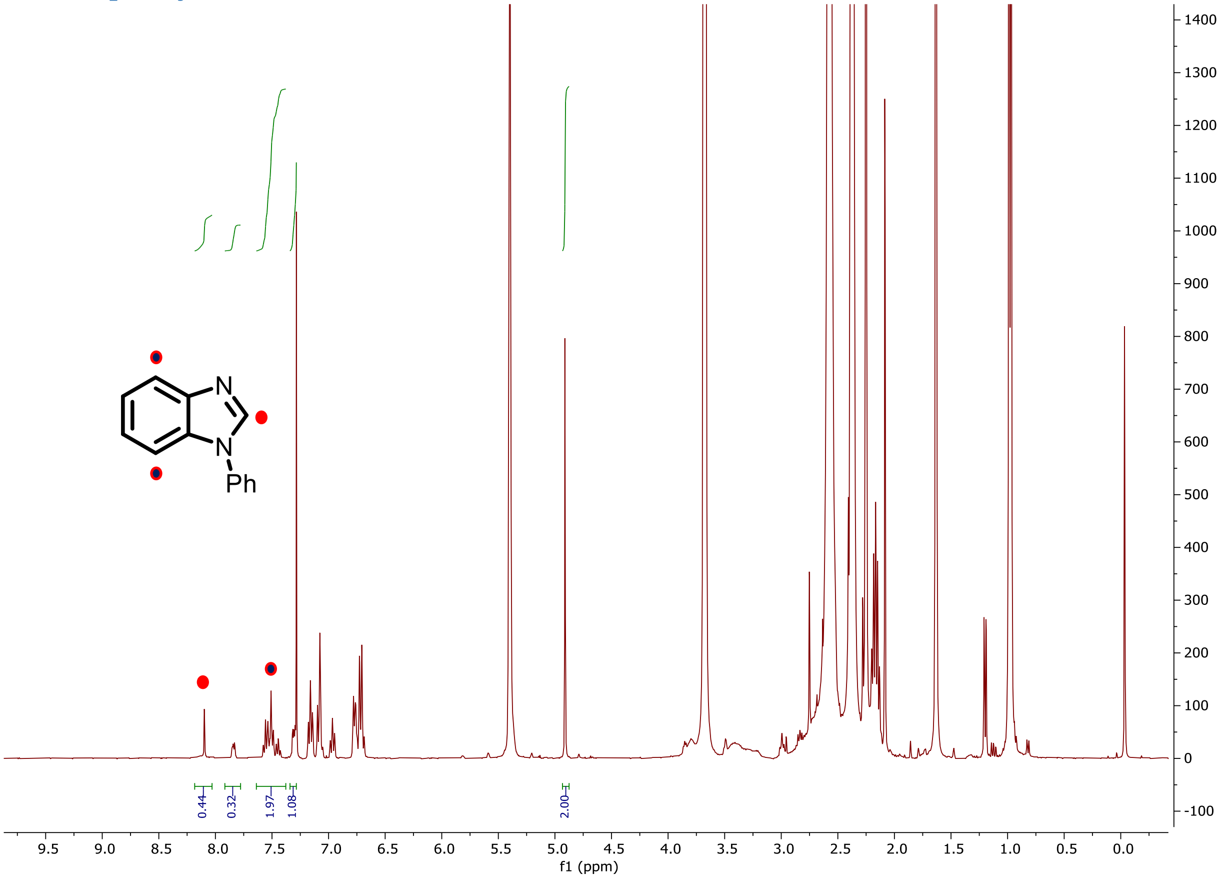

Figure S15: ^{1}H NMR analysis of the reaction product of 2-aminodiphenylamine.

^{1}H NMR (400 MHz, $CDCl_3$) δ:8.10 (s, 1H), 7.87 – 7.81 (m, 1H), 7.56 – 7.40 (m, 6H), 7.36 – 7.27 (m, 2H) GC retention time 27.8 minutes to 28.1 minutes; EI-MS (m/z) calculated: 194, found 194 all other peaks were obscured due to NMR solvent

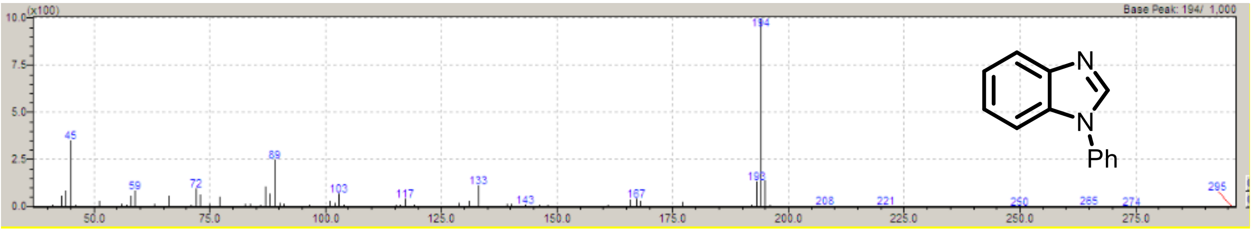

Figure S15b: EI-MS analysis of the reaction product of 2-aminodiphenylamine.

4.13 4-methylbenzimidazole

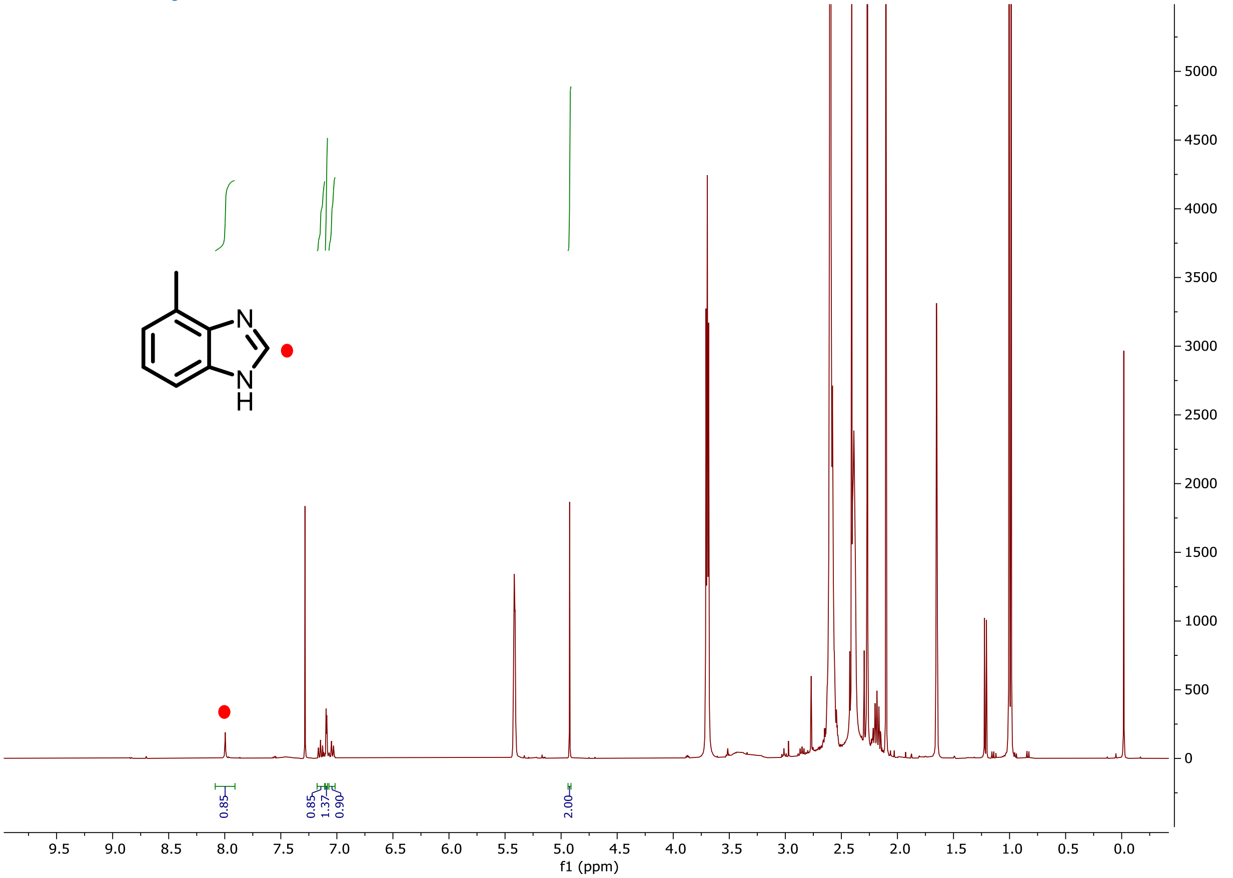

Figure S16: ^{1}H NMR analysis of the reaction product of 3-methyl-1,2-benzenediamine.

^{1}H NMR (400 MHz, $CDCl_3$) δ: 8.00 (s, 1H), 7.17 -7.12 (m, 1H), 7.10 – 7.08 (m, 1H), 7.07 – 7.02 (m, 1H) All other peaks were obscured by the reaction solvent. GC retention time 28.4 minutes to 28.8 minutes; EI-MS (m/z) calculated: 132, found 132

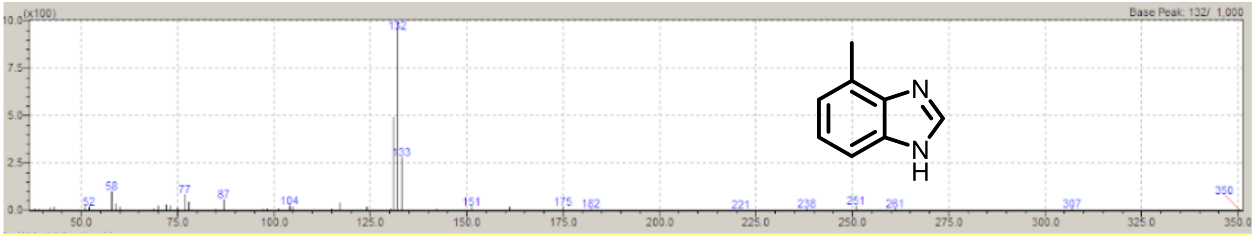

Figure S16b: EI-MS analysis of the reaction product of 3-methyl-1,2-benzenediamine.

5. Direct formylation of morpholine using DBU formate and N-methyl morpholinium formate.

5.1 General Procedure:

In an NMR tube, Lewis base (1 mmol of either N-methylmorpholine or DBU) and formic acid (1mmol) were dissolved in 1 mL of DMSO. The NMR tube was shaken vigorously and allowed to sit for 10 minutes. After which morpholine (1 mmol) was added and the NMR tube was heated to 130 ºC for 24 hrs after which the sample was measured by NMR. The amount of formate to formylamine conversion was measured by the ratio between the formate peak to formylamine peaks in the ^{1}H NMR.

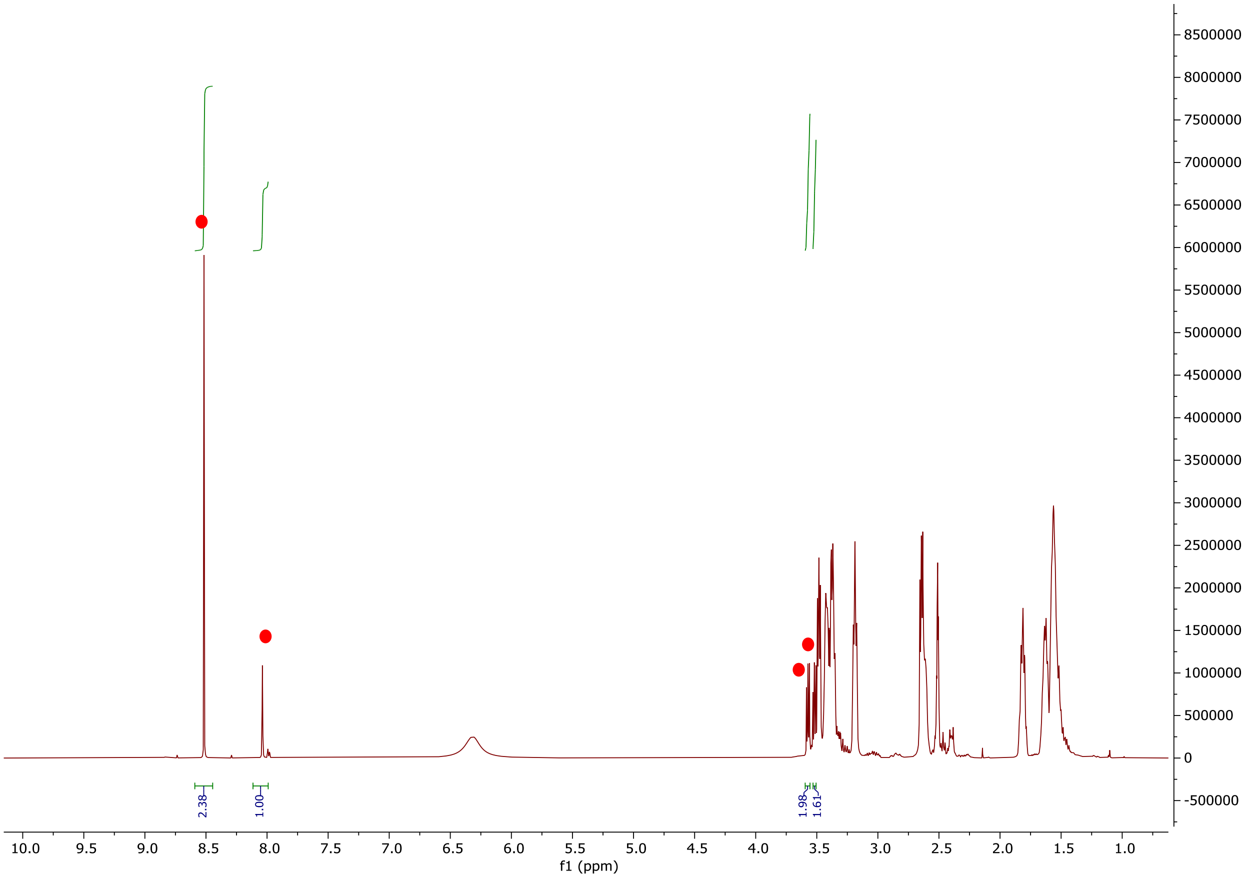

Figure S17: Result of the direct reaction between DBU formate and morpholine.

^{1}H NMR (400 MHz, $CDCl_3$) δ: 8.53 (s, 1H (DBU formate)), 8.02 (s, 1H (N-formylmorpholine), 3.57 (m, 2H), 3.52 (m, 2H) all other peaks of N-formylmorpholine are covered by DBU formate

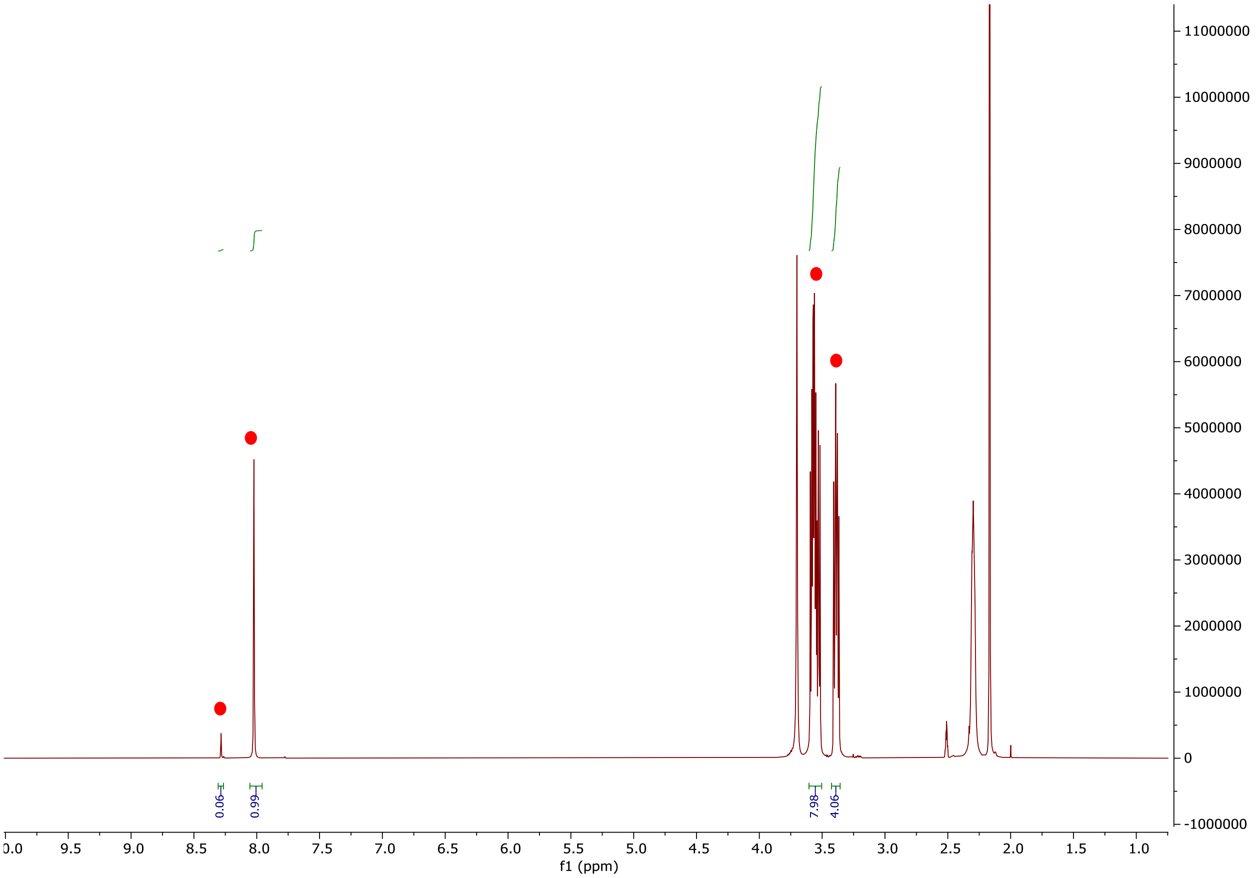

Figure S18: Result of the direct reaction between NMM formate and morpholine.

^{1}H NMR (400 MHz, $CDCl_3$) δ: 8.27 (s, 1H (NMM formate)), 8.02 (s, 1H (N-formylmorpholine), 3.59- 3.54(m, 4H), 3.54- 3.51 (m, 2H) all other peaks of N-formylmorpholine are covered by NMM and NMM formate

6. Synthesis of [DBUH][formate]

6.1 General Procedure for the synthesis of [DBUH][formate]:

In air $In(OTf)_3$ (0.05 mmol) was dissolved in the solvent mixture (4 mL; 1:1:2 mixture of DBU: γ-terpinene: DMSO) in a stainless-steel autoclave. The autoclave was then sealed and purged 5 times with the desired pressure of CO_2. The temperature and stirring rate were set using the Specview program on Parr 5000 series multi reactor system. T = 0 was defined as the time the heating starts. The heating was turned off at T = end of the stated reaction time and immediately cooled down i.e., for a reaction time of 24 hours the heating was turned off after 24 hours, removed from the heating mantel and cooled immediately. After which, dibromomethane (1 mmol) was added to the reactor, stirred and an aliquot was taken for ^{1}H NMR analysis in DMSO-d_6. The [DBU][formate] yield was quantified by ^{1}H NMR analysis with the added dibromomethane as the internal standard.

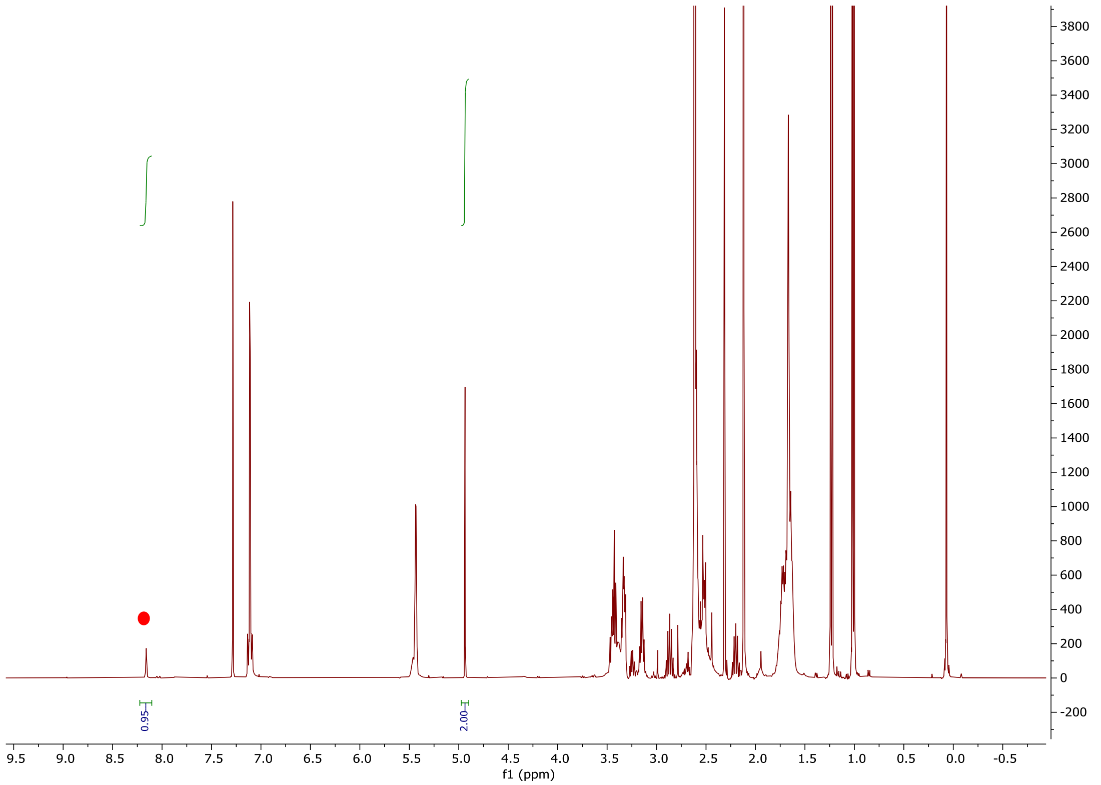

Figure S19: Result of the formation of DBU formate from CO_2.

^{1}H NMR (400 MHz, $CDCl_3$) δ: 8.16 (s, 1H). Peaks corresponding to the formate salt are obscured by free DBU.

6.2: Effect of CO_2 pressure on synthesis of DBU formate

**Entry**	**LA**	**LB**	**Temp (°C)**	**CO_2 Pressure (bar)**	**Time (hours)**	**Mmol of formate produced**
**1**	$InOTf_3$	DBU	130	4	48	0.95
**2**	$InOTf_3$	DBU	130	6	48	1.94
**3**	$InOTf_3$	DBU	130	10	48	0.88
**4**	$InOTf_3$	DBU	130	4	72	1.45

7. Formic acid decomposition.

7.1 General Procedure:

In an NMR tube $In(OTF)_3$ (0.05mmol), NMM (200 μL) and formic acid (1mmol) were dissolved in 1 mL of DMSO-*d6*. The NMR tube was heated to 130 ºC for 24 hrs after which the sample was measured by NMR. The yield of H_2 gas was 47% and was calculated by the ratio between the peak of H_2 and the formate peak of NMM formate.

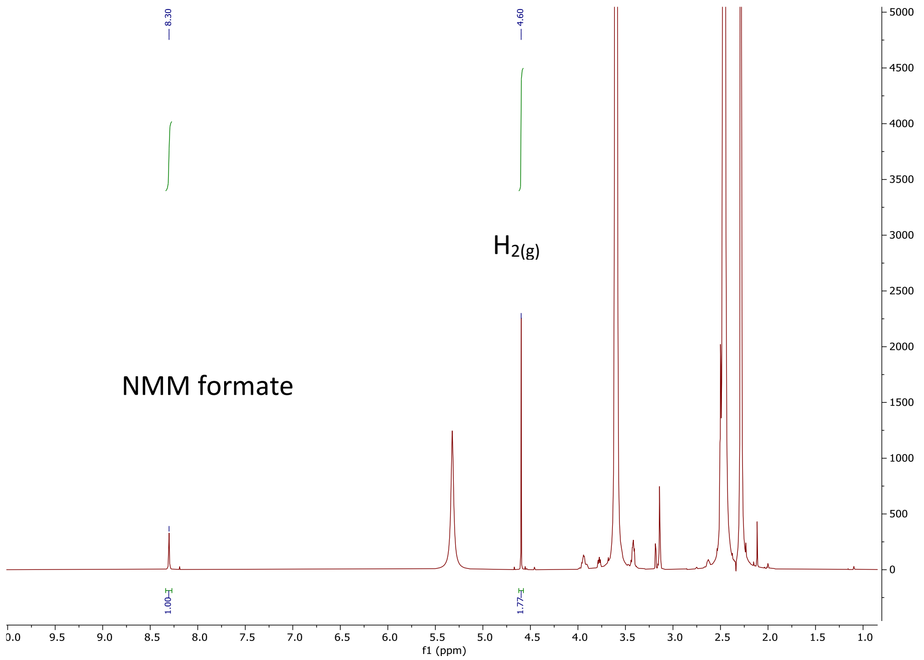

Figure S20: Result of the decomposition of formic acid by $In(OTf)_3$.

^{1}H NMR (400 MHz, DMSO-*d6*) δ: 8.30 (s, 1H, formic acid), 4.60 (s, 2H, H_2).

8. Formation of partially deuterated N-methylamines.

Under the standard reaction conditions some amines, particularly 1-acetylpiperazine, underwent N-methylation reaction instead of the expected N-formylation reaction. In depth analysis of the reaction indicate that CO_2 does not act as the C1 source in such reactions. Instead CO2 is reduced to [NMM][formate], which acts as an acid catalyst in amine N-methylation reaction using DMSO as the C1 source. Notably, the use of DMSO-d6 revealed partially deuteration of the produced 1-acetyl-4-methylpiperazine indicative of DMSO incorporation into the reaction side product. Such reaction was effectively inhibited by increase in the CO2 pressure, which promoted the desire N-formylation reaction.

8.1 Results:

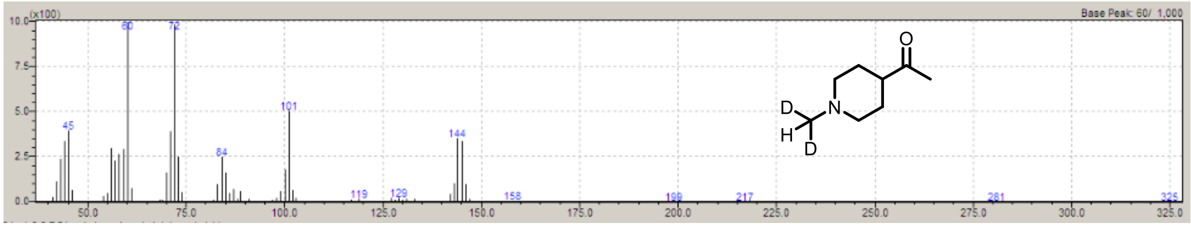

Figure S21: EI-MS analysis of the reaction product of DMSO-d6 and 4-acetylpiperizine. EI-MS (m/z) calculated: 144, found 144.

9. Computational details

9.1. Methods

Geometry optimizations were performed with the Gaussian16[1] set of programs, using the ωB97XD functional with the 6-31+G(d) basis set for H, C, O, N and S atoms, and the LANL2DZ pseudopotential for In, as recently reported for studying In(III)-catalyzed mechanisms.[2] After optimization, the stationary points were characterized by Hessian diagonalization and further harmonic frequency analyses. Solvent effects were included using the IEFPCM model with DMSO parameters (dielectric constant, $\varepsilon = 46.7$). Final refined Gibbs free energies were obtained from single-point calculations at the ωB97XD/def2TZVP level of theory. Intrinsic reaction coordinate (IRC) calculations were carried out at the ωB97XD/6-31+G(d)//LANL2DZ level of theory, for up to 80 steps. Visualization and graphics rendering were carried out with CYLView20.

9.2. Starting structures

Given the known propensity of DMSO to displace triflate ligands from $In(OTf)_3$ in solution, our modeling began with an $In(DMSO)_6$-coordinated complex (Fig. S22 a), based on the structure reported by Nava et al.[3] as a starting point. Compared to this, an alternative configuration featuring one coordinated triflate ligand (Fig. S22b) was found to be 4.5 kcal/mol higher in terms of Gibbs free energy, supporting the exclusion of triflates from the final mechanistic model due to their weak binding under catalytic conditions.

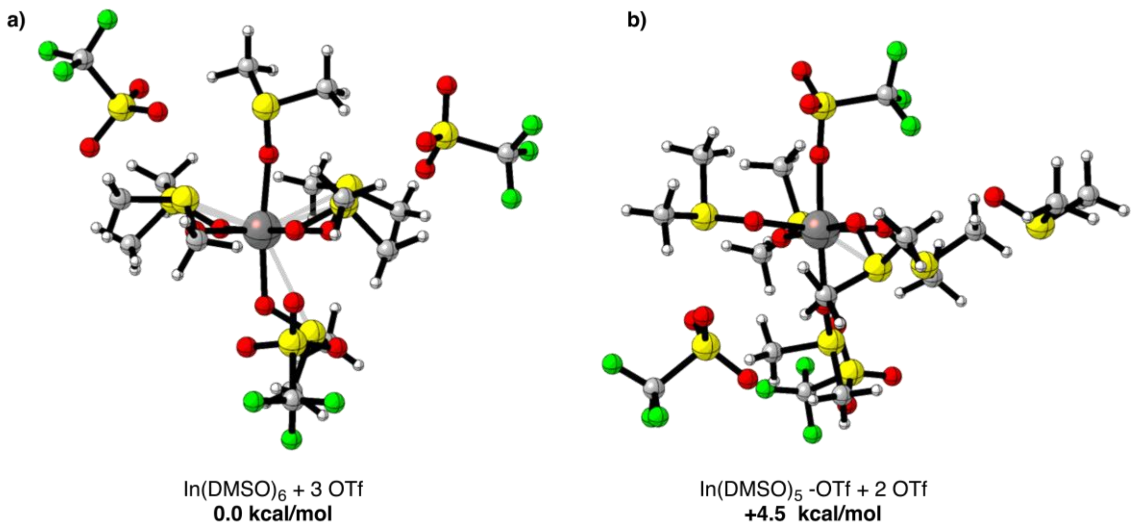

Figure S22. a) $In(DMSO)_6$-coordinated complex based on ref. 3, with the triflate ligands located in the secondary coordination sphere; b) alternative configuration featuring one triflate ligand coordinated to the indium center. Inset: relative Gibbs free energies calculated at the ωB97XD/def2TZVP level of theory in DMSO.

To explore potential reactive configurations, we computed the Gibbs free energies of several initial states, using the arrangement with fully separated reactants (Fig. S23-Ia) as the reference. Replacement of a DMSO ligand by a CO_2 molecule (Fig. S23-Ib) within the indium

coordination sphere was found to be slightly endergonic, while NMM binding (Fig. S23-Ic) was also endergonic but less than CO_2 binding. Additionally, given the known formation of carbamates from amines and CO_2, we evaluated a complex in which a NMM-carbamate interacts with the indium center (Fig. S23-Id). As shown in Fig. S21, species Ib), Ic) and d) exhibit comparable stability, suggesting that they may coexist in equilibrium at the experimental conditions (high temperatures and CO_2 pressure). However, in the carbamate-bound structure (Fig. S23-Id), the amine fragment sterically would hinder γ-terpinene from approaching the CO_2 carbon, impeding reduction. Moreover, NMM-bound complex (Fig. S23-Ic) resembles a frustrated Lewis pair (FLP), both structurally and energetically. The N-In bond length of 2.35Å aligns with values reported for NMM-aluminum adducts, although it is shorter than the optimal 3-5 Å range associated with effective FLP-mediated H_2 activation, indicating that the complex may be sterically or unfavorable for transfer hydrogenation. This limitation is further exacerbated by the steric bulk of γ-terpinene, which serves as the hydrogen donor. Consequently, the configuration shown in Fig. S23-Ib, where CO_2 acts as the Lewis base and serves as the hydride acceptor, appears to be the most realistic reactive species. In a similar fashion, morpholine was also considered as a Lewis base, demonstrating behavior analogous to NMM (Figure S23 II). Notable is morpholine binding to the indium center, which demonstrates almost identical binding energy as DMSO indicating that formation of mixed DMSO and morpholine bound indium complexes are possible for entropic reasons. Formation of mixed complexes then may affect the energy landscape of the reaction.

When DBU acts as the base, the configuration in which DBU coordinates to the axial position of the indium center (c, Fig. S23-IIIc), bottom) is more stable than the analogous NMM-bound configuration structure with NMM. However, the relative free energy of the CO_2-bound configuration (Fig. S23-IIIb), which corresponds to the productive pathway involving CO_2 coordination, remains nearly identical for both systems to that observed for NMM. This suggests that, despite the greater stronger binding stability of the DBU to indium adduct, the energy landscape for the productive pathway remains comparable to that of the NMM system, highlighting the similar reactivity of the two bases in the key catalytic step.

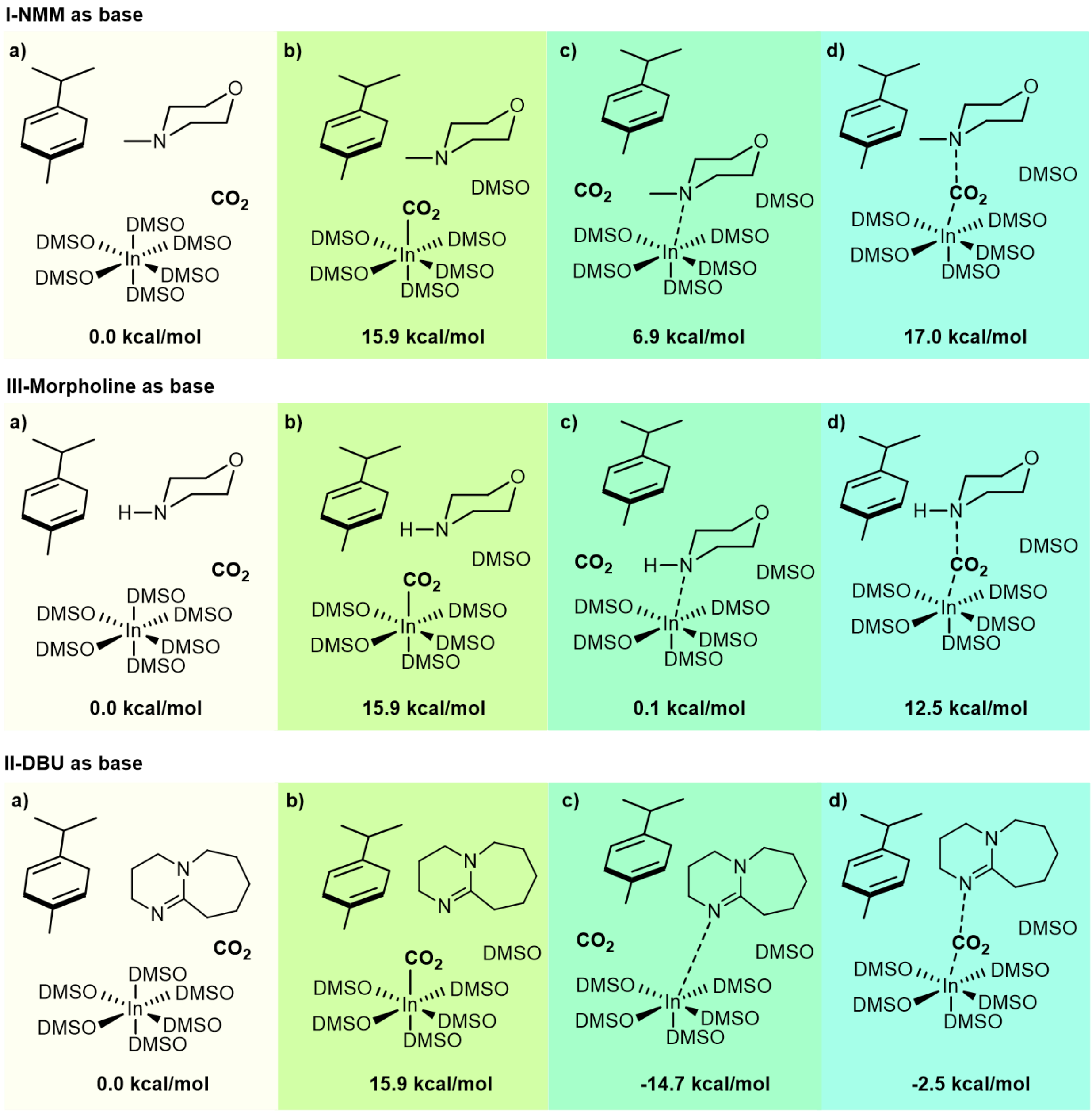

Figure S23. Configurations considered as the starting species for the catalysed transfer hydrogenation reactions, along with their relative Gibbs free energies computed at the ωB97XD/def2TZVP level of theory in DMSO.

9.3. IRC profile of an uncatalysed reaction

The transition state (TS) for the uncatalysed transfer hydrogenation reaction involving γ-terpinene, CO_2, and NMM corresponds to the hydride transfer stage, which represents the rate-limiting step of the process (Fig. S24). Following this TS, proton transfer from the resulting Wheland cation to NMM occurs without an energy barrier (see the shoulder in the IRC plot). It is noteworthy that the

Wheland-like cationic species does not constitute a local minimum on the potential energy surface, thereby ensuring the reaction proceeds through a single TS. The spontaneous proton transfer is driven by the gain of aromaticity, which facilitates the transformation of the cationic species into *p*-cymene, serving as the reaction's thermodynamic driving force.

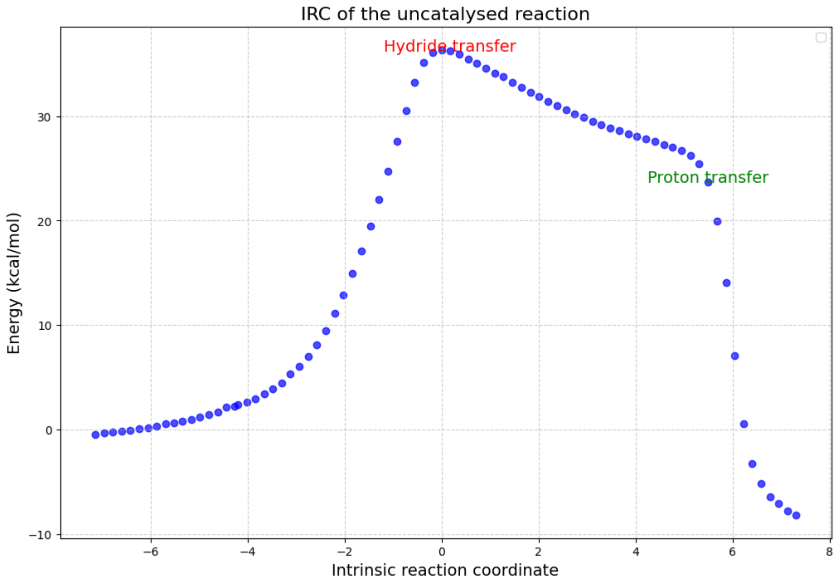

Figure S24. IRC profile of a model reaction in the absence of the Indium catalyst.

9.4. Geometries of the relevant TSs

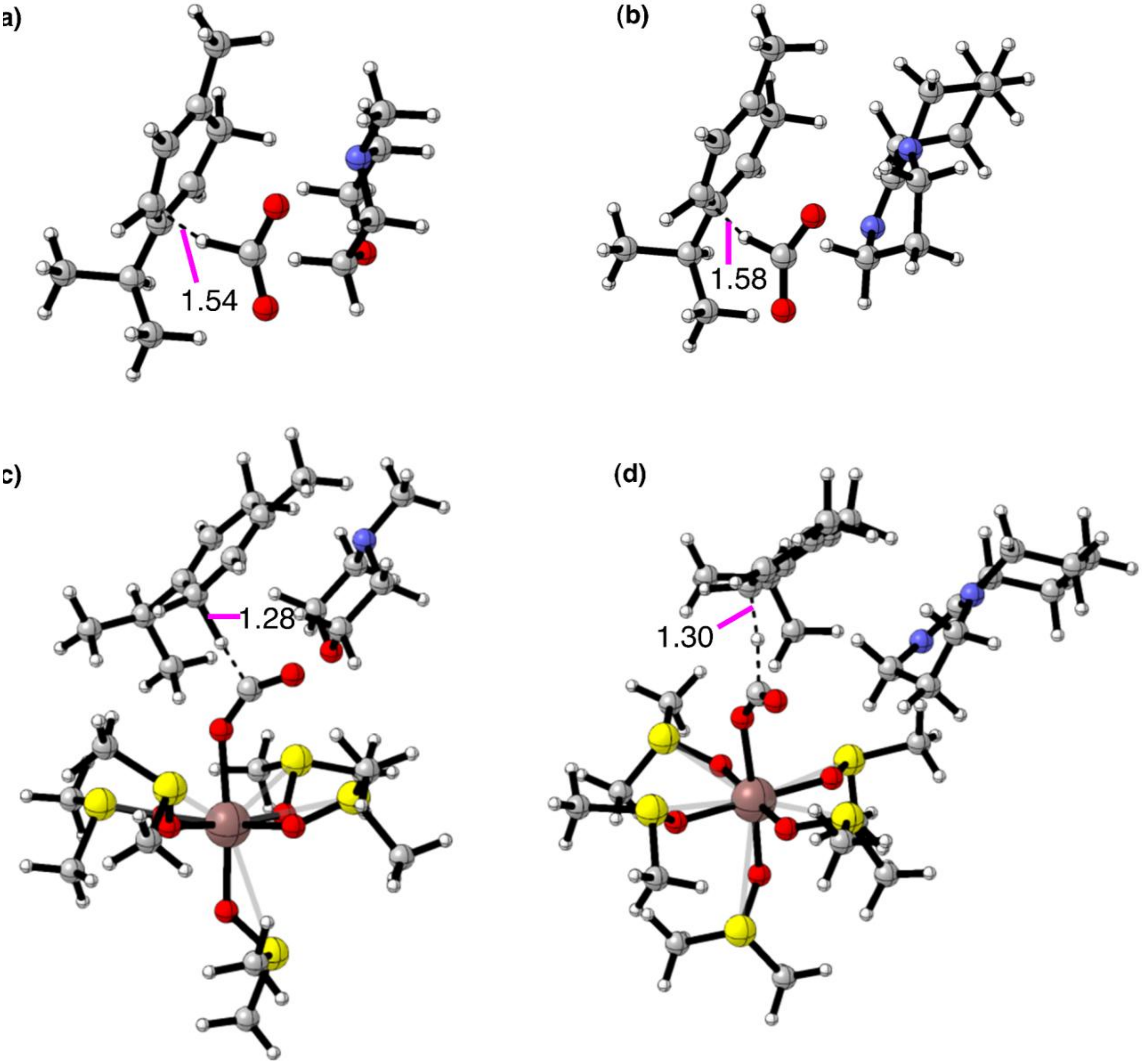

Figure S25: Geometries of the transition states (TS) for the uncatalysed (top) and catalysed (bottom) reaction pathways involving NMM (a and c) and DBU (b and d). The interatomic distances of the γ-terpinene C–H bond undergoing cleavage at each TS are indicated in angstroms (Å).

NMM-PC

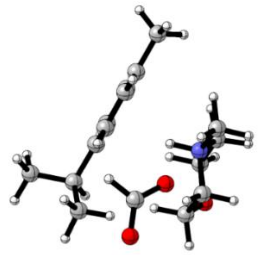

#p freq=noraman wb97xd/def2tzvp scrf=(solvent=dmso) nosymm geom=connec tivity gfinput iop(6/7=3)

Charge = 0, Multiplicity = 1, Point group = C1

Electronic Energy = -906.465015 Hartree

Sum of electronic and zero-point Energies = -906.048780 Hartree

Sum of electronic and thermal Energies = -906.026384 Hartree

Sum of electronic and thermal Enthalpies = -906.025440 Hartree

Sum of electronic and thermal Free Energies = -906.100298 Hartree

	Cartesian Coordinates				Cartesian Coordinates		
**Atoms**	***X***	***Y***	***Z***	**Atoms**	***X***	***Y***	***Z***
H	-3.391439	0.113513	2.052338	C	2.181174	1.839737	0.127201
C	1.888300	1.569225	1.470638	C	-0.469442	1.487826	0.864044
C	-0.178430	1.756213	-0.476447	H	3.216158	1.967344	-0.179851
H	-1.493718	1.321253	1.177995	C	2.988928	1.459268	2.494959
H	3.766554	0.762068	2.166962	H	3.470025	2.429484	2.654886
H	2.602155	1.111939	3.456227	C	-1.254204	1.900095	-1.539843
H	-0.831481	1.511160	-2.475964	C	-1.577228	3.386018	-1.764972
H	-2.316472	3.500703	-2.564270	H	-1.990315	3.828197	-0.851389
H	-0.681372	3.949915	-2.041914	C	-2.530195	1.111371	-1.236533
H	-3.205313	1.148854	-2.096425	H	-2.321795	0.060418	-1.013050
H	-3.067741	1.531576	-0.379906	C	0.546317	1.399930	1.819223
H	0.285256	1.178058	2.850471	C	1.168626	1.936602	-0.821821
H	1.426111	2.144933	-1.857769	N	0.944077	-1.557424	0.278996
C	-0.099897	-2.316126	-0.488021	C	2.291979	-1.727156	-0.351766
C	0.944013	-1.934767	1.720624	C	-0.037914	-1.929263	-1.953642
H	0.123350	-3.377401	-0.357437	H	-1.064917	-2.084650	-0.027216
C	2.227570	-1.404379	-1.837315	H	2.586586	-2.765672	-0.187364
H	2.989338	-1.063948	0.163451	H	-0.052267	-1.729680	2.115130
H	1.182940	-2.995602	1.797818	H	1.699525	-1.341374	2.234345
H	-0.754012	-2.530290	-2.514368	H	-0.296713	-0.867723	-2.085308
O	1.246184	-2.181828	-2.495264	H	3.190754	-1.636427	-2.292330
H	2.020802	-0.335715	-1.984580	H	0.687259	-0.561636	0.237076
C	-3.336129	-0.910250	1.606820	O	-2.223286	-1.490855	1.700186
O	-4.386474	-1.341491	1.076403				

NMM-RC

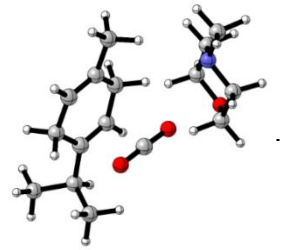

```
#p freq=noraman wb97xd/def2tzvp scrf=(solvent=dmso)
nosymm geom=connec tivity gfinput iop(6/7=3)
```

Charge = 0, Multiplicity = 1, Point group = C1

Electronic Energy = -906.455157 Hartree

Sum of electronic and zero-point Energies = -906.042652 Hartree

Sum of electronic and thermal Energies = -906.019831 Hartree

Sum of electronic and thermal Enthalpies = -906.018887 Hartree

Sum of electronic and thermal Free Energies = -906.096005 Hartree

	Cartesian Coordinates				Cartesian Coordinates		
**Atoms**	***X***	***Y***	***Z***	**Atoms**	***X***	***Y***	***Z***
H	-3.606575	1.001790	0.167506	C	-0.108178	0.444913	-1.289711
C	-0.725076	1.811458	-1.153954	C	-2.996690	0.827181	-0.729063
C	-2.333652	-0.527929	-0.639402	H	0.247061	0.309611	-2.324620
H	-3.715485	0.827181	-1.562016	C	0.223552	2.967443	-1.309079
H	0.730430	2.925863	-2.281032	H	-0.287475	3.930709	-1.226354
H	1.007420	2.921046	-0.543594	C	-3.190918	-1.707718	-0.219534
H	-2.605005	-2.615760	-0.410520	C	-4.486884	-1.806897	-1.035457
H	-5.018745	-2.732348	-0.792006	H	-5.160667	-0.971285	-0.815509
H	-4.282372	-1.804556	-2.111174	C	-3.501491	-1.669391	1.284991
H	-4.090373	-2.545757	1.576531	H	-2.579021	-1.665591	1.873921
H	-4.077817	-0.776674	1.551785	C	-2.028241	1.965967	-0.900277
H	-2.440616	2.969880	-0.806302	C	-1.032901	-0.675437	-0.912646
H	-0.590232	-1.670083	-0.850408	N	3.102056	0.330759	0.027375
C	3.189138	-0.436679	1.261581	C	3.676853	-0.425856	-1.075989
C	3.703795	1.643870	0.157782	C	2.497350	-1.778895	1.085739
H	4.244043	-0.605638	1.555408	H	2.698178	0.119804	2.065861
C	2.989849	-1.778270	-1.200384	H	4.764517	-0.580499	-0.933011
H	3.537000	0.133514	-2.007522	H	3.211953	2.195232	0.964898
H	4.786219	1.594294	0.379775	H	3.569743	2.203253	-0.773160
H	2.611308	-2.391995	1.982117	H	1.425066	-1.618872	0.896516
O	3.068711	-2.513968	0.012052	H	3.473947	-2.384880	-1.968507
H	1.934364	-1.635191	-1.474436	H	0.805302	0.403373	-0.677707
C	-1.007354	1.232840	2.183597	O	0.120096	1.013517	1.991846
O	-2.128991	1.454035	2.408949				

NMM-TS

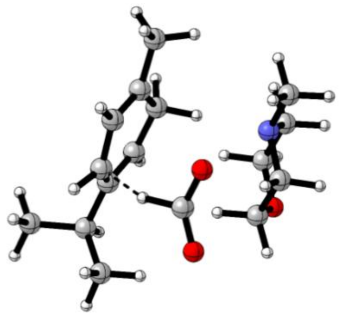

```
#p freq=noraman wb97xd/def2tzvp scrf=(solvent=dmso)
nosymm geom=connec tivity gfinput iop(6/7=3)
```

Charge = 0, Multiplicity = 1, Point group = C1

Electronic Energy = -906.384522 Hartree

Number of imaginary frequencies = 1, vi = -732.05

Sum of electronic and zero-point Energies = -905.976886 Hartree

Sum of electronic and thermal Energies = -905.955061 Hartree

Sum of electronic and thermal Enthalpies = -905.954117 Hartree

Sum of electronic and thermal Free Energies = -906.027602 Hartree

Atoms	Cartesian Coordinates			Atoms	Cartesian Coordinates		
	X	*Y*	*Z*		*X*	*Y*	*Z*
H	-1.628616	0.405884	1.464614	C	-0.032202	1.163662	-1.488090
C	-0.573494	2.328958	-0.754190	C	-2.294263	0.897376	0.171109
C	-1.986597	-0.182211	-0.753331	H	0.346776	1.444613	-2.477678
H	-3.260460	0.869643	0.671806	C	0.143008	3.633297	-0.889803
H	0.050670	4.008926	-1.914822	H	-0.248020	4.385070	-0.202106
H	1.211954	3.497393	-0.695674	C	-2.873489	-1.409027	-0.807002
H	-2.379620	-2.123041	-1.476399	C	-4.244035	-1.070384	-1.410962
H	-4.852252	-1.976037	-1.498781	H	-4.793424	-0.362852	-0.779352
H	-4.138321	-0.627549	-2.405760	C	-3.024779	-2.075843	0.565551
H	-3.577915	-3.014462	0.464344	H	-2.053302	-2.288536	1.019206
H	-3.582178	-1.437526	1.260352	C	-1.672135	2.176957	0.032409
H	-2.057322	3.008198	0.612479	C	-0.904898	-0.027551	-1.546337
H	-0.630659	-0.814639	-2.244448	N	2.599047	0.206145	0.002738
C	2.357418	-0.750393	1.078420	C	3.402520	-0.411941	-1.047001
C	3.195492	1.434374	0.501212	C	1.684912	-1.994991	0.523982
H	3.306126	-1.034571	1.571115	H	1.711011	-0.285167	1.827889
C	2.717510	-1.674087	-1.551060	H	4.413346	-0.663236	-0.675543
H	3.513502	0.295593	-1.875815	H	2.525710	1.886450	1.238568
H	4.176848	1.261377	0.976422	H	3.334203	2.138258	-0.325123
H	1.560556	-2.746401	1.306052	H	0.692717	-1.737066	0.122296
O	2.473323	-2.592338	-0.496925	H	3.350088	-2.188425	-2.277121
H	1.766419	-1.411977	-2.038093	H	0.897146	0.847286	-0.930137
C	-0.815084	0.291529	2.375715	O	-0.109638	1.292672	2.461223
O	-0.904636	-0.795155	2.943054				

In-NMM-PC'

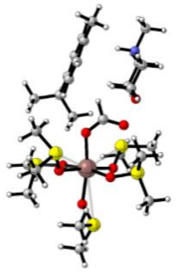

```
#p freq=noraman wb97xd/def2tzvp
scrf=(solvent=dmso) nosymm gfinput iop (6/7=3)
```

Charge = 3, Multiplicity = 1, Point group = C1

Electronic Energy = -3862.63068230 Hartree

Sum of electronic and zero-point Energies = -3861.800564 Hartree

Sum of electronic and thermal Energies = -3861.744021 Hartree

Sum of electronic and thermal Enthalpies = -3861.743077 Hartree

Sum of electronic and thermal Free Energies = -3861.890555 Hartree

	**Cartesian Coordinates**				**Cartesian Coordinates**		
**Atoms**	***X***	***Y***	***Z***	**Atoms**	***X***	***Y***	***Z***
H	1.672083	-0.171342	2.084818	C	5.933211	1.747213	0.298773
C	5.340228	1.480920	1.536892	C	3.178610	1.962822	0.527522
C	3.770615	2.217293	-0.717028	H	7.015920	1.696880	0.199559
H	2.099253	2.035492	0.637833	C	6.162422	1.108014	2.744032
H	7.207933	0.934469	2.477828	H	6.133965	1.906505	3.492543
H	5.774089	0.202453	3.220412	C	2.938107	2.615450	-1.921472
H	3.615202	2.653593	-2.783558	C	2.337254	4.015704	-1.735684
H	1.810067	4.331317	-2.642230	H	1.625253	4.027626	-0.902437
H	3.116241	4.754350	-1.524294	C	1.846716	1.582086	-2.227361
H	1.324738	1.840481	-3.154854	H	2.275759	0.583537	-2.354083
H	1.101448	1.525722	-1.426339	C	3.947011	1.599908	1.629023
H	3.461573	1.411191	2.583536	C	5.159021	2.107766	-0.809585
H	5.650903	2.306035	-1.758856	N	6.105653	-1.365607	-0.658156
C	5.030941	-2.049432	0.132837	C	5.913052	-1.619971	-2.124825
C	7.472553	-1.739893	-0.199308	C	3.668317	-1.631475	-0.390543
H	5.187285	-3.124771	0.025358	H	5.150953	-1.766433	1.179868
C	4.501456	-1.234362	-2.535149	H	6.098313	-2.682795	-2.292257
H	6.651557	-1.029214	-2.668770	H	7.561573	-1.509361	0.861625
H	7.616787	-2.806818	-0.366168	H	8.200182	-1.165517	-0.771093
H	2.895266	-2.181497	0.148045	H	3.504096	-0.557230	-0.221780
O	3.534072	-1.929815	-1.770239	H	4.347370	-1.499712	-3.581128
H	4.360479	-0.150011	-2.424609	H	5.995582	-0.352102	-0.496453
C	0.831301	-0.625910	1.536428	O	0.760690	-1.852090	1.422606
O	-0.001972	0.227174	1.053270	S	-1.912499	-3.211173	1.520385
O	-2.446333	-1.742621	1.418896	C	-3.429164	-4.170908	1.562055
C	-1.421510	-3.346571	3.239375	In	-1.821430	-0.194490	0.119328
H	-3.882484	-4.104460	0.572643	H	-3.170760	-5.209237	1.780098
H	-4.097836	-3.765440	2.323750	H	-0.520246	-2.745553	3.353881
H	-2.231518	-2.979295	3.871805	H	-1.202742	-4.395373	3.450190

O	-1.090234	-1.538838	-1.300384	O	-3.740449	-0.283938	-0.747117
O	-1.530092	1.388301	-1.234968	O	-2.659157	1.200701	1.487996
S	0.427138	-1.713236	-1.647840	S	-4.922654	-1.229749	-0.373006
S	-1.875017	2.891852	-0.989777	S	-2.122054	1.425921	2.939649
C	0.446572	-1.589322	-3.435336	C	0.702076	-3.470860	-1.444473
C	-5.530487	-0.616414	1.202701	C	-6.219403	-0.633920	-1.458441
C	-1.522746	3.612574	-2.592953	C	-3.670243	2.974645	-1.012814
C	-1.000220	2.819523	2.789802	C	-3.516086	2.229300	3.730672
H	0.188241	-0.559932	-3.686517	H	1.459829	-1.813886	-3.774266
H	-0.276915	-2.288196	-3.858266	H	0.638327	-3.668490	-0.374234
H	-0.063144	-4.020085	-1.995586	H	1.703553	-3.693515	-1.818024
H	-4.772422	-0.866663	1.944996	H	-6.467075	-1.130451	1.428893
H	-5.673832	0.463382	1.138735	H	-5.919766	-0.867721	-2.480227
H	-6.330844	0.443146	-1.323011	H	-7.143335	-1.160275	-1.211024
H	-0.449381	3.522375	-2.756740	H	-1.807257	4.666245	-2.566080
H	-2.078617	3.071230	-3.360325	H	-4.017670	2.485740	-0.102641
H	-4.041119	2.456243	-1.898189	H	-3.956464	4.028741	-1.016822
H	-0.156541	2.466914	2.195230	H	-0.669062	3.099431	3.792136
H	-1.512009	3.648385	2.297491	H	-4.305948	1.485218	3.832237
H	-3.844518	3.063690	3.108685	H	-3.199351	2.574391	4.716643

In-NMM-RC'

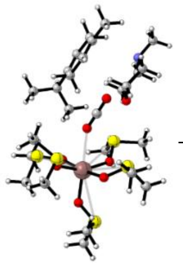

```
#p freq=noraman wb97xd/def2tzvp scrf=(solvent=dmso)
nosymm geom=connec tivity gfinput iop(6/7=3)
```

Charge = 3, Multiplicity = 1, Point group = C1

Electronic Energy = -3862.565920 Hartree

Sum of electronic and zero-point Energies = -3861.739853 Hartree

Sum of electronic and thermal Energies = -3861.683456 Hartree

Sum of electronic and thermal Enthalpies = -3861.682512 Hartree

Sum of electronic and thermal Free Energies = -3861.830177 Hartree

Atoms	Cartesian Coordinates			Atoms	Cartesian Coordinates		
	X	*Y*	*Z*		*X*	*Y*	*Z*
H	2.565042	1.414260	0.566567	C	6.280128	1.495394	0.144960
C	5.648593	1.310842	1.499893	C	3.414541	2.108618	0.665410
C	4.046674	2.296469	-0.692600	H	7.160177	2.149231	0.237725
H	2.962496	3.051722	1.004000	C	6.557195	0.783227	2.575579
H	7.396726	1.468453	2.745026	H	6.029811	0.643392	3.523237
H	6.991918	-0.178761	2.276078	C	3.148655	2.728628	-1.834931
H	3.784972	2.803887	-2.725461	C	2.504464	4.102200	-1.602408
H	1.958881	4.424942	-2.495739	H	1.790780	4.074360	-0.770992
H	3.260647	4.860474	-1.376266	C	2.075531	1.667359	-2.125517
H	1.599201	1.859920	-3.092752	H	2.510343	0.664543	-2.163227
H	1.295050	1.662913	-1.355258	C	4.360520	1.594874	1.715921
H	3.942315	1.445257	2.710210	C	5.340848	2.030651	-0.895426
H	5.756732	2.162559	-1.894034	N	6.210602	-1.759355	-1.246585
C	5.247413	-2.152847	-0.227011	C	5.668660	-2.031103	-2.571709
C	7.497242	-2.400651	-1.042958	C	3.923856	-1.440496	-0.448863
H	5.082842	-3.248373	-0.233357	H	5.634896	-1.876759	0.760022
C	4.348018	-1.300562	-2.750957	H	5.510659	-3.116240	-2.727958
H	6.378252	-1.682824	-3.329481	H	7.888859	-2.133259	-0.056572
H	7.434303	-3.503040	-1.104551	H	8.207170	-2.054433	-1.799787
H	3.176744	-1.801853	0.262127	H	4.055209	-0.361034	-0.313270
O	3.409436	-1.691836	-1.753888	H	3.897192	-1.540105	-3.716418
H	4.515482	-0.215595	-2.687981	H	6.668626	0.525540	-0.205569
C	0.943242	-0.039214	2.347703	O	1.781730	-0.380107	3.068712
O	0.097917	0.313257	1.617822	S	-1.821183	-2.582262	2.317864
O	-2.674215	-1.368462	1.792914	C	-2.490843	-3.987918	1.431304
C	-2.527919	-2.834860	3.942753	In	-2.182270	-0.058630	0.268082
H	-2.237975	-3.843803	0.380203	H	-2.009358	-4.890112	1.813642
H	-3.571181	-4.027003	1.578924	H	-2.274826	-1.958519	4.539985
H	-3.608924	-2.948581	3.848861	H	-2.071636	-3.727405	4.375095

O	-1.032415	-1.537967	-0.525886	O	-3.998414	-0.288976	-0.635648
O	-1.489005	1.295859	-1.102389	O	-2.602277	1.523836	1.500695
S	0.441568	-1.518163	-1.088737	S	-4.954306	-1.531568	-0.506699
S	-1.657709	2.863779	-1.097502	S	-2.589914	1.605866	3.070152
C	0.221385	-1.186135	-2.834889	C	0.819514	-3.267450	-1.121050
C	-5.939220	-1.164983	0.946570	C	-6.143112	-1.212711	-1.805527
C	-1.299945	3.254963	-2.808694	C	-3.434118	3.133233	-1.094863
C	-1.853018	3.216763	3.338790	C	-4.302401	1.950412	3.468889
H	-0.146815	-0.162386	-2.916576	H	1.199701	-1.278839	-3.309470
H	-0.495570	-1.896971	-3.248501	H	0.872297	-3.603964	-0.085162
H	0.031044	-3.790805	-1.663935	H	1.792502	-3.373757	-1.604054
H	-5.268290	-1.250780	1.802263	H	-6.732103	-1.912144	1.018322
H	-6.350737	-0.158008	0.859539	H	-5.617514	-1.307957	-2.755606
H	-6.553690	-0.209556	-1.681503	H	-6.925557	-1.970976	-1.734558
H	-0.243614	3.050323	-2.975403	H	-1.499635	4.317398	-2.960092
H	-1.925899	2.637624	-3.454481	H	-3.799328	2.854276	-0.106444
H	-3.891566	2.523106	-1.875403	H	-3.606346	4.197256	-1.270479
H	-0.805281	3.143140	3.046404	H	-1.927916	3.452954	4.401865
H	-2.379377	3.954218	2.730858	H	-4.872350	1.050970	3.237339
H	-4.647460	2.799018	2.876487	H	-4.364473	2.162050	4.538280

In-NMM-TS'

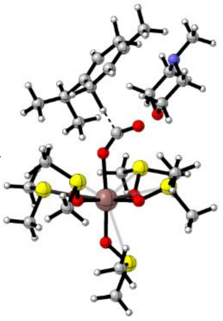

```
#p freq=noraman wb97xd/def2tzvp scrf=(solvent=dmso)
nosymm geom=connec tivity gfinput iop(6/7=3)
```

Charge = 3, Multiplicity = 1, Point group = C1

Electronic Energy = -3862.523804 Hartree

Number of imaginary frequencies = 1, vi = -815.51

Sum of electronic and zero-point Energies = -3861.701894 Hartree

Sum of electronic and thermal Energies = -3861.645666 Hartree

Sum of electronic and thermal Enthalpies = -3861.644721 Hartree

Sum of electronic and thermal Free Energies = -3861.791972 Hartree

	Cartesian Coordinates				Cartesian Coordinates		
Atoms	*X*	*Y*	*Z*	Atoms	*X*	*Y*	*Z*
H	1.898200	1.174336	0.846218	C	5.515340	1.111797	0.617846
C	4.850667	0.997604	1.948900	C	2.815361	2.049805	1.049217
C	3.459837	2.244512	-0.266435	H	6.494969	1.595452	0.738501
H	2.128918	2.843574	1.351009	C	5.642122	0.368943	3.054636
H	6.591955	0.894655	3.198433	H	5.092187	0.368599	3.997916
H	5.889086	-0.667391	2.796546	C	2.701666	2.891418	-1.410669
H	3.460563	3.207042	-2.135374	C	1.904807	4.133566	-0.997323
H	1.515377	4.637602	-1.886893	H	1.043456	3.870777	-0.373229
H	2.526120	4.846009	-0.446056	C	1.792469	1.872462	-2.115308
H	1.410123	2.294800	-3.050231	H	2.337604	0.955246	-2.360204
H	0.935547	1.604955	-1.487490	C	3.602265	1.471702	2.136933
H	3.135977	1.418265	3.115822	C	4.725418	1.817610	-0.424398
H	5.224467	1.987987	-1.376297	N	5.747634	-1.919328	-0.760935
C	4.601062	-2.451590	-0.035580	C	5.531854	-2.052627	-2.197537
C	6.990828	-2.541674	-0.337239	C	3.331380	-1.744671	-0.477970
H	4.489324	-3.540072	-0.199882	H	4.744451	-2.288043	1.038200
C	4.246335	-1.346378	-2.598830	H	5.473519	-3.116855	-2.495491
H	6.373402	-1.598619	-2.730501	H	7.139282	-2.374028	0.733865
H	7.002712	-3.630476	-0.523735	H	7.829895	-2.090342	-0.874307
H	2.462923	-2.191926	0.009716	H	3.378385	-0.678885	-0.208444
O	3.132470	-1.863355	-1.880349	H	4.034067	-1.499992	-3.658546
H	4.339160	-0.267830	-2.404151	H	5.754997	0.100765	0.231933
C	0.895016	0.303268	1.131763	O	1.282356	-0.510559	1.910836
O	-0.074574	0.678713	0.479670	S	-1.209452	-2.556762	1.793711
O	-2.170407	-1.341301	1.533387	C	-2.296371	-3.979080	1.711241
C	-0.933215	-2.451034	3.559188	In	-2.133739	-0.035873	-0.098776
H	-2.600501	-4.091093	0.670122	H	-1.728085	-4.856364	2.027721
H	-3.158667	-3.816059	2.359830	H	-0.344272	-1.550307	3.732512

H	-1.896147	-2.398270	4.069529	H	-0.367989	-3.332921	3.866980
O	-1.281965	-1.484338	-1.297269	O	-4.069161	-0.518170	-0.629074
O	-1.985824	1.344678	-1.629945	O	-2.804299	1.477391	1.165410
S	0.212261	-1.573777	-1.778504	S	-4.910522	-1.726318	-0.091760
S	-2.093266	2.904326	-1.478979	S	-2.263189	1.815056	2.597684
C	0.160330	-0.938434	-3.453118	C	0.396807	-3.327019	-2.091401
C	-5.540385	-1.151283	1.487456	C	-6.389718	-1.611688	-1.094581
C	-1.770242	3.445558	-3.154845	C	-3.857576	3.215747	-1.376324
C	-1.625660	3.479396	2.401633	C	-3.767238	2.146957	3.512285
H	-0.114756	0.114880	-3.381138	H	1.159598	-1.042901	-3.879646
H	-0.580248	-1.495084	-4.029104	H	0.346874	-3.833926	-1.127188
H	-0.408436	-3.658137	-2.748851	H	1.379958	-3.473022	-2.540815
H	-4.687919	-1.118098	2.167144	H	-6.276560	-1.873407	1.846395
H	-5.984596	-0.162793	1.359305	H	-6.103358	-1.840686	-2.121036
H	-6.797477	-0.602277	-1.018860	H	-7.103809	-2.353449	-0.732038
H	-0.722275	3.236497	-3.367236	H	-1.952150	4.520585	-3.205706
H	-2.423691	2.900401	-3.837345	H	-4.187794	2.818025	-0.415774
H	-4.358493	2.715357	-2.206666	H	-4.013109	4.296029	-1.412655
H	-0.754199	3.410167	1.750493	H	-1.327163	3.852488	3.383188
H	-2.396124	4.111265	1.956641	H	-4.275416	1.193710	3.655935
H	-4.385652	2.838188	2.937510	H	-3.492075	2.571807	4.479586

DBU-PC

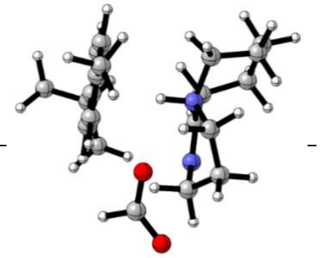

#p freq=noraman wb97xd/def2tzvp scrf=(solvent=dmso) nosymm geom=connec tivity gfinput iop(6/7=3)

Charge = 0, Multiplicity = 1, Point group = C1

Electronic Energy = -1041.432233 Hartree

Sum of electronic and zero-point Energies = -1040.933289 Hartree

Sum of electronic and thermal Energies = -1040.908024 Hartree

Sum of electronic and thermal Enthalpies = -1040.907080 Hartree

Sum of electronic and thermal Free Energies = -1040.987942 Hartree

	Cartesian Coordinates				Cartesian Coordinates		
**Atoms**	***X***	***Y***	***Z***	**Atoms**	***X***	***Y***	***Z***
H	-1.332569	-1.193136	4.128916	C	2.230753	1.682476	0.619400
C	1.797185	0.957689	1.737552	C	-0.476226	1.675494	1.228843
C	-0.044848	2.393124	0.109089	H	3.285896	1.683632	0.358746
H	-1.531268	1.643529	1.481473	C	2.760901	0.192160	2.606829
H	3.150947	0.835001	3.403757	H	2.258283	-0.659147	3.074835
H	3.615234	-0.169986	2.028033	C	-0.986753	3.198187	-0.770805
H	-0.630461	3.085305	-1.803259	C	-0.894411	4.690311	-0.412242
H	-1.531347	5.283607	-1.076208	H	-1.227513	4.855098	0.618655
H	0.131573	5.059824	-0.501652	C	-2.440837	2.722676	-0.724712
H	-3.025433	3.248778	-1.485203	H	-2.523899	1.647240	-0.909982
H	-2.900647	2.937364	0.246388	C	0.426342	0.966670	2.024699
H	0.064262	0.384330	2.867347	C	1.326520	2.380561	-0.177759
H	1.692280	2.919582	-1.048742	H	0.738581	-0.315450	0.126637
C	-0.902216	-2.174337	3.805857	O	0.318393	-2.166981	3.506179
O	-1.699753	-3.143034	3.778179	C	1.390815	-1.349377	-3.830300
C	1.864621	-2.043921	-2.551694	C	1.911655	-1.128257	-1.331135
C	-0.061081	-0.877506	-3.797610	N	0.619851	-1.082220	-0.553937
C	-0.397722	0.056469	-2.620288	C	-0.609301	-0.669637	-1.324612
H	2.045272	-0.490094	-4.024495	H	1.248094	-2.927374	-2.344248
H	2.163849	-0.103435	-1.606438	H	-0.739268	-1.738569	-3.768969
H	-1.343795	0.563789	-2.815846	H	1.520649	-2.037295	-4.671477
H	2.881058	-2.415910	-2.706178	H	2.655401	-1.460790	-0.605520
H	-0.278142	-0.336343	-4.723137	H	0.368975	0.831548	-2.511464
N	-1.758452	-0.887368	-0.861162	C	0.466953	-2.337666	0.309762
C	-1.915641	-1.647198	0.370280	C	-0.963035	-2.832786	0.369527
H	-2.955028	-1.968694	0.442039	H	-1.716012	-0.990429	1.227519
H	-1.193705	-3.473329	-0.487235	H	-1.081594	-3.414527	1.286446
H	0.809033	-2.055319	1.308853	H	1.137260	-3.089884	-0.102937

DBU-RC

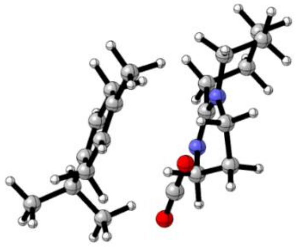

```
#p freq=noraman wb97xd/def2tzvp
scrf=(solvent=dmso) nosymm geom=connec tivity
gfinput iop(6/7=3)
```

Charge = 0, Multiplicity = 1, Point group = C1

Electronic Energy = -1041.453187 Hartree

Sum of electronic and zero-point Energies = -1040.956445 Hartree

Sum of electronic and thermal Energies = -1040.930789 Hartree

Sum of electronic and thermal Enthalpies = -1040.929844 Hartree

Sum of electronic and thermal Free Energies = -1041.012687 Hartree

Atoms	Cartesian Coordinates			Atoms	Cartesian Coordinates		
	X	*Y*	*Z*		*X*	*Y*	*Z*
H	-1.481191	1.528454	2.362766	C	1.600204	1.558997	0.203222
C	1.611364	0.977436	1.592517	C	-0.562534	2.102222	2.171246
C	-0.591329	2.656872	0.767206	H	2.523136	2.138621	0.048548
H	-0.594866	2.928670	2.895663	C	2.792668	0.112206	1.931048
H	3.727096	0.680551	1.849213	H	2.722108	-0.295593	2.942903
H	2.872619	-0.724967	1.226708	C	-1.800018	3.478459	0.357698
H	-1.573808	3.908115	-0.626477	C	-2.075651	4.641005	1.321058
H	-2.868434	5.283008	0.923515	H	-2.406519	4.278596	2.300714
H	-1.181511	5.255362	1.470054	C	-3.051057	2.600516	0.197126
H	-3.897305	3.203867	-0.148668	H	-2.877249	1.801136	-0.529050
H	-3.340950	2.139118	1.147958	C	0.631706	1.238421	2.462933
H	0.695459	0.826220	3.469637	C	0.399902	2.409536	-0.093940
H	0.341918	2.821959	-1.100717	H	1.656297	0.738182	-0.525567
C	-0.759343	-1.565136	3.585714	O	0.253442	-2.123891	3.722544
O	-1.776180	-1.013623	3.449525	C	2.188644	-2.398223	-4.016611
C	2.369764	-3.000136	-2.622510	C	2.227891	-1.975098	-1.493474
C	0.850897	-1.690480	-4.244694	N	0.848620	-1.643935	-1.149900
C	0.586986	-0.495476	-3.310950	C	0.042414	-0.880337	-1.955022
H	2.999048	-1.677136	-4.192940	H	1.643878	-3.806253	-2.454875
H	2.784549	-1.062635	-1.740744	H	0.025403	-2.407777	-4.148123
H	-0.162582	0.162883	-3.752824	H	2.307170	-3.187859	-4.767181
H	3.367234	-3.449922	-2.558248	H	2.684126	-2.368916	-0.581363
H	0.826974	-1.326248	-5.277379	H	1.504681	0.097072	-3.197934
N	-1.151631	-0.481231	-1.654909	C	0.322197	-2.283874	0.055142
C	-1.646994	-0.849254	-0.336478	C	-1.196225	-2.251928	0.056033
H	-2.739215	-0.778096	-0.340179	H	-1.282321	-0.122802	0.406133
H	-1.585895	-2.973674	-0.671142	H	-1.579244	-2.540412	1.038691
H	0.717095	-1.769337	0.941935	H	0.684171	-3.317904	0.084138

DBU-TS

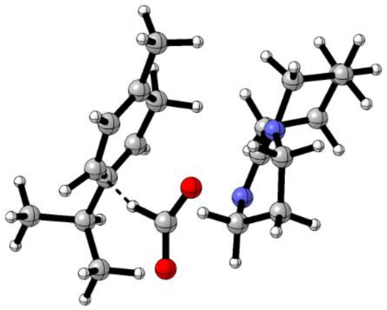

`#p freq=noraman wb97xd/def2tzvp scrf=(solvent=dmso) nosymm geom=connec tivity gfinput iop(6/7=3)`

Charge = 0, Multiplicity = 1, Point group = C1

Electronic Energy = -1041.379486 Hartree

Number of imaginary frequencies = 1, vi = -606.97

Sum of electronic and zero-point Energies = -1040.887119 Hartree

Sum of electronic and thermal Energies = -1040.862439 Hartree

Sum of electronic and thermal Enthalpies = -1040.861495 Hartree

Sum of electronic and thermal Free Energies = -1040.940613 Hartree

	**Cartesian Coordinates**				**Cartesian Coordinates**		
**Atoms**	***X***	***Y***	***Z***	**Atoms**	***X***	***Y***	***Z***
H	2.584936	0.581269	2.160626	C	5.261767	1.064005	-0.144626
C	5.620599	0.936089	1.290447	C	3.464928	1.870665	1.884141
C	3.134688	2.182172	0.506880	H	6.100940	1.482470	-0.716408
H	2.894500	2.369486	2.665007	C	6.946409	0.333380	1.622539
H	7.756356	0.933710	1.194771	H	7.095246	0.253089	2.700432
H	7.023180	-0.666272	1.180823	C	1.860234	2.936023	0.185447
H	1.798087	2.992291	-0.907401	C	1.915353	4.371115	0.728175
H	1.017219	4.920806	0.430510	H	1.961601	4.383452	1.823051
H	2.789199	4.905631	0.344034	C	0.613238	2.200675	0.690925
H	-0.287235	2.740825	0.383409	H	0.558719	1.184196	0.292347
H	0.602661	2.129823	1.783883	C	4.747316	1.354855	2.242792
H	4.989115	1.256329	3.295213	C	4.008785	1.792507	-0.446432
H	3.799022	1.996141	-1.492772	H	5.156736	0.040657	-0.555311
C	2.268398	-0.547592	2.475329	O	3.254472	-1.278046	2.556958
O	1.052782	-0.670268	2.629135	C	6.062915	-2.557254	-4.477635
C	6.148601	-3.192250	-3.088652	C	5.976454	-2.182696	-1.950001
C	4.757016	-1.813921	-4.769189	N	4.587947	-1.830136	-1.659286
C	4.453479	-0.643415	-3.815959	C	3.822966	-1.070619	-2.511522
H	6.898531	-1.852364	-4.589750	H	5.391747	-3.979454	-2.977810
H	6.554881	-1.275604	-2.162916	H	3.913179	-2.515858	-4.750048
H	3.738366	0.037270	-4.280505	H	6.207693	-3.333414	-5.237438
H	7.127125	-3.673476	-2.979690	H	6.385268	-2.591425	-1.022229
H	4.807945	-1.416861	-5.788602	H	5.369315	-0.067572	-3.629297
N	2.606616	-0.694377	-2.281033	C	3.976138	-2.555272	-0.539570
C	2.018070	-1.126568	-1.021838	C	2.460944	-2.533937	-0.639195
H	0.928581	-1.069320	-1.107334	H	2.308125	-0.427540	-0.222914
H	2.120794	-3.239521	-1.405922	H	2.029217	-2.837682	0.318058

H	4.293260	-2.101574	0.408539	H	4.345735	-3.586728	-0.557447

In-DBU-PC'

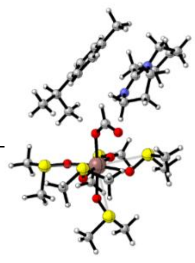

```
#p freq=noraman wb97xd/def2tzvp
scrf=(solvent=dmso) nosymm gfinput iop (6/7=3)
```

Charge = 3, Multiplicity = 1, Point group = C1

Electronic Energy = -3997.64555980 Hartree

Sum of electronic and zero-point Energies = -3996.732635 Hartree

Sum of electronic and thermal Energies = -3996.672976 Hartree

Sum of electronic and thermal Enthalpies = -3996.672032 Hartree

Sum of electronic and thermal Free Energies = -3996.826189 Hartree

Atoms	Cartesian Coordinates *X*	*Y*	*Z*	Atoms	Cartesian Coordinates *X*	*Y*	*Z*
H	2.862627	-1.343790	2.854222	C	5.802440	1.025385	-0.285883
C	6.217797	-0.050336	0.508158	C	4.671758	0.715724	2.223897
C	4.247429	1.782299	1.428065	H	6.246642	1.170222	-1.268147
H	4.253189	0.576441	3.215674	C	7.289891	-0.998846	0.033411
H	8.286476	-0.594966	0.242911	H	7.215548	-1.968483	0.533794
H	7.228160	-1.163727	-1.046100	C	3.164461	2.761331	1.855147
H	3.526962	3.766255	1.601293	C	2.863940	2.736012	3.355133
H	2.187936	3.557131	3.613266	H	2.377313	1.797320	3.644072
H	3.771323	2.856193	3.955334	C	1.877193	2.510808	1.053871
H	1.110411	3.250627	1.305393	H	2.056974	2.580297	-0.023963
H	1.476806	1.519120	1.287772	C	5.632847	-0.188922	1.768531
H	5.932892	-1.013668	2.410595	C	4.837327	1.919409	0.164684
H	4.533201	2.740974	-0.480449	C	1.784687	-1.566839	2.926088
O	1.407139	-2.693896	3.252218	O	1.031353	-0.563116	2.634716
C	4.471494	-1.178272	-5.469521	C	4.841712	-2.341202	-4.546814
C	5.376369	-1.895381	-3.185385	C	3.404913	-0.229115	-4.919424
N	4.316170	-1.515240	-2.236503	C	3.792679	0.464033	-3.598470
C	3.597705	-0.416361	-2.398967	H	5.379105	-0.599167	-5.685130
H	3.981428	-3.003166	-4.389871	H	6.079239	-1.063593	-3.297968
H	2.454763	-0.758863	-4.782155	H	3.184844	1.359916	-3.457556
H	4.123166	-1.579454	-6.426627	H	5.620556	-2.939981	-5.029547
H	5.925475	-2.707152	-2.707067	H	3.221565	0.557342	-5.656881
H	4.835804	0.797009	-3.632651	C	4.070105	-2.440864	-1.118625
C	2.364992	-0.821950	-0.312548	C	2.661856	-2.288850	-0.569946
H	1.317249	-0.659425	-0.053631	H	2.983026	-0.427651	0.500008
H	1.935389	-2.694175	-1.281180	H	2.578750	-2.858530	0.357699
H	4.815271	-2.246254	-0.340206	H	4.223624	-3.453732	-1.495773
S	-0.351442	-1.610478	5.663568	O	-1.255717	-0.818355	4.660956
C	-0.912039	-3.303598	5.480860	C	-1.104963	-1.186512	7.233850

In	-1.031551	-0.464216	2.586704	H	-0.602526	-3.612296	4.482322
H	-0.419011	-3.915604	6.238755	H	-1.997793	-3.332719	5.588240
H	-0.902385	-0.130326	7.414157	H	-2.178685	-1.374509	7.177191
H	-0.639279	-1.792784	8.013450	O	-1.399300	-2.456368	2.018077
O	-3.142803	-0.281496	2.688754	O	-1.060682	-0.024658	0.518728
O	-1.014746	1.612189	2.892745	S	-0.549722	-3.176609	0.922512
S	-4.055228	-1.447306	3.181894	S	-1.276055	1.347615	-0.200287
S	-0.863066	2.352508	4.259976	C	-1.727218	-3.435353	-0.404182
C	-0.394726	-4.844109	1.554603	C	-5.092265	-0.658926	4.414597
C	-5.235462	-1.624162	1.843162	C	-1.576279	0.826929	-1.890654
C	-2.936754	1.858641	0.254766	C	-0.938376	4.069752	3.751802
C	-2.469351	2.190721	5.046486	H	-1.988706	-2.445763	-0.779969
H	-1.240435	-4.019226	-1.188097	H	-2.606293	-3.952622	-0.016293
H	0.297305	-4.793263	2.395087	H	-1.378660	-5.198805	1.866828
H	0.016279	-5.472873	0.762314	H	-4.461688	-0.457715	5.280823
H	-5.888623	-1.352601	4.692225	H	-5.500330	0.266422	4.004302
H	-4.682399	-2.003918	0.983796	H	-5.675457	-0.650329	1.621477
H	-5.999189	-2.343835	2.143892	H	-0.664909	0.351328	-2.252840
H	-1.795773	1.715022	-2.486432	H	-2.412893	0.126704	-1.909454
H	-2.907706	2.108975	1.315110	H	-3.630479	1.037166	0.069727
H	-3.189583	2.738665	-0.340655	H	-0.047300	4.272623	3.157456
H	-0.938037	4.692887	4.647888	H	-1.841445	4.228901	3.160431
H	-2.567306	1.140647	5.321963	H	-3.249379	2.486526	4.343152
H	-2.475339	2.820082	5.938921	N	2.657412	-0.076631	-1.530445
H	2.166155	0.787829	-1.698495				

In-DBU-RC'

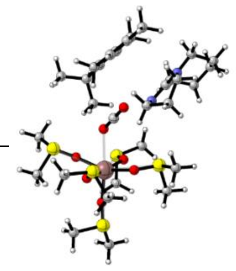

#p freq=noraman wb97xd/def2tzvp scrf=(solvent=dmso) nosymm geom=connec tivity gfinput iop(6/7=3)

Charge = 3, Multiplicity = 1, Point group = C1

Electronic Energy = -3997.56545790 Hartree

Sum of electronic and zero-point Energies = -3996.655441 Hartree

Sum of electronic and thermal Energies = -3996.596452 Hartree

Sum of electronic and thermal Enthalpies = -3861.644721 Hartree

Sum of electronic and thermal Free Energies = -3861.791972 Hartree

Atoms	Cartesian Coordinates			Atoms	Cartesian Coordinates		
	X	*Y*	*Z*		*X*	*Y*	*Z*
H	3.404168	0.807178	2.655159	C	6.110575	0.730264	-0.023145
C	6.173203	-0.150823	1.197527	C	4.390013	1.189096	2.352695
C	4.244548	1.998061	1.086694	H	7.093695	1.199300	-0.182924
H	4.686686	1.849654	3.180755	C	7.197383	-1.251809	1.160785
H	8.208517	-0.832878	1.086117	H	7.150668	-1.879786	2.054750
H	7.059425	-1.891514	0.280274	C	3.147196	3.045309	1.048817
H	3.304994	3.642006	0.141508	C	3.208297	3.998145	2.250231
H	2.476792	4.805288	2.134241	H	2.983594	3.475541	3.187622
H	4.199735	4.452087	2.349701	C	1.758446	2.398669	0.931222
H	0.982255	3.169047	0.860847	H	1.706328	1.768029	0.038133
H	1.525341	1.771480	1.800193	C	5.377804	0.060491	2.250693
H	5.455579	-0.594847	3.117248	C	5.039172	1.780589	0.034876
H	4.904414	2.380841	-0.864515	H	5.950730	0.100943	-0.910272
C	2.021143	-1.513220	3.080855	O	2.864287	-2.301771	3.155352
O	1.174316	-0.706317	3.004302	C	4.230271	-1.056944	-5.410116
C	4.897692	-2.056155	-4.462893	C	5.305193	-1.439977	-3.121139
C	2.979968	-0.371465	-4.852226	N	4.206279	-1.298054	-2.170045
C	3.215523	0.461344	-3.578702	C	3.224475	-0.356773	-2.309245
H	4.964131	-0.282109	-5.672493	H	4.231236	-2.906760	-4.269546
H	5.785781	-0.467810	-3.284484	H	2.200434	-1.118074	-4.650667
H	2.410761	1.188528	-3.455728	H	3.969922	-1.566822	-6.344586
H	5.795167	-2.457360	-4.947539	H	6.053999	-2.069691	-2.632953
H	2.580426	0.294843	-5.624324	H	4.149266	1.030850	-3.672105
N	2.279113	-0.134929	-1.452322	C	4.129126	-2.320178	-1.125904
C	2.301533	-0.924703	-0.234006	C	2.742234	-2.358882	-0.506931
H	1.298500	-0.892214	0.205601	H	2.992953	-0.458124	0.486190
H	2.032826	-2.828577	-1.198314	H	2.768441	-2.961838	0.405726
H	4.878179	-2.109601	-0.351466	H	4.376607	-3.289296	-1.573504
S	-0.687433	-2.348612	5.221614	O	-1.482425	-1.196433	4.502639

C	-1.836416	-3.722918	5.227611	C	-0.765847	-1.820331	6.929354
In	-1.411313	-0.618934	2.519399	H	-1.967923	-4.021676	4.186972
H	-1.383645	-4.537030	5.797188	H	-2.781443	-3.407987	5.672670
H	-0.169724	-0.910621	7.008273	H	-1.807019	-1.635345	7.198178
H	-0.332343	-2.607448	7.548916	O	-0.949793	-2.567427	2.064881
O	-3.449907	-0.509916	2.384093	O	-1.131660	-0.198161	0.541848
O	-1.048665	1.309851	3.051351	S	-0.684901	-3.191598	0.640009
S	-4.517253	-1.446861	3.052984	S	-1.348708	1.152063	-0.243095
S	-0.514342	1.868949	4.420494	C	-2.320471	-3.347220	-0.077424
C	-0.342063	-4.894099	1.071490	C	-4.959064	-0.585135	4.561105
C	-5.959301	-1.118320	2.044988	C	-0.802238	0.697183	-1.880898
C	-3.126972	1.217367	-0.479822	C	-0.277569	3.595531	4.013873
C	-1.979067	1.972562	5.450694	H	-2.671417	-2.336087	-0.285907
H	-2.226042	-3.907365	-1.010113	H	-2.978227	-3.858577	0.627346
H	0.594469	-4.898750	1.629500	H	-1.161676	-5.289435	1.672953
H	-0.224715	-5.457925	0.144025	H	-4.109253	-0.679659	5.238052
H	-5.833603	-1.079482	4.989186	H	-5.166742	0.461147	4.331146
H	-5.758869	-1.529408	1.055587	H	-6.122352	-0.040566	1.996122
H	-6.812244	-1.628099	2.497146	H	0.277915	0.492094	-1.816327
H	-0.992214	1.549412	-2.537324	H	-1.360219	-0.182936	-2.207131
H	-3.584628	1.338926	0.502919	H	-3.463166	0.293561	-0.952803
H	-3.346257	2.083251	-1.108000	H	0.529484	3.642682	3.282988
H	0.012103	4.122025	4.924972	H	-1.203980	3.996299	3.600276
H	-2.256917	0.949141	5.703231	H	-2.773671	2.474075	4.895880
H	-1.717227	2.523930	6.356180				

In-DBU-TS'

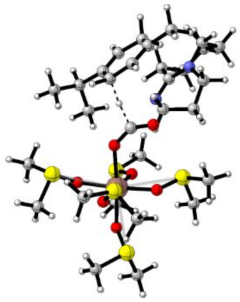

```
#p freq=noraman wb97xd/def2tzvp
scrf=(solvent=dmso) nosymm gfinput iop (6/7=3)
```

Charge = 3, Multiplicity = 1, Point group = C1

Electronic Energy = -3997.522293 Hartree

Number of imaginary frequencies = 1, vi = -813.16

Sum of electronic and zero-point Energies = -3996.616630 Hartree

Sum of electronic and thermal Energies = -3996.557674 Hartree

Sum of electronic and thermal Enthalpies = -3996.556730 Hartree

Sum of electronic and thermal Free Energies = -3996.709174 Hartree

	Cartesian Coordinates				Cartesian Coordinates		
Atoms	*X*	*Y*	*Z*	Atoms	*X*	*Y*	*Z*
H	2.879631	-0.126552	2.490601	C	5.717958	0.089163	0.248854
C	5.764302	-0.840483	1.416903	C	4.033485	0.482663	2.554322
C	4.012665	1.470139	1.460784	H	6.736167	0.431878	0.015608
H	3.852504	0.882169	3.553411	C	6.744277	-1.971275	1.350306
H	7.766911	-1.583893	1.283968	H	6.671458	-2.622142	2.223718
H	6.574803	-2.571651	0.449569	C	3.095458	2.680082	1.518066
H	3.559996	3.439210	0.878162	C	2.961329	3.277424	2.922405
H	2.427265	4.231064	2.871180	H	2.394525	2.618602	3.591058
H	3.938751	3.466813	3.377706	C	1.717691	2.357801	0.917473
H	1.129374	3.275618	0.809598	H	1.821065	1.892396	-0.068698
H	1.158093	1.672640	1.561915	C	4.984624	-0.622062	2.495354
H	5.043242	-1.278349	3.357949	C	4.817092	1.262016	0.402841
H	4.832947	1.992393	-0.403034	H	5.413270	-0.469839	-0.651376
C	1.863837	-0.969209	2.653664	O	2.233909	-2.098680	2.543332
O	0.871545	-0.272263	2.868090	C	4.439190	-0.553088	-5.618144
C	4.899859	-1.778625	-4.826660	C	5.282832	-1.455930	-3.379223
C	3.245419	0.193814	-5.016865	N	4.144903	-1.324066	-2.471987
C	3.485579	0.765196	-3.607295	C	3.298841	-0.244946	-2.501122
H	5.282798	0.146340	-5.700695	H	4.116175	-2.547357	-4.820170
H	5.890112	-0.544218	-3.342807	H	2.365978	-0.463109	-4.990530
H	2.770753	1.563968	-3.401338	H	4.188815	-0.859358	-6.640073
H	5.770880	-2.216842	-5.326950	H	5.907853	-2.254393	-2.969288
H	2.991356	1.026967	-5.680798	H	4.487102	1.211098	-3.550777
N	2.343140	-0.038653	-1.652892	C	3.807382	-2.532965	-1.715091
C	2.208079	-1.021835	-0.592620	C	2.414979	-2.439411	-1.113786
H	1.217724	-0.910797	-0.138032	H	2.953155	-0.819138	0.191177
H	1.653384	-2.655299	-1.871792	H	2.314750	-3.179024	-0.315254
H	4.552485	-2.679247	-0.920985	H	3.879066	-3.395600	-2.387836

S	-0.528843	-1.827716	5.468528	O	-1.467454	-0.876818	4.641965
C	-1.521746	-3.303387	5.687702	C	-0.624488	-1.103045	7.102623
In	-1.302536	-0.498848	2.597273	H	-1.630555	-3.750993	4.698861
H	-0.981825	-3.982234	6.351006	H	-2.491509	-3.031635	6.107597
H	-0.124489	-0.135367	7.050244	H	-1.672955	-0.987925	7.382284
H	-0.098706	-1.758555	7.799266	O	-1.019305	-2.535245	2.315681
O	-3.375791	-0.605911	2.447716	O	-1.098078	-0.215360	0.562143
O	-1.414368	1.538211	2.925729	S	-0.506454	-3.232028	1.006146
S	-4.252503	-1.673107	3.184407	S	-1.388913	1.093272	-0.259972
S	-0.889660	2.283062	4.200762	C	-2.003325	-3.525925	0.064356
C	-0.146663	-4.883460	1.593315	C	-4.988467	-0.753612	4.536847
C	-5.653691	-1.827918	2.080218	C	-0.843497	0.628920	-1.897414
C	-3.170898	1.064811	-0.474195	C	-0.851421	3.980888	3.634018
C	-2.313757	2.329404	5.290174	H	-2.373492	-2.546905	-0.242703
H	-1.741731	-4.119835	-0.813930	H	-2.730721	-4.043792	0.691736
H	0.713529	-4.802747	2.258411	H	-1.017575	-5.275213	2.121155
H	0.103561	-5.503993	0.730557	H	-4.183993	-0.547243	5.243615
H	-5.748314	-1.380117	5.008321	H	-5.420576	0.170903	4.150360
H	-5.283975	-2.289204	1.164144	H	-6.063750	-0.836849	1.880094
H	-6.394392	-2.476049	2.552568	H	0.251488	0.541618	-1.872286
H	-1.142355	1.428140	-2.579630	H	-1.314310	-0.317485	-2.171453
H	-3.615549	1.187591	0.514434	H	-3.467778	0.111804	-0.914864
H	-3.445338	1.901554	-1.119767	H	-0.079325	4.041822	2.866945
H	-0.588516	4.618724	4.479889	H	-1.827516	4.245752	3.224629
H	-2.476549	1.306161	5.629803	H	-3.177601	2.703287	4.738484
H	-2.073885	2.973709	6.138574				

10. Effect of $In(OTf)_3$ on the ^{13}C NMR of CO_2 in DMSO-d6

In a pressure NMR tube CO2 (5 bar) was let to dissolve in DMSO-d6 (0.02 mL) for 20 minutes with occasional shaking. After the 20 minutes the NMR tube was sealed and a 13C NMR measured (Figure S26).

^{13}C NMR (DMSO-d6): 124.6 ppm (s, CO_2)

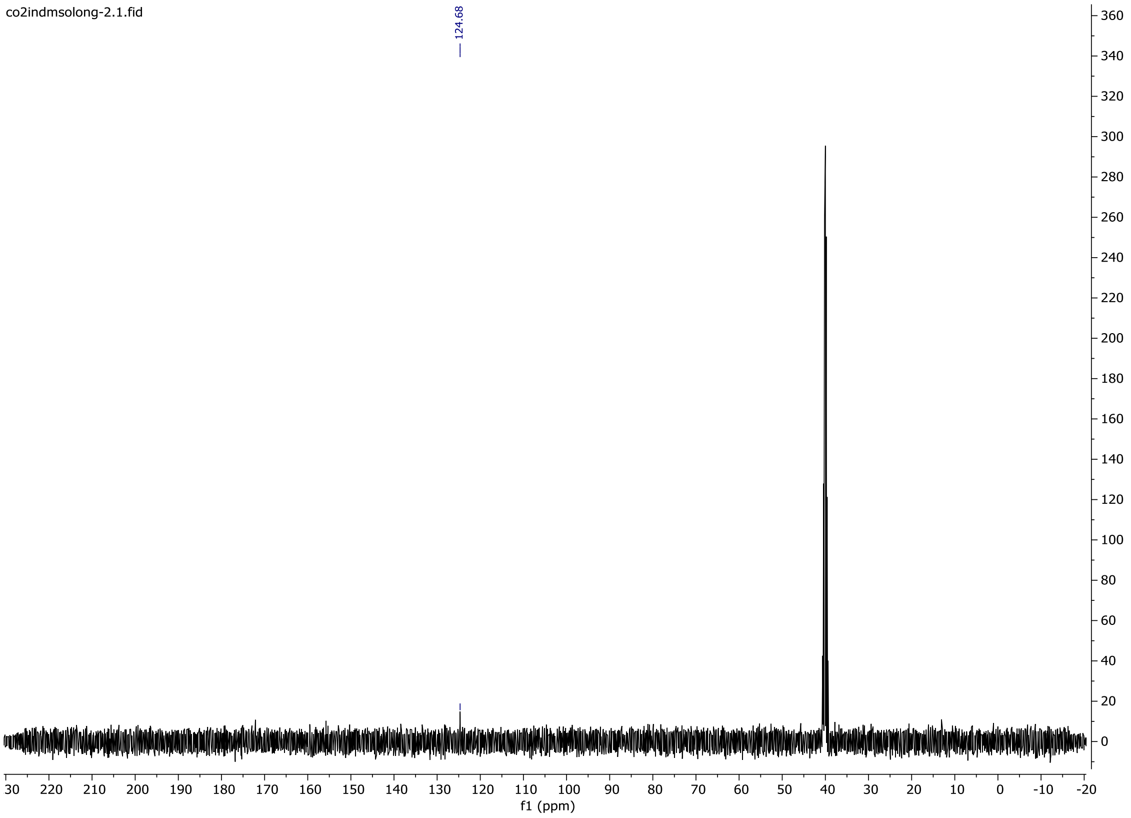

Figure S26: ^{13}C NMR of CO_2 in DMSO-d6; 128 scans measured

The measurement was repeated including $In(OTf)_3$ (2.5 mg) (Figure S27). While $In(OTf)_3$ does not interact strongly enough with CO_2 on the NMR time scale to affect changes in the chemical shift of the CO_2 signal, which in both presence and absence of $In(OTf)_3$ appears at 125 ppm, it strongly affects CO_2 solubility greatly increasing its concentration in solution.

^{13}C NMR (DMSO-d6): 124.6 ppm (s, CO_2), 121.0 (q, $J_{C\text{-}F}$ =340 Hz, -CF_3 of OTf)

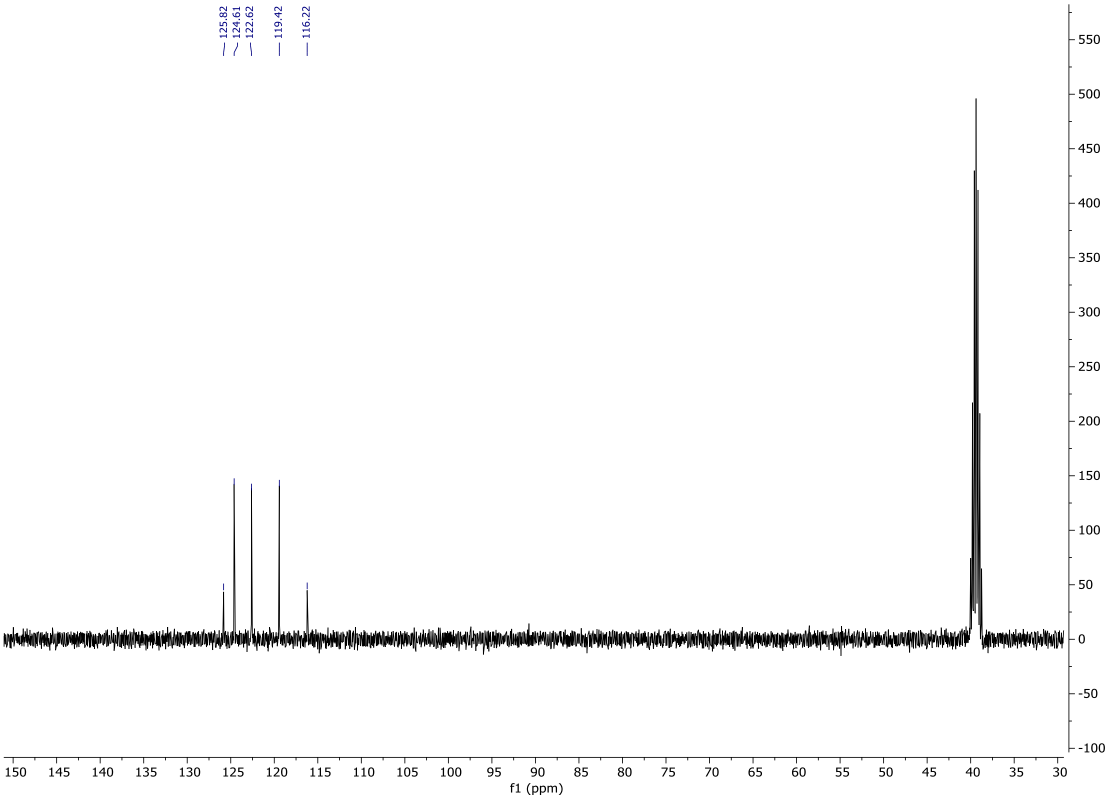

Figure S27: ^{13}C NMR of CO_2 in the presence of $In(OTf)_3$; 32 scans measured

References

1. Gaussian 16, Revision C.01, M. J. Frisch, G. W. Trucks, H. B. Schlegel, G. E. Scuseria, M. A. Robb, J. R. Cheeseman, G. Scalmani, V. Barone, G. A. Petersson, H. Nakatsuji, X. Li, M. Caricato, A. V. Marenich, J. Bloino, B. G. Janesko, R. Gomperts, B. Mennucci, H. P. Hratchian, J. V. Ortiz, A. F. Izmaylov, J. L. Sonnenberg, D. Williams-Young, F. Ding, F. Lipparini, F. Egidi, J. Goings, B. Peng, A. Petrone, T. Henderson, D. Ranasinghe, V. G. Zakrzewski, J. Gao, N. Rega, G. Zheng, W. Liang, M. Hada, M. Ehara, K. Toyota, R. Fukuda, J. Hasegawa, M. Ishida, T. Nakajima, Y. Honda, O. Kitao, H. Nakai, T. Vreven, K. Throssell, J. A. Montgomery, Jr., J. E. Peralta, F. Ogliaro, M. J. Bearpark, J. J. Heyd, E. N. Brothers, K. N. Kudin, V. N. Staroverov, T. A. Keith, R. Kobayashi, J. Normand, K. Raghavachari, A. P. Rendell, J. C. Burant, S. S. Iyengar, J. Tomasi, M. Cossi, J. M. Millam, M. Klene, C. Adamo, R. Cammi, J. W. Ochterski, R. L. Martin, K. Morokuma, O. Farkas, J. B. Foresman, and D. J. Fox, Gaussian, Inc., Wallingford CT, **2016**.
2. Pérez-Guevara, R., Sarandeses, L. A., Álvarez, R., Martínez, M. M., & Pérez Sestelo, J. (2024). Advanced Synthesis & Catalysis, 366(4), 852-861. https://doi.org/10.1002/adsc.202301329
3. Nava, P.; Carissan, Y.; Humbel, S. Labile Ligands on Some Lewis Super Acids: A Computational Study. Phys. Chem. Chem. Phys. **2009**, 11 (33), 7130–7136. https://doi.org/10.1039/b907229b
